# An integrated map of HIV genome-wide variation from a population perspective

**DOI:** 10.1186/s12977-015-0148-6

**Published:** 2015-02-15

**Authors:** Guangdi Li, Supinya Piampongsant, Nuno Rodrigues Faria, Arnout Voet, Andrea-Clemencia Pineda-Peña, Ricardo Khouri, Philippe Lemey, Anne-Mieke Vandamme, Kristof Theys

**Affiliations:** Metabolic Syndrome Research Center, the Second Xiangya Hospital, Central South University, Changsha, Hunan China; Rega Institute for Medical Research, Department of Microbiology and Immunology, KU Leuven, Leuven, Belgium; Department of Zoology, University of Oxford, Oxford, OX1-3PS UK; Zhang IRU, RIKEN Institute Laboratories, Hirosawa 2-1, Wako-shi, Saitama Japan; Clinical and Molecular Infectious Disease Group, Faculty of Sciences and Mathematics, Universidad del Rosario, Bogotá, Colombia; LIMI-LIP, Centro de Pesquisa Gonçalo Moniz, FIOCRUZ, Salvador-Bahia, Brasil; Centro de Malária e Outras Doenças Tropicais and Unidade de Microbiologia, Instituto de Higiene e Medicina Tropical, Universidade Nova de Lisboa, Lisbon, Portugal

**Keywords:** HIV genome, Genomic diversity, Conservation, Peptide inhibitor, HIV-human protein interaction, HIV phylogenetic tree, HIV inter- and inter-clade genetic diversity, Selective pressure, Protein multimerization, Protein intrinsic disorder

## Abstract

**Background:**

The HIV pandemic is characterized by extensive genetic variability, which has challenged the development of HIV drugs and vaccines. Although HIV genomes have been classified into different types, groups, subtypes and recombinants, a comprehensive study that maps HIV genome-wide diversity at the population level is still lacking to date. This study aims to characterize HIV genomic diversity in large-scale sequence populations, and to identify driving factors that shape HIV genome diversity.

**Results:**

A total of 2996 full-length genomic sequences from 1705 patients infected with 16 major HIV groups, subtypes and circulating recombinant forms (CRFs) were analyzed along with structural, immunological and peptide inhibitor information. Average nucleotide diversity of HIV genomes was almost 50% between HIV-1 and HIV-2 types, 37.5% between HIV-1 groups, 14.7% between HIV-1 subtypes, 8.2% within individual HIV-1 subtypes and less than 1% within single patients. Along the HIV genome, diversity patterns and compositions of nucleotides and amino acids were highly similar across different groups, subtypes and CRFs. Current HIV-derived peptide inhibitors were predominantly derived from conserved, solvent accessible and intrinsically ordered structures in the HIV-1 subtype B genome. We identified these conserved regions in Capsid, Nucleocapsid, Protease, Integrase, Reverse transcriptase, Vpr and the GP41 N terminus as potential drug targets. In the analysis of factors that impact HIV-1 genomic diversity, we focused on protein multimerization, immunological constraints and HIV-human protein interactions. We found that amino acid diversity in monomeric proteins was higher than in multimeric proteins, and diversified positions were preferably located within human CD4 T cell and antibody epitopes. Moreover, intrinsic disorder regions in HIV-1 proteins coincided with high levels of amino acid diversity, facilitating a large number of interactions between HIV-1 and human proteins.

**Conclusions:**

This first large-scale analysis provided a detailed mapping of HIV genomic diversity and highlighted drug-target regions conserved across different groups, subtypes and CRFs. Our findings suggest that, in addition to the impact of protein multimerization and immune selective pressure on HIV-1 diversity, HIV-human protein interactions are facilitated by high variability within intrinsically disordered structures.

**Electronic supplementary material:**

The online version of this article (doi:10.1186/s12977-015-0148-6) contains supplementary material, which is available to authorized users.

## Background

As the causative agent of AIDS, the Human Immunodeficiency Virus (HIV) represents a worldwide threat to public health and the economy. The HIV pandemic is characterized by extensive genomic diversity caused by multiple factors including multiple zoonotic transmissions into human populations, high rates of viral evolution and recombination [1]. HIV has two major types, HIV-1 and HIV-2, which are further divided into groups, subtypes and recombinant forms. Globally, over 90% of HIV infections belong to HIV-1 group M viruses, which have been classified into 9 subtypes (A-D, F-H, J, K) and more than 50 circulating recombinant forms (CRFs) [1]. The high genetic diversity of the HIV genome has challenged the development of drugs and vaccines [2].

The HIV genome contains nine genes that encode fifteen viral proteins (Additional file 1: Figure S1). Three major genes, *gag*, *pol* and *env*, code for structural proteins (Matrix, Capsid, Nucleocapsid, p6), enzymes (Protease, Reverse transcriptase (RT), Integrase) and envelope proteins (GP120, GP41), respectively. The remaining genes code for regulatory (Tat, Rev) and accessory proteins (Vif, Vpr, Vpu/Vpx, Nef) [3]. These viral proteins can exhibit multiple functions and interact with various human proteins during the viral life cycle [4,5].

During the past three decades, many antiviral inhibitors have been designed to prevent HIV replication by targeting different viral proteins [6]. These anti-HIV peptides and small-molecule inhibitors either act by blocking active sites of viral enzymes or interrupting protein interactions [6]. For instance, the fusion inhibitor T20 (Enfuvirtide, Fuzeon), a peptide derived from the GP41 heptad repeat region, can efficiently inhibit viral entry by interrupting interactions between the GP41 helices [7]. For all existing drug classes, mutations in the HIV genome can cause drug resistance [8]. Therefore, inhibitors have been preferentially developed to target conserved regions of different viral proteins [9]. HIV genetic diversity also challenges the development of a global HIV vaccine [10]. While the vaccine trial STEP was unable to show preventive vaccination in subtype B infected cohorts [11], the Thai trial RV144 showed for the first time that prime-boost vaccination provided a modest efficacy in patients infected with CRF01_AE [12]. For vaccine and drug design, it remains important to investigate the genomic diversity of different HIV groups, subtypes and CRFs at a population level.

Despite a large body of knowledge on different aspects of HIV pathogenesis, a large-scale analysis that reveals the genome-wide diversity within and between different HIV groups, subtypes and CRFs is still lacking. Although previous HIV genomic studies have reported subtype distribution, genetic variability, disease progression, evolutionary rate, positive selective pressure and the origin of HIV [11-27], most studies reported their findings using either reference genomes or small cohorts of less than 100 patients or sequences in a single subtype. HIV-1 subtype B which dominates infections in developed countries is the most studied subtype, largely due to historical reasons [28]. For instance, the adaptive evolution during acute infection was evaluated only in 11 individuals infected with HIV-1 subtype B [14]. In light of using HIV consensus sequences as vaccine candidates, an analysis on the genetic difference between consensus sequences and circulating strains was limited to subtypes B and C using less than 100 sequences [2], while other subtypes also prevail worldwide [29].

The last three decades have seen an accumulation of HIV data including full-length genomic sequences, protein crystal structures, HIV-human protein interactions, human T-cell epitope information and antiretroviral peptide inhibitors derived from the HIV genome. By integrating distinct but complementary sources of large-scale HIV datasets, this study aims to characterize HIV genome-wide diversity and to determine multiple factors that shape HIV genomic diversity.

## Results

### Genome-wide diversity within and across HIV types, major groups and subtypes

**Figure 1:**
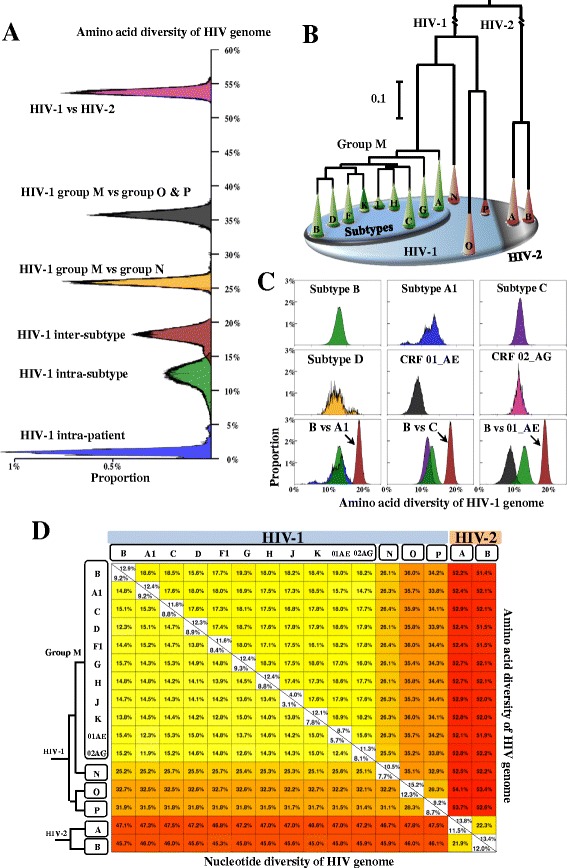
**Distribution of HIV genome-wide diversity and phylogenetic tree. (A)** Distribution plots of amino acid diversity in the HIV genome. The plots show the genomic diversity within HIV-1 infected patients (HIV-1 intra-patient, blue), within HIV-1 subtypes (HIV-1 intra-subtype, green), between HIV-1 subtypes (HIV-1 inter-subtype, red), between HIV-1 group M and group N (HIV-1 inter-group, yellow), between HIV-1 group M and group O/P (HIV-1 inter-group, black) and between HIV-1 and HIV-2 (pink). Distribution plots of nucleotide genomic diversity are shown in Additional file 1: Figure S2. **(B)** Maximum likelihood phylogenetic tree of HIV groups and pure subtypes. Green cones indicate HIV-1 subtypes in group M, while orange cones denote other HIV groups. All phylogenetic branches have bootstrap supports of more than 85% except one containing subtypes J, H and C. Branch lengths from the root to HIV-1 and HIV-2 are shortened for visualization purposes. SIV strains were not included in our phylogenetic tree. Visualization software: FigTree V1.4.0 (http://tree.bio.ed.ac.uk/software/figtree/). **(C)** Distribution plots of amino acid diversity in 6 major HIV-1 subtypes and CRFs (B, A1, C, D, CRF01_AE, CRF02_AG). X- and y-axes indicate the amino acid diversity and the proportions of sequence pairs, respectively. Six subplots in the first and second rows show the intra-subtype amino acid diversity of 6 HIV-1 subtypes and CRFs. Three subplots in the third row show the distribution of inter-subtype genomic diversity (B vs A1, B vs C, B vs 01_AE). One genomic sequence per patient (Table 1) was used for our analysis. Distribution plots of the other inter-clade genomic diversity are shown in Additional file 1: Figure S3. **(D)** Average inter- and intra-clade genomic diversity of HIV-1 and HIV-2. The top right matrix demonstrates results for amino acid diversity, the bottom left matrix for nucleotide diversity. HIV subtypes and groups are shown on the left side of the matrix.

We quantified the nucleotide and amino acid diversity of the HIV genome using 2996 full-length sequences sampled from 1705 patients (Table 1). The amino acid diversity was 53.8% (95% confidence interval (CI): 53.0-54.6%) between HIV-1 and HIV-2, 41.1% (CI: 25.6-54.3%) between HIV-1 groups, 18.0% (CI: 15.6-19.6%) between HIV-1 subtypes, 12.0% (CI: 8.6-14.4%) within HIV-1 subtypes and 1.1% (CI: 0.3-2.2%) within HIV-1 patients (Figure 1A). Similarly, nucleotide genomic diversity was found to be the highest when comparing HIV-1 and HIV-2 (mean: 48.32%, CI: 47.8-48.9%), followed by HIV-1 inter-group (37.5%, CI: 26.0-45.7%), HIV-1 inter-subtype (14.7%, CI: 12.2-15.8%), HIV-1 intra-subtype (8.2%, CI: 5.3-10.0%) and HIV-1 intra-patient diversity (0.6%, CI: 0.2-1.4%) (Additional file 1: Figure S2). As expected, the trend in HIV genomic diversity corresponds with the phylogenetic relationships between groups and pure subtypes in HIV-1 and HIV-2 (Figure 1B).

**Table 1:** **Information on HIV-1 and HIV-2 full-length genomic sequence datasets**

**Type**	**HIV-1**														**HIV-2**	
**Group**	**M**											**N**	**O**	**P**	**A**	**B**
**Subtype/CRF**	**A1**	**B**	**C**	**D**	**F1**	**G**	**H**	**J**	**K**	**01_AE**	**02_AG**					
Number of genomes	159	1425	554	65	25	27	4	2	2	581	81	11	25	4	25	6
Number of patients	134	657	429	57	22	23	4	2	2	250	71	9	22	2	16	5
Average length in nucleotides*	8500	8600	8600	8500	8500	8600	8600	8600	8600	8500	8500	8500	8700	8600	8600	8600

*: Only the HIV coding regions are counted.

**Figure 2:**
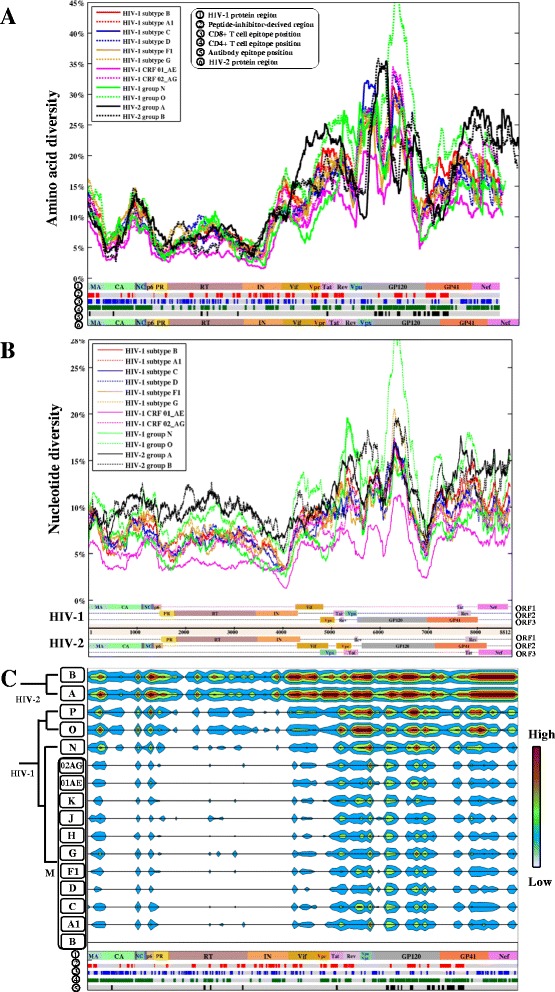
**Plots of amino acid and nucleotide diversity in the HIV full-length genome. (A)** Amino acid diversity along the HIV full-length genome using the sliding windows (window size: 100AA; also see the plots of exact diversity values in Additional file 1: Figure S5). Each colored plot shows the density of amino acid diversity for one HIV group, subtype or CRF genome, indicated by the figure legend. Six layers are shown beneath the plots: (1) HIV-1 protein regions (HXB2 reference) are concatenated and shown with abbreviated names (e.g. MA: matrix); (2) peptide-inhibitor-derived region; (3) CD8+ T cell epitope position; (4) CD4+ T cell epitope position; (5) antibody epitope position; (6) HIV-2 protein region (BEN reference). **(B)** Nucleotide diversity along the full-length HIV genome using sliding windows (window size: 300 nucleotides; also see the plots of exact diversity values in Additional file 1: Figure S6). Each colored plot shows the density of nucleotide diversity for one HIV group, subtype or CRF genome, indicated by the figure legend. Annotated HIV-1 and HIV-2 reference genomes are shown beneath; each track contains one open reading frame (ORF). Long terminal regions in the HIV genome are not shown. **(C)** Contour map of inter-clade amino acid diversity between HIV-1 subtype B and the other HIV genomes. Inter-clade amino acid diversity was calculated by a sliding window of 30 amino acids over the HIV genome (low: ≤1 AA difference, high: ≥25 AA differences). Five colored layers beneath the contour map are annotated similarly in **(A)**.

We next quantified genomic diversity within and between individual HIV clades (Figure 1C, Additional file 1: Figure S3). Within each HIV clade, amino acid diversity was consistently higher than nucleotide diversity (Figure 1D). CRF01_AE showed the lowest genomic diversity (nucleotide: 5.7%, amino acid: 8.7%) among the 10 HIV-1 subtypes with at least 10 sequences available (Figure 1D). Sequence variability was not uniformly distributed along the full-length HIV genome, but similar patterns were consistently observed in HIV group, subtype and CRF genomes at the nucleotide and amino acid levels (Figure 2A, B). Moreover, the estimated geographical distribution of HIV-1 genomic diversity (Additional file 1: Figure S4) showed a good agreement with the reported geographical distribution of HIV-1 subtypes [29].

**Figure 3:**
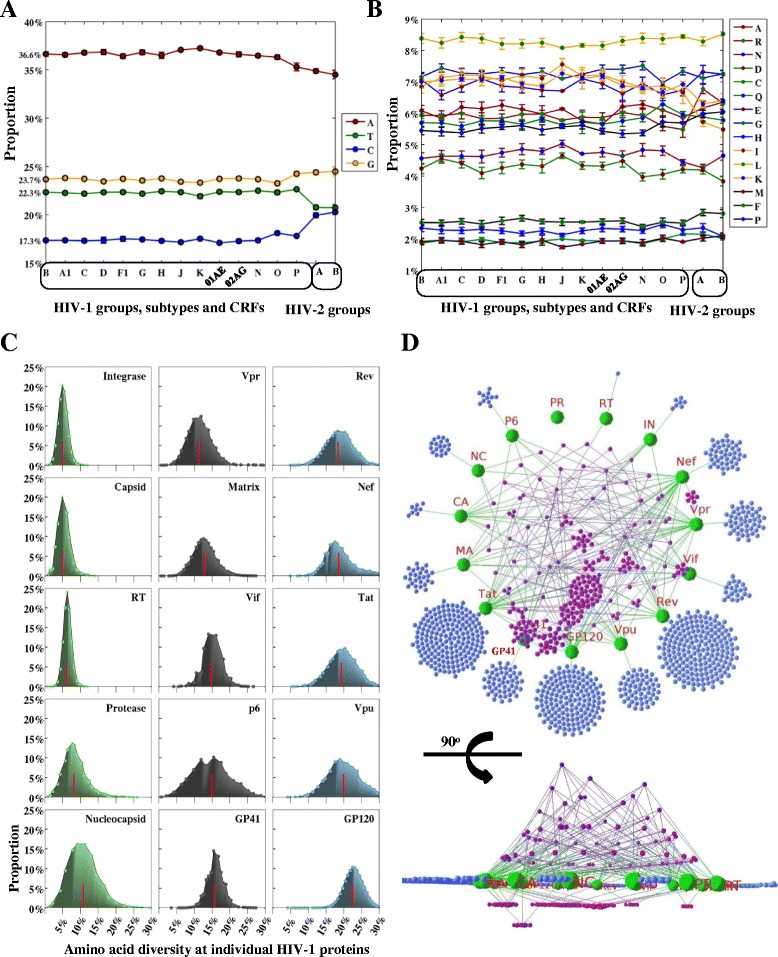
**Nucleotide and amino acid composition of HIV genomes and 3D mapping of HIV-human protein interactions. (A)** Nucleotide composition for HIV-1 and HIV-2. X-axis represents the HIV groups, subtypes and CRFs. Y-axis shows the average proportions of nucleotides (A, T, C, G) using the HIV genomic sequence datasets (one sequence per patient, Table 1). **(B)** Amino acid composition for HIV-1 and HIV-2. X-axis represents HIV groups, subtypes and CRFs. Y-axis shows the average proportions of amino acids using the HIV protein sequence datasets (one sequence per patient, Table 1). **(C)** Distribution plots of amino acid genetic diversity for 15 HIV-1 subtype B proteins. Each subplot demonstrates a viral protein. X- and y-axes indicate the amino acid diversity and the proportions of amino acid diversity, respectively. Red lines inside the distribution plots indicate the mean values of amino acid diversity at individual proteins. **(D)** Top and side views of 3D HIV-human protein interaction networks. HIV-1 proteins with protein names annotated are indicated by green spheres. Human proteins that interact with only one HIV-1 protein are indicated by blue spheres in the outer circle (one human protein one sphere). Human proteins that interact with more than one HIV-1 protein are indicated by purple spheres above the plane of HIV-1 proteins. The height of the layers above the plane indicates the number of HIV proteins that a human protein interacts with. Below, human proteins are clustered if they interact with a set of more than one HIV-1 protein. Abbreviation names have been described in the abbreviation list. Visualization software: Geomi V2.0(http://sydney.edu.au/engineering/it/~visual/geomi2/).

Among all HIV proteins, Integrase was the most conserved protein (mean ± deviation: 4.5 ± 1.1%), while GP120 varied the most (21.3 ± 2.5%) (Table 2). Pairwise comparisons of genetic diversity between subtype B and the other clades identified conserved regions in the Capsid, Nucleocapsid, Protease, RT, Integrase, Vpr and the N terminus of GP41 (Figure 2C). Despite the different degrees of sequence diversity along the full-length genome, the nucleotide and amino acid compositions were comparable across the 16 group and subtype genomes (Figure 3A, 3B).

**Table 2:** **Average amino acid diversity of viral proteins within individual HIV clades (%)**

	**Clade #**	**MA**	**CA**	**NC**	**p6**	**PR**	**RT**	**IN**	**Vif**	**Vpr**	**Tat**	**Rev**	Vpu/Vpx*	**GP120**	**GP41**	**Nef**
**HIV-1**	**Subtype A1**	13.07	7.3	7	19.89	5.59	6.44	4.76	13.63	9.86	18.14	16.83	23.04	23.34	13.38	14.64
	**Subtype B**	12.92	4.95	10.83	14.99	8.21	6.04	4.91	14.71	11.29	19.32	18.34	20.04	23.93	15.59	17.79
	**Subtype C**	16.11	5.77	9.39	15.52	6.15	5.84	4.45	10.61	10.64	15.37	16.10	20.21	22.89	14.19	14.96
	**Subtype D**	14.78	5.08	10.85	13.16	7.68	6.98	4.80	11.57	11.74	17.89	15.87	19.40	23.58	13.61	14.42
	**Subtype F1**	13.69	5.71	10.07	16.72	8.84	6.42	4.76	11.04	9.26	17.01	15.70	18.27	20.24	13.39	14.02
	**Subtype G**	17.33	5.54	7.40	17.21	6.60	7.30	4.17	14.36	10.33	15.51	18.39	19.25	23.87	12.98	14.15
	**Subtype H**	18.81	3.61	8.48	17.65	4.55	5.95	5.71	15.46	9.11	19.28	19.80	18.31	21.51	12.33	16.34
	**CRF01_AE**	10.06	3.00	4.92	13.37	4.34	3.75	2.39	9.32	7.97	13.57	13.34	12.80	17.40	9.62	11.78
	**CRF02_AG**	13.57	5.69	3.78	13.63	5.56	5.67	4.06	13.44	7.70	13.58	15.49	16.42	23.37	12.09	15.45
	**Group N**	10.52	6.40	7.06	10.01	5.93	6.78	3.09	11.02	4.87	12.15	12.77	35.01	21.43	9.15	12.64
	**Group O**	14.52	7.45	9.89	22.17	8.20	6.51	5.77	16.17	12.16	21.61	21.25	27.12	28.65	19.35	17.96
**HIV-2**	**Group A**	10.15	4.67	11.82	11.02	7.14	6.89	6.78	12.74	22.55	22.59	22.75	10.40	18.55	16.47	21.98
	**Group B**	12.14	3.54	13.15	15.4	5.76	6.4	6.19	12.71	12.66	20.44	15.82	11.45	21.20	15.29	20.59

#: Only HIV groups, subtypes or CRFs with more than 2 genomic sequences are listed.

*: Vpu in HIV-1 and Vpx in HIV-2.

### Multiple factors shape HIV-1 genomic diversity

We next evaluated three potential factors (protein multimerization, immunological constraints, HIV-human protein interactions) that shaped the HIV genomic diversity. Firstly, we calculated the average diversity at amino acid positions of the 15 HIV-1 proteins (Figure 3C). For every HIV-1 group, subtype and CRF, the average amino acid diversity was significantly higher in the monomeric proteins (Nucleocapsid, Vpr, Vpu, p6) than in the multimeric proteins (Matrix, Capsid, Protease, RT, Integrase, Vif, Tat, Rev, GP120, GP41, Nef) (p-value < 0.05) (Additional file 2: Table S1). This suggests that protein multimerization imposes a constraint on the HIV-1 amino acid variability. Besides our diversity analysis, we also measured synonymous (dS) and non-synonymous (dN) substitution rates in the HIV-1 subtype A1, B, C and CRF01_AE genomes, which contained more than 100 sequences in our genomic datasets (Additional file 2: Table S2). Mann-Whitney U tests were used to compare the distribution of dN, dS and the ratio of non-synonymous to synonymous substitutions (dN/dS) between the monomeric and multimeric protein groups. We found that the distributions of dN/dS and dN in these two groups were significantly different (p-value < 0.05) in HIV-1 subtypes A1, B, C and CRF01_AE (Additional file 2: Table S3). This suggests that multimeric proteins are under the stronger negative selective pressure than monomeric proteins.

Secondly, we evaluated the amino acid variation in the known CD4 T cell, CD8 T cell and antibody epitopes (see Materials). By measuring the diversity of 3066 amino acid positions, we identified 919 (30%) variable positions with amino acid diversity above 12.9% (the average amino acid diversity within subtype B) using 657 subtype B genomic sequences. Univariate analysis showed that these variable positions were preferably located within antibody epitopes (OR 1.43, CI: 1.15-1.79, Fisher’s exact test, p-value = 0.0015) and CD4 T cell epitopes (OR 1.73, CI: 1.18-2.96, p-value = 0.0438), but not within CD8 T cell epitopes (OR 1.11, CI: 0.82-1.51, p-value = 0.498) (Figure 2A).

**Figure 4:**
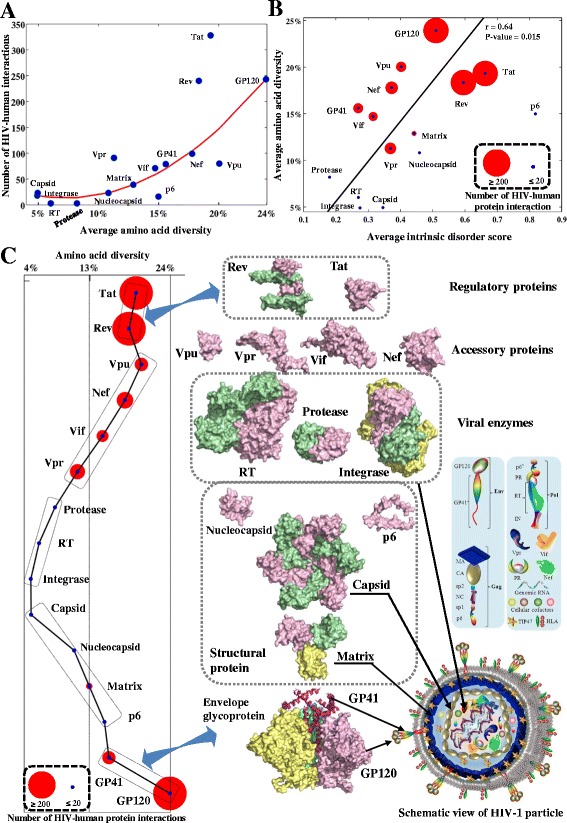
**Correlations between HIV-1 protein diversity and HIV-human protein interactions, protein disorder and viral particle structures. (A)** Plot of polynomial regression between the HIV-1 protein diversity (x-axis) and the number of HIV-human protein interactions (y-axis). The second-order model is $\mathcal{Y}=8346{\mathcal{X}}^2-1223\mathcal{X}+57.96$ (adjusted R-squared: 0.82, root-mean-square error: 42.31). **(B)** Plot of average protein disorder score and average amino acid diversity in HIV-1 proteins. Red circles indicate the number of HIV-human protein interactions at individual viral proteins, for visualization purpose, scaled between 20 and 200 interactions (proteins with fewer than 20 interactions are scaled to the same size as those with 20, proteins with more than 200 interactions are scaled to the same size as those with 200). Average amino acid diversities of HIV-1 proteins are calculated using subtype B sequences (one genomic sequence per patient, Table 1). **(C)** Clustering of HIV-1 proteins and schematic view of HIV-1 viral particle. On the left, each colored circle represents a viral protein positioned according to the clusters of protein functions. The size of each red circle indicates the number of HIV-human protein interactions involving each HIV-1 protein (see **(B)**). On the right, the schematic view of mature viral particle is visualized at the bottom with annotations indicated in the inserted figure legend. Above, surface representations show the structures of HIV-1 proteins that are grouped according to their functional roles. Different units in HIV-1 multimeric proteins are indicated with different colors and HIV-1 monomeric proteins are colored pink. HIV-1 protein structures are scaled according to their precise protein sizes for direct comparison. Visualization software: PyMOL V1.5 (http://www.pymol.org/).

Thirdly, we mapped 1352 interactions between 1052 human and 15 HIV-1 proteins using the HIV-human protein interaction dataset (Figure 3D, see Materials). The following three observations support the hypothesis that the amino acid diversity of HIV-1 proteins is associated with HIV-human protein interactions. (1) Univariate analysis showed that HIV-1 proteins with higher amino acid diversity interact with more human proteins (Pearson’s coefficient = 0.74, p-value = 0.0017). Polynomial regression analysis further identified a second-order model that fitted the correlation between these two variables (Figure 4A, adjusted R-squared: 0.82). (2) Intrinsically disordered structures in HIV-1 proteins can interact with multiple interaction partners [30]. Univariate analysis showed a significant correlation between the average amino acid diversity and the average disorder scores of HIV-1 proteins (Pearson’s coefficient = 0.64, p-value = 0.015, Figure 4B). (3) The levels of HIV-human protein interactions clustered according to the functional roles of the HIV-1 proteins, which have different functional roles and requirements for interactions with human proteins (Figure 4C). HIV regulatory proteins (Tat, Rev) and envelope proteins (GP120, GP41) had the largest number of interactions with different human proteins (568 for the regulatory proteins, 322 for the envelope proteins), while viral enzymes had the least number of interactions (Figure 4C). The average amino acid diversity of envelope proteins (20.4%) and regulatory proteins (18.8%) was higher than that of accessory proteins (16.0%), structural proteins (9.0%) and viral enzymes (5.9%) (Additional file 1: Figure S7). Our findings suggest that HIV-1 proteins with higher genetic diversities have larger intrinsically disordered structures and interact with more human proteins.

### Peptide inhibitors are mainly derived from conserved subtype B genomic regions

**Figure 5:**
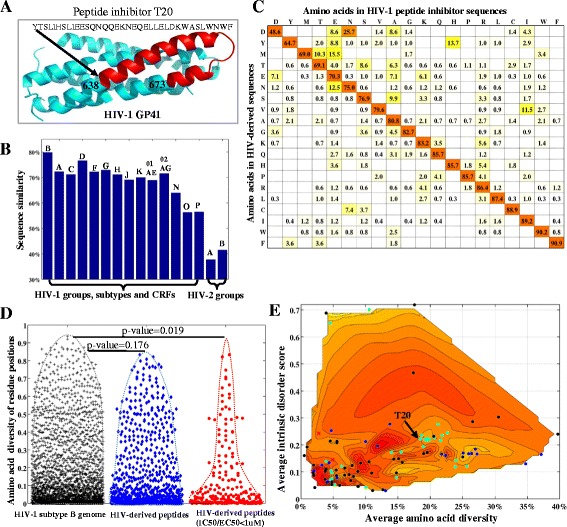
**Characterization of HIV-derived peptide inhibitors. (A)** Cartoon representation of GP41 structure. The red structure indicates the region from which peptide inhibitor T20 was derived (PDB: 3H01). **(B)** Bar plot of sequence similarities between peptide inhibitor sequences and the sequences of HIV-derived regions in the consensus genome of different HIV clades. X-axis presents the HIV groups, subtypes and CRFs. Y-axis shows the sequence similarity between peptide inhibitor sequences and the sequences of HIV-derived regions in the consensus genomes of HIV groups, subtypes or CRFs. **(C)** Amino acid replacements between peptide inhibitor sequences and HIV-derived regions in the subtype B genome. The percentage values (%) are colored using heat maps. **(D)** Distribution (bee-swarm) plots of amino acid diversity in the full-length subtype B genome (black crosses), peptide-derived regions (blue diamonds) and peptide-derived regions of those inhibitors whose IC_50_/EC_50_ are less than 1 μM (red circles). Each shape represents the amino acid diversity at one protein position. Two-sample Kolmogorov-Smirnov tests were performed to compare diversity distributions (significance level: 0.05). **(E)** Plot of amino acid diversity (x-axis), disorder score (y-axis) and solvent accessible surface area of peptide-inhibitor-derived regions (contour map, darker red indicates larger accessible surface areas). GP41 inhibitor T20 is also annotated. For individual peptide inhibitors, the average amino acid diversity, disorder score and solvent accessible surface areas are shown in Additional file 1: Figure S9, S10 and S11, respectively.

We investigated the 121 HIV-derived peptide inhibitors reported between 1993 and 2013 (Additional file 2: Table S4). Figure 5A illustrates the GP41 structure and the GP41-derived region of T20 as an example of HIV-derived peptide inhibitors. Peptide inhibitors had on average a length of 25 AAs (range: 3 to 73), a charge of +0.27 at pH 7.2 and a molecular weight of 2953 g/mol. Most common amino acids in these peptide inhibitors were leucine, glutamic acid and isoleucine (Additional file 1: Figure S8). Comparisons between the 121 peptide sequences and the consensus sequences of 16 HIV group, subtype and CRF genomes showed the highest sequence similarity with subtype B (79.8%) (Figure 5B). Aspartic acid to asparagine (25.7%) was the most common amino acid substitution between the consensus subtype B sequence and the peptide inhibitor sequences (Figure 5C).

We characterized peptide-derived regions in the subtype B genome. Of the 894 amino acid positions from which the 121 peptide inhibitors were derived, 41.2% were located in helix structures and 60.2% displayed less than 5% genetic diversity in the subtype B genome. Forty-two inhibitors had IC_50_ or EC_50_ values less than 1 μM and were derived from 249 amino acid positions in the HIV-1 genome (Additional file 2: Table S4). In the subtype B genome, these 249 positions displayed significantly lower amino acid diversity compared to the genome-wide diversity (Figure 5D, 10.1% vs 12.9%, p-value = 0.019), and were likely to be from conserved (amino acid diversity < 5%, OR: 1.43 (1.09-1.88), p-value = 0.016), solvent exposed (OR: 2.47 (1.88-3.24), p-value = 3.9E-11) and intrinsically ordered structures (disorder score < 0.4, OR: 1.75 (1.21-2.51), p-value = 0.0019) (Figure 5E).

**Figure 6:**
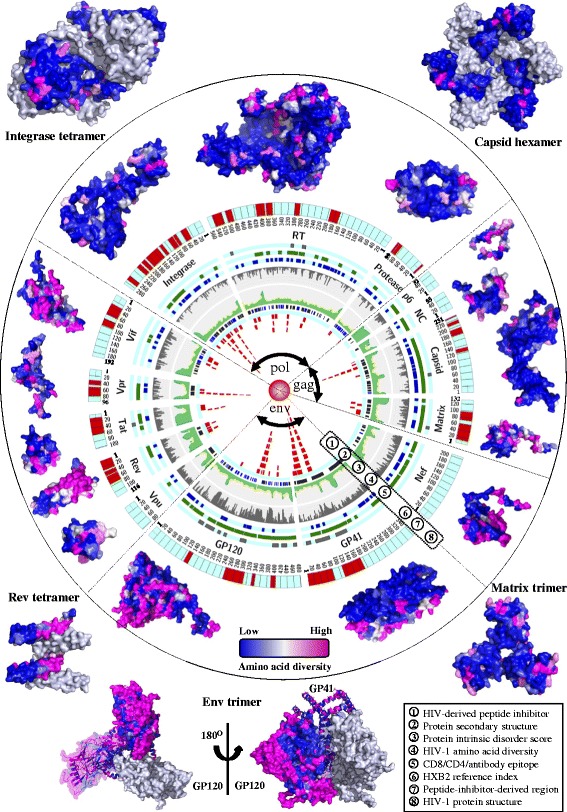
**An integrated map of HIV-1 genomic diversity and protein structures.** All 15 HIV-1 proteins are mapped in the circle with 8 layers, showing the schematic view of HIV-1 peptide inhibitors (layer 1), protein secondary structures (layer 2, dark blue: helix structures, light blue: beta-strand structures, white: random-coil structures), protein disorder scores (layer 3), amino acid diversity at individual positions (layer 4), human CD4/CD8/antibody epitope regions (layer 5, three sub-layers from inside to outside represent CD8+ T cell, CD4+ T cell and antibody epitope regions), HXB2 reference indices (layer 6), peptide-inhibitor-derived regions (layer 7) and the protein structures are colored according to the diversity of amino acid positions (layer 8, low: 0%, high: ≥30%). Three major genes (*gag*, *pol*, *env*) are annotated in the center. Structures of multimeric HIV-1 proteins are shown outside the circle and different protein units are colored separately. The list of PDB data is available in Additional file 2: Table S5. Visualization software: Circos V0.64 (http://circos.ca/).

Integrated findings from our analyses on HIV-1 genomic diversity, HIV-derived peptide inhibitors and protein structures are visualized in Figure 6. The HIV genomic sequence datasets and our toolbox developed for data visualization, genomic diversity analysis and HIV genomic alignments are freely available in Additional file 3.

## Discussion and conclusions

To our knowledge, this study provides the first large-scale analysis that investigates the genomic variability of 16 major groups, subtypes and CRFs in HIV-1 and HIV-2. While previous studies have reported the diversity of HIV genomes in small cohorts of patients (n < 250) [11-24,31], our analyses evaluated HIV genome-wide diversity using 2996 full-length genomic sequences sampled from 1705 patients worldwide. We evaluated three important aspects of HIV genomic diversity using the integrated datasets of genomic sequences, protein structures, HIV-human protein interactions, human immune epitopes and HIV-derived peptide inhibitors. Firstly, we quantified HIV genomic diversity at the individual and population levels. Secondly, we reported possible associations between HIV-1 amino acid diversity and protein multimerization, immunological constraints and HIV-human protein interactions. Thirdly, we mapped conserved regions in the HIV genome and characterized experimental and clinically used HIV-derived peptide inhibitors [7].

### Quantification of HIV genomic diversity

HIV-1 genomic diversity is the lowest within single patients and increases in the following order when different patients are considered: within subtypes, between subtypes, between groups and between HIV types (Figure 1). A nucleotide genomic diversity was quantified to be 48.3% between HIV-1 and HIV-2, 37.5% between HIV-1 groups, 14.7% between HIV-1 subtypes, 8.2% within HIV-1 subtypes, and 0.6% within single patients infected with HIV-1. These results are in a good agreement with previous studies which analyzed less than 100 sequences [13,23]. Our study quantified genomic diversity at the population level using the largest sequence dataset ever analyzed, thereby resulting in robust and accurate estimations.

As shown in Figure 2, the degree of HIV genetic diversity varied along the full-length genome. A comparison of the amino acid diversity of HIV proteins revealed the highest diversity in the envelope proteins, followed by the regulatory, accessory, structural and enzymatic proteins (Figure 4, Table 2). Our diversity analysis showed comparable results compared to our previous studies, which reported the amino acid diversity of Gag and Rev using 10862 and 4632 sequences respectively [9,32]. In contrast, estimated amino acid diversities for Pol (5.7 ± 0.9%) and Env (18.7 ± 2.7%) displayed higher values than previous reports analyzing fewer than 100 sequences of subtypes A and B [24]. Using large-scale sequence datasets, our study thus provides a complete estimation of the genetic diversity in 16 HIV proteins.

### HIV genomic diversity is shaped by multiple factors

HIV genomic diversity is driven by the high rates of viral replication, recombination and mutation [33], but other factors also play a role in shaping HIV genomic diversity. For instance, positive selection was significantly identified in the overlapping reading frames of the HIV-1 subtype B genome, probably due to functional and structural requirements [26]. To evaluate other potential factors, we correlated HIV amino acid diversity with protein multimerization, human immunological constraints and HIV-human protein interactions. We showed that CD4 T cell and antibody epitope positions in the HIV-1 genome were likely to have high amino acid diversities, supporting the hypothesis that the human immune system imposes a diversifying selective pressure on the HIV-1 genome [26]. Moreover, we found that the average amino acid diversity was significantly lower in the multimeric than in the monomeric proteins, suggesting that protein multimerization places a constraint on HIV-1 sequence conservation. While synonymous substitution rates were not significantly different, nonsynonymous substitution rates were significantly lower in multimeric proteins (Additional file 2: Table S3), suggesting that the negative selective pressure may be stronger for multimeric proteins. Previous findings on other protein families have also shown that multimeric proteins are relatively conserved and have less tolerance for amino acid substitutions, probably because of the structural and functional constraints [34-37].

We mapped 1352 HIV-human protein interactions between 15 HIV-1 proteins and 1052 human proteins. A strong association was found between the amino acid diversity of HIV-1 proteins and the number of HIV-human protein interactions (Figure 4A). HIV-1 proteins with higher genetic diversities tended to interact with more human proteins. This is probably associated with structurally disordered regions in HIV-1 proteins (Figure 4B), which provide the structural flexibility for HIV to interact with multiple human proteins [30]. For instance, GP120 uses five hypervariable loops (Additional file 1: Figure S12) to interact with various human proteins [38]. An intricate landscape of HIV-human protein complexes is made by HIV to exploit human cellular machineries during the HIV infection and production [39]. Despite the high variability of HIV, it is surprising that the nucleotide and amino acid compositions were remarkably constant across all HIV-1 and HIV-2 clades (Figure 3A, 3B), suggesting that other constraints may be active to restrict the HIV genetic diversity [25]. For instance, HIV RNA structures require the stable nucleotide compositions for the reverse transcription [25].

### Conserved drug targets in the HIV-1 genome

Many peptide inhibitors derived from HIV-1 proteins have shown promising antiviral activities and some of these inhibitors are currently under clinical trials [40,41]. Our study summarized HIV-derived peptide inhibitors published between 1993 and 2013 (Additional file 1: Figure S12-S21, Additional file 2: Table S4), and mapped the positions of these inhibitors to the HIV-1 genome (Figure 6). We showed that most peptide inhibitors were derived from the regions of HIV-1 subtype B proteins (Figure 5B), which had conserved, solvent exposed and intrinsically ordered structures (Figure 5E). This information enhances current understanding of HIV-derived peptide inhibitors, which may provide valuable guidelines for the design of novel peptide inhibitors [42,43].

In the full-length genome, we identified conserved regions in Capsid, Nucleocapsid, Protease, RT, Integrase, Vpr and N-terminal domain of GP41 (Figure 2). These conserved regions have been targeted by known anti-HIV inhibitors (Figure 6). For instance, over 40 experimental inhibitors with promising antiviral activities have been designed to target Capsid and Nucleocapsid [9]. HIV enzymes (Protease, RT, Integrase) are targeted by the majority of the FDA-approved antiretroviral drugs. Peptide inhibitor T20 targets the N-terminal heptad domain of GP41 [44]. Overall, our sequence analysis mapped the conserved drug target regions in the HIV-1 genome, providing useful information for drug design.

### Implications for HIV vaccine development

HIV subtype- and geography-specific vaccination has been proposed to contend with the challenges imposed by the high HIV genetic diversity [2]. Previous vaccine trials were carried out in regional populations dominated by a single HIV-1 subtype or CRF. For instance, the STEP [11] and RV144 [12] vaccine trials targeted patient populations mainly infected by subtype B and CRF01_AE, respectively. Particularly, the RV144 trial in 2009 showed the first sign that a prime–boost strategy achieved a modest vaccine efficacy (31.2%) in the heterosexual population, which was at risk for infections with CRF01_AE [12,31]. In our analysis, CRF01_AE has the lowest genomic diversity among the 12 analyzed HIV groups, subtypes and CRFs (Figures 1D, 2A, 2B). It is thus tempting to speculate that the low diversity of CRF01_AE may have contributed to the success of the RV144 trial.

As conserved epitopes are ideal targets for potential vaccines to contend with the high HIV diversity [24,45], our study highlighted position-specific conservation along the full-length HIV genome (Figure 6). Moreover, HIV-1 consensus sequences have been considered as potential vaccine candidates to minimize genetic diversity between vaccine candidates and circulating strains [2]. Previous analyses on fewer than 100 Matrix and GP160 sequences reported that genetic diversity between subtype-specific consensus sequences and circulating strains was only half of the genetic diversity between circulating strains from the same subtype [2]. We found that in the full-length HIV genome, this effect was much smaller as we only observed a 32.5% reduction of the genomic diversity (8.3% vs. 12.3%, Additional file 1: Figure S24). As the most explored vaccine target protein, GP120 has the highest genetic diversity among all HIV proteins (Table 2), presenting a challenge in the search for broadly neutralizing antibodies and vaccines [46]. Furthermore, we mapped the global distribution of HIV-1 genomic diversity (Additional file 1: Figure S4). Our results showed the highest HIV genomic diversity in Central Africa, the birthplace of HIV [1,29], which suggested the difficulty of implementing HIV vaccines in this region.

### Limitations and future perspectives

The limited number of full-length genomic sequences for HIV-1 subtypes H, J and K, group P and HIV-2 group B (n < 10) may have affected our estimation of sequence diversity, but consistent patterns were detected in the full-length genome across different HIV groups and subtypes (Figure 2). Sequence diversity investigations at a population level for such clades may improve when adding more sequences from individual proteins. However, it would also result in difficulties (e.g. lack of patient or treatment information) that may affect our confidence in data quality, and consequently the accuracy of our results. Our structural analysis focuses on HIV-1 proteins because most PDB data are available for HIV-1 but not for HIV-2 (Additional file 2: Table S5). Beside the multiple factors described in our study, other driving forces may shape HIV genetic diversity [25,47] and the genetic diversity data reported in our study can be useful for further investigations. Information on positions involved in HIV-human protein interactions is largely lacking, restricting our analysis from exploring the genetic diversity of protein interaction positions. Despite an extensive search, anti-HIV peptide inhibitors other than the ones described here may have been developed, but major changes in our conclusions regarding the known peptide-derived regions are not expected. Future studies are still needed to clarify how to improve vaccines and anti-HIV inhibitors based on the information of HIV genomic diversity. The increased knowledge of genome-wide diversity from our study may contribute to a better rational design of HIV vaccines and inhibitors.

## Methods

### HIV genomic sequence dataset

In August 2013, we retrieved 3607 nucleotide genomic sequences of major HIV-1 and HIV-2 clades (HIV-2 group A and B, HIV-1 group N, O, P, subtype A1, B, C, D, F1, G, H, J, K, CRF01_AE, CRF02_AG) from the HIV Los Alamos database (www.hiv.lanl.gov/). HIV-1 subtype was determined by the Rega [48] and COMET [49] subtyping tools. The quality criteria for removing duplicates and sequences with hypermutations, stop codons, ambiguous nucleotides or discordant subtype classification were described in [9]. The sequence dataset that fulfilled the quality criteria comprised 2996 genomic sequences, sampled from 1684 HIV-1 and 21 HIV-2 patients between 1982 and 2013. Information on genomic sequence datasets is summarized in Table 1.

Nucleotide genomic sequences were aligned using MUSCLE [50]. Protein regions encoded by their respective open reading frames (ORFs) were concatenated according to the reference strains (HIV-1: HXB2, HIV-2: BEN). For each HIV protein coding region, the translation of nucleotide to amino acid sequence alignments was optimized by our nucleotide to amino acid alignment toolbox. This toolbox maximizes amino acid matches, including in overlapping reading frames, based on the BLOSUM62 substitution matrix. Sequence alignments were further curated using Seaview v4.3 [51]. To show the alignment quality, we measured the percentages of deletions and insertions in the multiple sequence alignments (MSAs) of HIV genomes, which were less than 1.31% in 16 individual HIV clades (Additional file 2: Table S6). Our alignment toolbox and genomic sequences are available in Additional file 3.

### PDB, HIV-human protein interaction, CD4/CD8/antibody epitope datasets

As of February 2014, we queried HIV PDB data from the RCSB Protein Data Bank using sequence search; PDB quality was then examined using PDBREPORT [52] (Additional file 2: Table S5). We extracted HIV-human protein interactions (interaction type: physical interaction) from the NCBI HIV-1 human protein interaction database [53]. From the HIV Los Alamos database (http://www.hiv.lanl.gov/content/immunology/), we extracted the human CD4 T cell and antibody epitopes in HIV-1. For human CTL/CD8 T cell epitopes, we included the best-defined CTL epitopes of the A-list described in [54] (Additional file 2: Table S7).

### HIV-derived peptide inhibitor dataset

HIV-derived peptide inhibitors have their amino acid sequences derived from HIV proteins. We searched for English articles in PubMed published between January 1983 and September 2013 using the keywords “HIV peptide inhibitor”, “HIV [protein name] peptide” and “HIV [protein name] inhibitor”. References from primary studies, review articles and peptide design papers were also reviewed. If more than one peptide inhibitor were reported in one publication, only the most promising peptide inhibitors as indicated by the abstract of articles were collected. If data on the same inhibitors was reported by more than one publication, only the latest results were retained. Additional file 2: Table S4 summarizes the 121 peptide inhibitors with corresponding information on peptide sequences, peptide-derived regions, target proteins, inhibitory activities and references.

### Protein secondary structure

For HIV-1 proteins (Rev, GP41) whose crystalized structures are not fully resolved in the PDB data, we used the sequence-based method PSIPRED V3.0 [55] to estimate protein secondary structures. For the other HIV-1 proteins with available PDB data (Additional file 2: Table S5), we assessed protein secondary structures using both PSIPRED V3.0 [55] and 2Struc [56]. 2Struc is a software platform which integrates 8 PDB-based methods: DSSP_CONT, DSSP, KAKSI, PALSSE, P-SEA, STICKS, STRIDE and XTLSSTR [56]. Alpha-helix, beta-strand and random-coil structures were estimated using the majority voting of above 9 methods. Prediction similarities between these 9 methods are shown in Additional file 1: Figure S24.

### Protein intrinsic disorder

Protein disordered regions are exploited by the virus to invade cellular host systems [30]; these regions are often structurally unstable without their partner molecules [57]. We estimated the intrinsically disordered structures of HIV-1 subtype B proteins using three software packages: MetaPrDOS [57], VSL2P [58] and PreDisorder v1.1 [59]. A disorder score (a numerical value between 0 and 1) of each amino acid position was estimated by 17 methods in these 3 software packages. An amino acid position was estimated as intrinsically disordered if its disorder score was above the cutoff value of 0.5 [57-59]. The intrinsically disordered positions were identified based on the majority voting of 17 methods. Prediction similarities between these 17 methods are shown in Additional file 1: Figure S13.

### Solvent accessible surface area

We estimated protein solvent accessible surface areas (ASA) using Chimera V1.6.1 [60] (default parameters). Provided with PDB data in Additional file 2: Table S5, we calculated the ASA at each amino acid of all HIV-1 protein units. For each of the 20 amino acids, a distribution of its ASA scores over 15 HIV-1 proteins was obtained and the maximum ASA was identified therein. An amino acid at a specific position was considered buried if its ASA was lower than 25% of the maximum ASA for the corresponding amino acid [61] (Additional file 2: Table S8).

### Phylogenetic analysis

Our phylogenetic analysis was performed using 1384 nucleotide genomic sequences of 14 HIV groups and pure subtypes (thus excluding CRFs), obtained from the earliest sampling time (one sequence per patient). To prepare the alignment, we also removed ambiguous regions containing multiple insertions, deletions and hypervariable positions (HXB2 index: 1126-1182, 6866-7003, 7106-7154, 7773-7842, 7981-8032, 8897-9383). Maximum-likelihood phylogenetic trees were obtained using the multi-threaded FastTree V2.1 [62]. Our software parameters were set to 100 bootstrap replicates, the fully optimized GTR (generalized time-reversible) model, the continuous gamma distribution and the exhaustive nearest-neighbor interchange approach. The consensus phylogenetic tree with bootstrap supports was obtained using the seqboot tool in Phylip V3.69 (http://evolution.genetics.washington.edu/phylip.html).

### Ratio of non-synonymous and synonymous rate (dN/dS)

Following the protocol described in [26], we performed the dN/dS analysis for HIV-1 subtype B using 495 genomic sequences sampled from different treatment-naïve patients. We firstly constructed a maximum-likelihood phylogenetic tree using FastTree V2.1 [62] (parameters: continuous gamma distribution, generalized time-reversible (GTR) model). Provided with the constructed phylogenetic tree and the codon sequence dataset, we then applied HyPhy V2.1.0 [63] to estimate the non-synonymous (dN) and synonymous (dS) substitution rates. We employed the single likelihood ancestor counting (SLAC) model with the optimized GTR [63]. Ambiguous nucleotides were resolved by averaging over all possible states for the ancestral sequence reconstruction [63]. Statistical significance of dN/dS was measured by the continuous extension of binomial distributions [63]. The above procedure was also performed for HIV-1 subtype A1, C and CRF01_AE genomes using our sequence datasets (Additional file 2: Table S2).

### Quantification of sequence diversity

Sequence diversity was calculated based on the pairwise nucleotide (NT) and amino acid (AA) comparisons [9,64]. When calculating the amino acid diversity of HIV genome, we concatenated the amino acid sequences of 15 HIV protein coding regions in the full-length genome. Suppose the sequence dataset D contains L sequences with N positions, genetic diversity at position n is calculated by: $GD\left({D}_n\right)=1-\frac{2}{L\left(L-1\right)}{\displaystyle \sum_{i=1}^L{\displaystyle \sum_{j=i+1}^L\delta \left({D}_{n,i}={D}_{n,j}\right)}}$, where *D*_*n,i*_ is the NT or AA form of the position *n* at the i^th^ sequence in the dataset D, *δ* represents the Kronecker symbol, *δ*(*D*_*n,i*_ = *D*_*n,j*_) equals 1 if *D*_*n,i*_ is identical to *D*_*n,j*_; otherwise 0. Given the sequence dataset D, intra-clade genetic diversity *AGD(D)* is defined as the average genetic diversity of all positions: $AGD(D)=1-\frac{1}{N}{\displaystyle \sum_{n=1}^N\frac{2}{L\left(L-1\right)}{\displaystyle \sum_{i=1}^L{\displaystyle \sum_{j=i+1}^L\delta \left({D}_{n,i}={D}_{n,j}\right).}}}$ Suppose two sequence datasets D1 and D2 aligned with the same reference genome have the number of sequences *L*_1_ and *L*_2_ respectively. The inter-clade genetic diversity between D1 and D2 is defined as: $RGD\left(D1,D2\right)=1-\frac{1}{N}{\displaystyle \sum_{n=1}^N\frac{1}{L_1\times {L}_2}{\displaystyle \sum_{i=1}^{L_1}{\displaystyle \sum_{j=1}^{L_2}\delta \left(D{1}_{n,i}=D{2}_{n,j}\right)}}}$.

Furthermore, only positions for which less than 20% of sequences had gaps were considered and gaps were treated as missing data. Intra- and inter-clade genetic diversity was measured using one genomic sequence per patient, while intra-patient diversity was calculated using more than one genomic sequence sampled from individual patients. The Mann–Whitney *U* test was performed to compare the distributions of genetic diversity and a significant difference was identified if a p-value was lower than 0.05 [65]. Our Matlab implementation of genomic diversity analysis is available in Additional file 3.

## Additional files

Additional file 1:
**Figures and tables.** **Figure S1.** Gene maps and protein structures of HIV-1 and HIV-2. **Figure S2.** Distribution plots of nucleotide and AA diversity among HIV types, groups and subtypes. **Figure S3.** Distribution plots of AA diversity between HIV-1 subtype B/C and the other HIV groups**/**subtypes. **Figure S4.** Global distribution of HIV-1 genomic diversity. **Figure S5.** AA diversity along the full-length HIV genome. **Figure S6.** Global distribution of HIV-1 genomic diversity. **Figure S7.** Average AA diversity of HIV-1 protein clusters and number of HIV-human protein interactions. **Figure S8.** AA composition of HIV-1 subtype B genome, HIV-1 peptide-derived regions and sequences of HIV-derived peptide inhibitors. **Figure S9.** Average AA diversity of peptide-derived regions in HIV-1 subtype B. **Figure S10.** Solvent accessible surface area of peptide-derived regions in the HIV-1 subtype B genome. **Figure S11.** Protein intrinsic disorder scores of peptide-derived regions in the HIV-1 subtype B genome. **Figure S12.** Protein structure of the HIV-1 GP120-CD4-Fab 48d complex (PDB: 2B4C, 3U4E) and mapped GP120 peptide-derived inhibitors. **Figure S13.** GP41 structure and GP41-derived peptide inhibitors. **Figure S14.** HIV-1 Integrase tetramer and Integrase-derived peptide inhibitors. **Figure S15.** HIV-1 RT structure and RT-derived peptide inhibitors. **Figure S16.** HIV-1 Protease homodimer structure and protease-derived peptide inhibitors. **Figure S17.** HIV-1 Tat structure and Tat-derived peptide inhibitors. **Figure S18.** HIV-1 Vpr structure and Vpr-derived peptides. **Figure S19.** HIV-1 Rev tetramer structure and Rev-derived peptide inhibitors. **Figure S20.** Structure of HIV-1 Capsid monomer and Capsid-derived peptide inhibitors. **Figure S21.** HIV-1 Vif structure and Vif-derived peptide inhibitors. **Figure S22.** Distribution plots of AA diversity between the consensus and the circulating genomes, within circulating genomes. **Figure S23.** Prediction similarities of the consensus and the 9 protein secondary structure prediction methods. **Figure S24.** Prediction similarities of the consensus and 17 methods for protein intrinsically disorder prediction. Additional file 2: Table S1. Average amino acid diversity of HIV monomeric and multimeric proteins. **Table S2.** Summary of average AA diversity, average dN, average dS and average dN/dS in the HIV-1 subtype A1, B, C and CRF 01_AE genomes. **Table S3.** Statistical of dN/dS, dN and dS distributions in the monomeric and multimeric protein groups of the HIV-1 subtype A1, B, C and CRF01_AE genomes. **Table S4.** Summary of 121 peptide inhibitors derived from HIV-1 proteins. **Table S5.** Summary of protein structures and PDB data for HIV-1 and HIV-2 proteins. **Table S6.** Percentages of deletions and insertions in the HIV-1 and HIV-2 full-length genomic sequence alignments. **Table S7.** Summary of antibody, CD4+, CD8+ T cell epitope positions. **Table S8.** Cutoffs for determining solvent exposed residues. Additional file 3:
**Software.** Our Matlab toolbox developed for data visualization, genomic diversity analysis and HIV genomic alignment. Our sequence datasets are also included.

## Abbreviations

AA: Amino acid
ASA: Solvent accessible surface area
CA: Capsid
CRF: Circulating recombinant form
dN/dS: Ratio of non-synonymous to synonymous
EC5_0_: 50% effective concentration
GTR: Generalized time reversible model
LTR: Long terminal region
IC_50_: 50% inhibitory concentration
IN: Integrase
MA: Matrix
MSA: Multiple sequence alignment
NC: Nucleocapsid
NT: Nucleotide
OR: Odds ratio
ORF: Open reading frame
PDB: Protein data bank
PR: Protease
RT: Reverse transcriptase

## References

[1] Hemelaar J (2012). The origin and diversity of the HIV-1 pandemic. Trends Mol Med.

[2] Gaschen B, Taylor J, Yusim K, Foley B, Gao F, Lang D (2002). Diversity considerations in HIV-1 vaccine selection. Science.

[3] Frankel AD, Young JA (1998). HIV-1: fifteen proteins and an RNA. Annu Rev Biochem.

[4] Engelman A, Cherepanov P (2012). The structural biology of HIV-1: mechanistic and therapeutic insights. Nat Rev Microbiol.

[5] Acosta EG, Kumar A, Bartenschlager R (2014). Revisiting dengue virus-host cell interaction: new insights into molecular and cellular virology. Adv Virus Res.

[6] Perry CM (2014). Elvitegravir/cobicistat/emtricitabine/tenofovir disoproxil fumarate single-tablet regimen (Stribild(R)): a review of its use in the management of HIV-1 infection in adults. Drugs.

[7] Tilton JC, Doms RW (2010). Entry inhibitors in the treatment of HIV-1 infection. Antiviral Res.

[8] Fauci AS, Folkers GK, Dieffenbach CW (2013). HIV-AIDS: much accomplished, much to do. Nat Immunol.

[9] Li G, Verheyen J, Rhee SY, Voet A, Vandamme AM, Theys K (2013). Functional conservation of HIV-1 gag: implications for rational drug design. Retrovirology.

[10] Stephenson KE, Barouch DH (2013). A global approach to HIV-1 vaccine development. Immunol Rev.

[11] Rolland M, Tovanabutra S, deCamp AC, Frahm N, Gilbert PB, Sanders-Buell E (2011). Genetic impact of vaccination on breakthrough HIV-1 sequences from the STEP trial. Nat Med.

[12] Rolland M, Edlefsen PT, Larsen BB, Tovanabutra S, Sanders-Buell E, Hertz T (2012). Increased HIV-1 vaccine efficacy against viruses with genetic signatures in Env V2. Nature.

[13] Arien KK, Vanham G, Arts EJ (2007). Is HIV-1 evolving to a less virulent form in humans?. Nat Rev Microbiol.

[14] Herbeck JT, Rolland M, Liu Y, McLaughlin S, McNevin J, Zhao H (2011). Demographic processes affect HIV-1 evolution in primary infection before the onset of selective processes. J Virol.

[15] Rousseau CM, Birditt BA, McKay AR, Stoddard JN, Lee TC, McLaughlin S (2006). Large-scale amplification, cloning and sequencing of near full-length HIV-1 subtype C genomes. J Virol Methods.

[16] Wang YE, Li B, Carlson JM, Streeck H, Gladden AD, Goodman R (2009). Protective HLA class I alleles that restrict acute-phase CD8+ T-cell responses are associated with viral escape mutations located in highly conserved regions of human immunodeficiency virus type 1. J Virol.

[17] Brown BK, Darden JM, Tovanabutra S, Oblander T, Frost J, Sanders-Buell E (2005). Biologic and genetic characterization of a panel of 60 human immunodeficiency virus type 1 isolates, representing clades A, B, C, D, CRF01_AE, and CRF02_AG, for the development and assessment of candidate vaccines. J Virol.

[18] Fernandez-Garcia A, Cuevas MT, Munoz-Nieto M, Ocampo A, Pinilla M, Garcia V (2009). Development of a panel of well-characterized human immunodeficiency virus type 1 isolates from newly diagnosed patients including acute and recent infections. AIDS Res Hum Retroviruses.

[19] Kousiappa I, Van De Vijver DA, Kostrikis LG (2009). Near full-length genetic analysis of HIV sequences derived from Cyprus: evidence of a highly polyphyletic and evolving infection. AIDS Res Hum Retroviruses.

[20] Sanabani SS, Pessoa R, Soares de Oliveira AC, Martinez VP, Giret MT, de Menezes Succi RC (2013). Variability of HIV-1 genomes among children and adolescents from Sao Paulo, Brazil. PLoS One.

[21] Fernandez-Garcia A, Revilla A, Vazquez-de Parga E, Vinogradova A, Rakhmanova A, Karamov E (2012). The analysis of near full-length genome sequences of HIV type 1 subtype A viruses from Russia supports the monophyly of major intrasubtype clusters. AIDS Res Hum Retroviruses.

[22] Henn MR, Boutwell CL, Charlebois P, Lennon NJ, Power KA, Macalalad AR (2012). Whole genome deep sequencing of HIV-1 reveals the impact of early minor variants upon immune recognition during acute infection. PLoS Pathog.

[23] Guyader M, Emerman M, Sonigo P, Clavel F, Montagnier L, Alizon M (1987). Genome organization and transactivation of the human immunodeficiency virus type 2. Nature.

[24] Korber B, Gaschen B, Yusim K, Thakallapally R, Kesmir C, Detours V (2001). Evolutionary and immunological implications of contemporary HIV-1 variation. Br Med Bull.

[25] van der Kuyl AC, Berkhout B (2012). The biased nucleotide composition of the HIV genome: a constant factor in a highly variable virus. Retrovirology.

[26] Snoeck J, Fellay J, Bartha I, Douek DC, Telenti A (2011). Mapping of positive selection sites in the HIV-1 genome in the context of RNA and protein structural constraints. Retrovirology.

[27] Mayrose I, Stern A, Burdelova EO, Sabo Y, Laham-Karam N, Zamostiano R (2013). Synonymous site conservation in the HIV-1 genome. BMC Evol Biol.

[28] Santoro MM, Perno CF (2013). HIV-1 Genetic Variability and Clinical Implications. ISRN Microbiol.

[29] Hemelaar J, Gouws E, Ghys PD, Osmanov S (2011). Isolation W-UNfH, Characterisation: Global trends in molecular epidemiology of HIV-1 during 2000-2007. AIDS.

[30] Xue B, Mizianty MJ, Kurgan L, Uversky VN (2012). Protein intrinsic disorder as a flexible armor and a weapon of HIV-1. Cell Mol Life Sci.

[31] Haynes BF, Gilbert PB, McElrath MJ, Zolla-Pazner S, Tomaras GD, Alam SM, et al. Immune-correlates analysis of an HIV-1 vaccine efficacy trial. Engl J Med. 2012;366:1275–86. 10.1056/NEJMoa1113425 PMC3371689 22475592

[32] Boons E, Li G, Vanstreels E, Vercruysse T, Pannecouque C, Vandamme A-M (2014). A stably expressed llama single-domain intrabody targeting Rev displays broad-spectrum anti-HIV activity. Antiviral Research.

[33] Rambaut A, Posada D, Crandall KA, Holmes EC (2004). The causes and consequences of HIV evolution. Nat Rev Genet.

[34] Falkowski PG, Fenchel T, Delong EF (2008). The microbial engines that drive Earth's biogeochemical cycles. Science.

[35] Dunwell JM, Culham A, Carter CE, Sosa-Aguirre CR, Goodenough PW (2001). Evolution of functional diversity in the cupin superfamily. Trends Biochem Sci.

[36] Buck PM, Kumar S, Singh SK (2013). On the role of aggregation prone regions in protein evolution, stability, and enzymatic catalysis: insights from diverse analyses. PLoS Comput Biol.

[37] Li G, Theys K, Verheyen J, Pineda-Pena A, Khouri R, Piampongsant S (2015). A new ensemble coevolution system for detecting HIV-1 protein coevolution. Biol Direct.

[38] Zolla-Pazner S, Cardozo T (2010). Structure-function relationships of HIV-1 envelope sequence-variable regions refocus vaccine design. Nat Rev Immunol.

[39] Jager S, Cimermancic P, Gulbahce N, Johnson JR, McGovern KE, Clarke SC (2012). Global landscape of HIV-human protein complexes. Nature.

[40] Burnett JC, Zaia JA, Rossi JJ (2012). Creating genetic resistance to HIV. Curr Opin Immunol.

[41] He Y (2013). Synthesized peptide inhibitors of HIV-1 gp41-dependent membrane fusion. Curr Pharm Des.

[42] Roberts KE, Cushing PR, Boisguerin P, Madden DR, Donald BR (2012). Computational design of a PDZ domain peptide inhibitor that rescues CFTR activity. PLoS Comput Biol.

[43] Durrant JD, McCammon JA (2011). Molecular dynamics simulations and drug discovery. BMC Biol.

[44] Liu S, Lu H, Niu J, Xu Y, Wu S, Jiang S (2005). Different from the HIV fusion inhibitor C34, the anti-HIV drug Fuzeon (T-20) inhibits HIV-1 entry by targeting multiple sites in gp41 and gp120. J Biol Chem.

[45] Rolland M, Nickle DC, Mullins JI (2007). HIV-1 group M conserved elements vaccine. PLoS Pathog.

[46] Kwong PD, Mascola JR, Nabel GJ (2013). Broadly neutralizing antibodies and the search for an HIV-1 vaccine: the end of the beginning. Nat Rev Immunol.

[47] Li G, Verheyen J, Theys K, Piampongsant S, Van Laethem K, Vandamme AM (2014). HIV-1 Gag C-terminal amino acid substitutions emerging under selective pressure of protease inhibitors in patient populations infected with different HIV-1 subtypes. Retrovirology.

[48] Pineda-Pena AC, Faria NR, Imbrechts S, Libin P, Abecasis AB, Deforche K (2013). Automated subtyping of HIV-1 genetic sequences for clinical and surveillance purposes: Performance evaluation of the new REGA version 3 and seven other tools. Infect Genet Evol.

[49] Struck D, Lawyer G, Ternes AM, Schmit JC, Bercoff DP (2015). COMET: adaptive context-based modeling for ultrafast HIV-1 subtype identification. Nucleic Acids Res.

[50] Edgar RC (2004). MUSCLE: multiple sequence alignment with high accuracy and high throughput. Nucleic Acids Res.

[51] Gouy M, Guindon S, Gascuel O (2010). SeaView version 4: A multiplatform graphical user interface for sequence alignment and phylogenetic tree building. Mol Biol Evol.

[52] Hooft RW, Vriend G, Sander C, Abola EE (1996). Errors in protein structures. Nature.

[53] Pinney JW, Dickerson JE, Fu W, Sanders-Beer BE, Ptak RG, Robertson DL (2009). HIV-host interactions: a map of viral perturbation of the host system. AIDS.

[54] Llano A, Frahm N, Brander C (2009). How to optimally define optimal cytotoxic T lymphocyte epitopes in HIV infection. HIV Mol Immunol.

[55] Buchan DW, Ward SM, Lobley AE, Nugent TC, Bryson K, Jones DT (2010). Protein annotation and modelling servers at University College London. Nucleic Acids Res.

[56] Klose DP, Wallace BA, Janes RW (2010). 2Struc: the secondary structure server. Bioinformatics.

[57] Ishida T, Kinoshita K (2008). Prediction of disordered regions in proteins based on the meta approach. Bioinformatics.

[58] Peng K, Radivojac P, Vucetic S, Dunker AK, Obradovic Z (2006). Length-dependent prediction of protein intrinsic disorder. BMC Bioinformatics.

[59] Deng X, Eickholt J, Cheng J (2009). PreDisorder: ab initio sequence-based prediction of protein disordered regions. BMC Bioinformatics.

[60] Pettersen EF, Goddard TD, Huang CC, Couch GS, Greenblatt DM, Meng EC (2004). UCSF Chimera–a visualization system for exploratory research and analysis. J Comput Chem.

[61] Levy ED (2010). A simple definition of structural regions in proteins and its use in analyzing interface evolution. J Mol Biol.

[62] Price MN, Dehal PS, Arkin AP (2010). FastTree 2–approximately maximum-likelihood trees for large alignments. PLoS One.

[63] Pond SL, Frost SD, Muse SV (2005). HyPhy: hypothesis testing using phylogenies. Bioinformatics.

[64] Spira S, Wainberg MA, Loemba H, Turner D, Brenner BG (2003). Impact of clade diversity on HIV-1 virulence, antiretroviral drug sensitivity and drug resistance. J Antimicrob Chemother.

[65] Li G: HIV genome-wide diversity, interaction and coevolution. Doctoral thesis. University of Leuven, Faculty of medicine; 2014. (https://lirias.kuleuven.be/handle/123456789/460408)

