# Supplementary material for: An integrated map of HIV genome-wide variation from a population perspective
Source: Retrovirology. 2015 Feb 15;12:18. doi: 10.1186/s12977-015-0148-6 (PMC4358901; doi:10.1186/s12977-015-0148-6)
Supplement: Additional file 1: — Figures and tables. Figure S1. Gene maps and protein structures of HIV-1 and HIV-2. Figure S2. Distribution plots of nucleotide and AA diversity among HIV types, groups and subtypes. Figure S3. Distribution plots of AA diversity between HIV-1 subtype B/C and the other HIV groups/subtypes. Figure S4. Global distribution of HIV-1 genomic diversity. Figure S5. AA diversity along the full-length HIV genome. Figure S6. Global distribution of HIV-1 genomic diversity. Figure S7. Average AA diversity of HIV-1 protein clusters and number of HIV-human protein interactions. Figure S8. AA composition of HIV-1 subtype B genome, HIV-1 peptide-derived regions and sequences of HIV-derived peptide inhibitors. Figure S9. Average AA diversity of peptide-derived regions in HIV-1 subtype B. Figure S10. Solvent accessible surface area of peptide-derived regions in the HIV-1 subtype B genome. Figure S11. Protein intrinsic disorder scores of peptide-derived regions in the HIV-1 subtype B genome. Figure S12. Protein structure of the HIV-1 GP120-CD4-Fab 48d complex (PDB: 2B4C, 3U4E) and mapped GP120 peptide-derived inhibitors. Figure S13. GP41 structure and GP41-derived peptide inhibitors. Figure S14. HIV-1 Integrase tetramer and Integrase-derived peptide inhibitors. Figure S15. HIV-1 RT structure and RT-derived peptide inhibitors. Figure S16. HIV-1 Protease homodimer structure and protease-derived peptide inhibitors. Figure S17. HIV-1 Tat structure and Tat-derived peptide inhibitors. Figure S18. HIV-1 Vpr structure and Vpr-derived peptides. Figure S19. HIV-1 Rev tetramer structure and Rev-derived peptide inhibitors. Figure S20. Structure of HIV-1 Capsid monomer and Capsid-derived peptide inhibitors. Figure S21. HIV-1 Vif structure and Vif-derived peptide inhibitors. Figure S22. Distribution plots of AA diversity between the consensus and the circulating genomes, within circulating genomes. Figure S23. Prediction similarities of the consensus and the 9 protein secondary structure prediction [file 12977_2015_148_MOESM1_ESM.pdf]

Additional figures

An integrated map of HIV genome-wide variation from a population perspective

Authors and Affiliations

Guangdi Li[1,2*], Supinya Piampongsant[2], Nuno Rodrigues Faria[3], Arnout Voet[4], Andrea-Clemencia Pineda-Peña[2,5], Ricardo Khouri[2,6], Philippe Lemey[2], Anne-Mieke Vandamme[2,7], Kristof Theys [2*]

[1] Metabolic Syndrome Research Center, the Second Xiangya Hospital, Central South University, Changsha, Hunan, China

[2] Rega Institute for Medical Research, Department of Microbiology and Immunology, KU Leuven, Leuven, Belgium

[3] Department of Zoology, University of Oxford, Oxford OX1-3PS, UK

[4] Zhang IRU, RIKEN Institute Laboratories, Hirosawa 2-1, Wako-shi, Saitama, Japan

[5] Clinical and Molecular Infectious Disease Group, Faculty of Sciences and Mathematics, Universidad del Rosario, Bogotá, Colombia

[6] LIMI-LIP, Centro de Pesquisa Gonçalo Moniz, FIOCRUZ, Salvador-Bahia, Brasil

[7] Centro de Malária e Outras Doenças Tropicais and Unidade de Microbiologia, Instituto de Higiene e Medicina Tropical, Universidade Nova de Lisboa, Lisbon, Portugal

Abbreviations:

AA: amino acid, ASA: solvent accessible surface area, CA: capsid, CTD: C-terminal domain, gag: group-specific antigen gene, dN/dS: ratio of non-synonymous to synonymous, NTD: N-terminal domain, K_d: dissociation constant, MA: matrix, NC: nucleocapsid, PR: protease, RT: reverse transcriptase, IN: integrase, LTR: long terminal region, ORF: open reading frame, Vif: viral infectivity factor, Vpr: viral protein R, Tat: trans-activator of transcription, Vpu: viral protein U, Rev: regulator of virion expression, sp: signal peptide, GP120: surface glycoprotein GP120, GP41: transmembrane glycoprotein GP41, Nef: negative regulatory factor, env: envelope gene, p2: spacer peptide 2, p1: spacer peptide 1, FP: fusion peptide, NHR: N-terminal heptad repeat, CHR: C-terminal heptad repeat, MPER: membrane-proximal external region, TM: transmembrane domain.

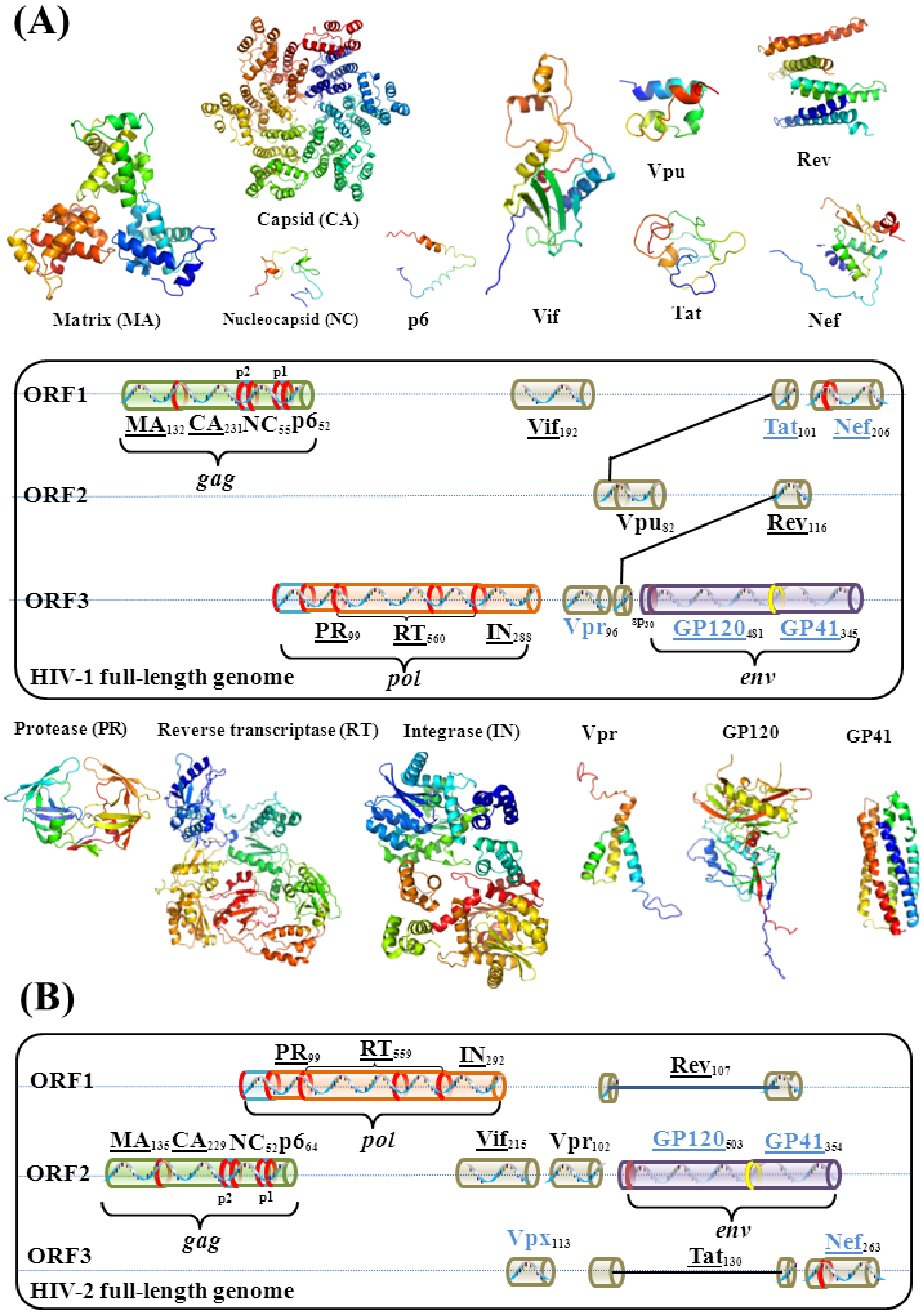

Figure S 1: (A) Coding regions of HIV-1 full-length genome with gene maps, protein names and protein structures. (B) Coding regions of HIV-2 full-length genome with gene

maps and protein names. The gene map is annotated with protein names and amino acids lengths (HIV-1 reference: HXB2, HIV-2 reference: BEN). Multimeric proteins are underlined and extracellular proteins have their names colored blue. HIV-1 protease cleavage sites are denoted by red rings. Human protease (furin, PC1) cleavage site [1] is indicated by a yellow ring in the Env protein. Protein regions indicated in the gene map are not to scale. The PDB list of HIV-1 proteins is available in Table S5.

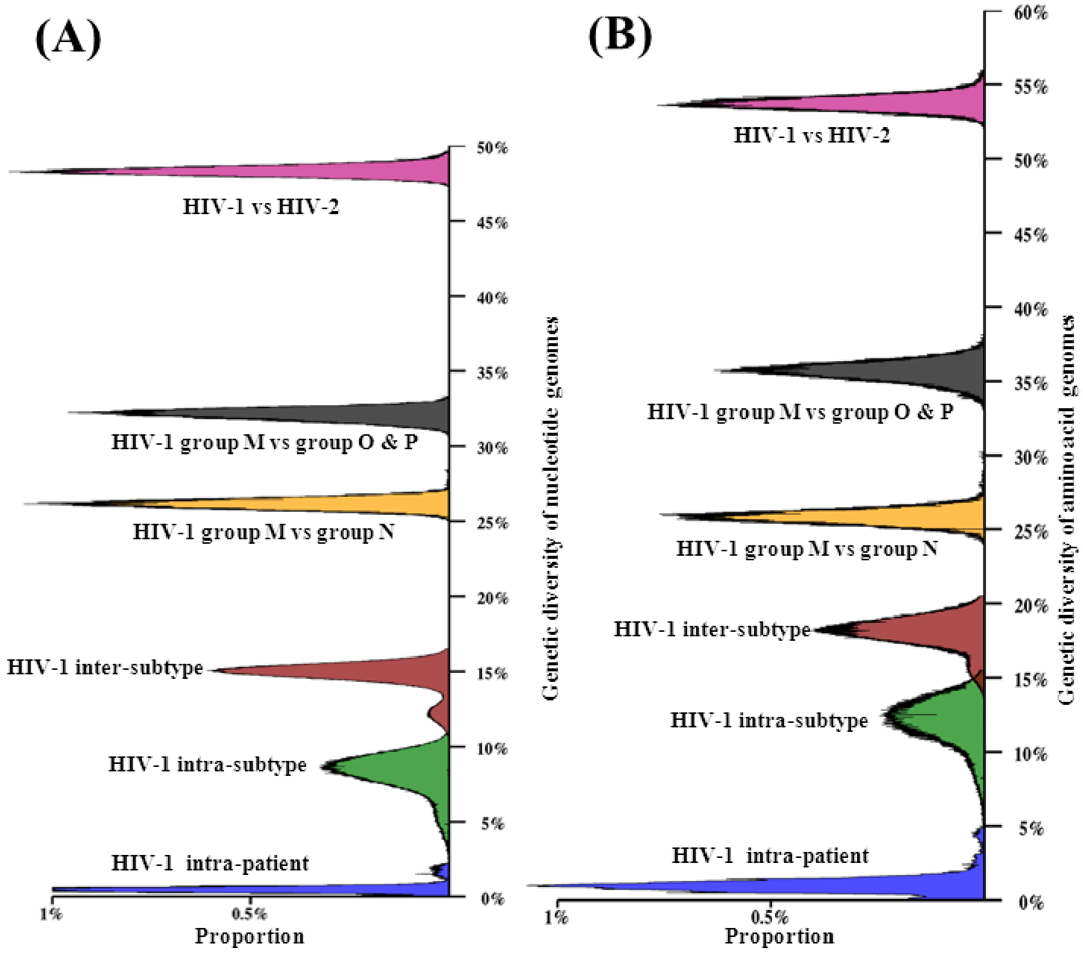

Figure S 2: Distribution plots of nucleotide (A) and amino acid (B) diversity among HIV types, groups and subtypes. See figure captions in Figure 1.

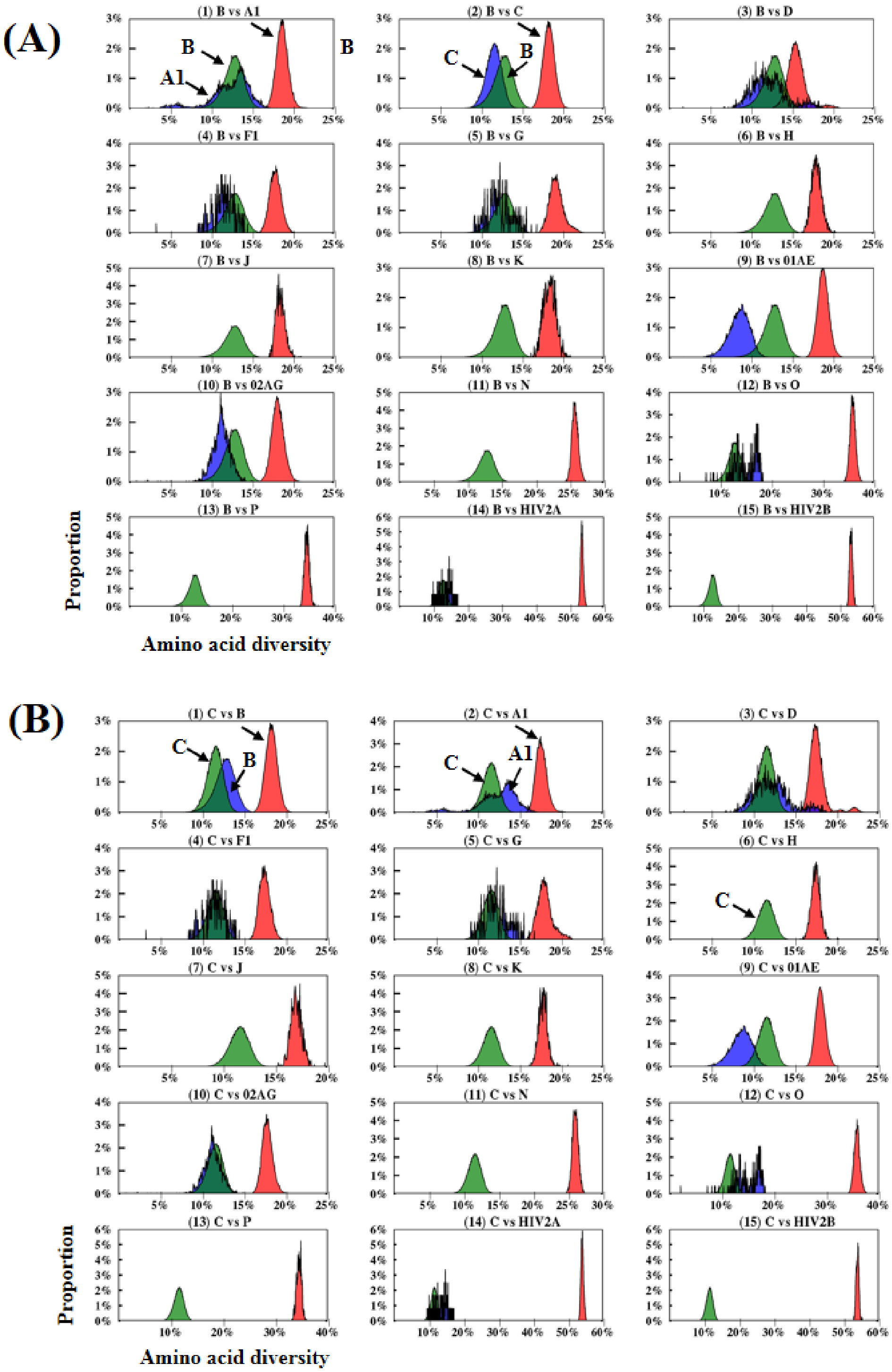

Figure S 3: (A) Distribution plots of amino acid diversity between HIV-1 subtype B and the other clades (HIV-1 subtypes, HIV-1 and HIV-2 groups) in the protein coding regions of the full-length genome. Each graph displays the distribution plots of diversity within subtype B (green), diversity within the other subtype/group (blue) and diversity between subtype B and the other subtypes/groups (red). The x- and y-axes indicate the HIV amino

acid diversity and the proportions, respectively. (B) Distribution plots of amino acid diversity between subtype C and other clades (HIV-1 subtypes,HIV-1 and HIV-2 groups).

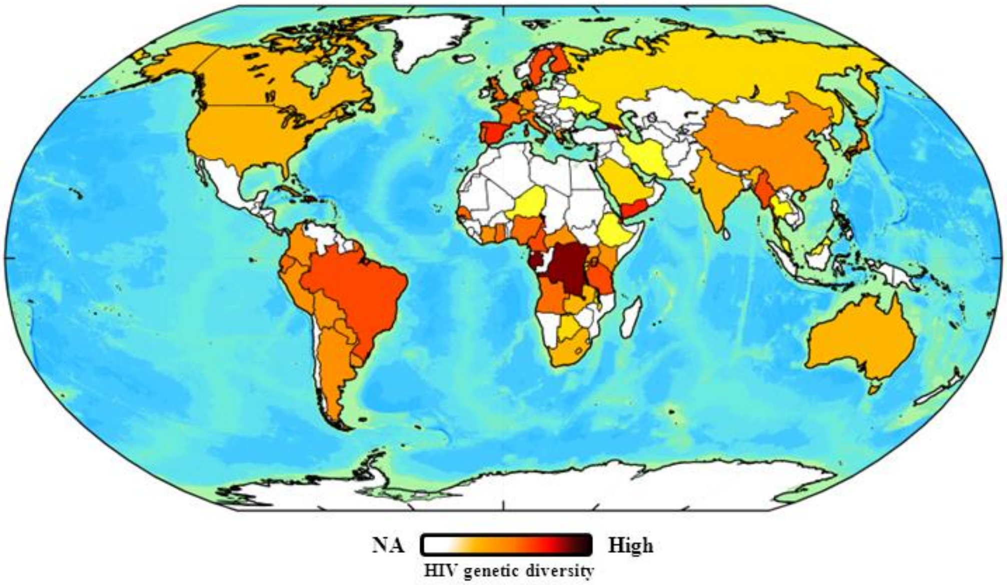

Figure S 4: Geographic distribution of HIV-1 genomic diversity. Countries with no sequences available (NA) are colored white. Amino acid diversity in individual countries was mapped onto the global cartographic map in Natural Earth V2.0.0 (http://www.naturalearthdata.com/). Countries with their infections in different groups or subtypes had higher genomic diversity, and the highest being found in Central Africa. Our results are consistent with the reported distribution of HIV-1 subtypes described in [2], implying that strains included in our study may capture the global HIV-1 diversity.

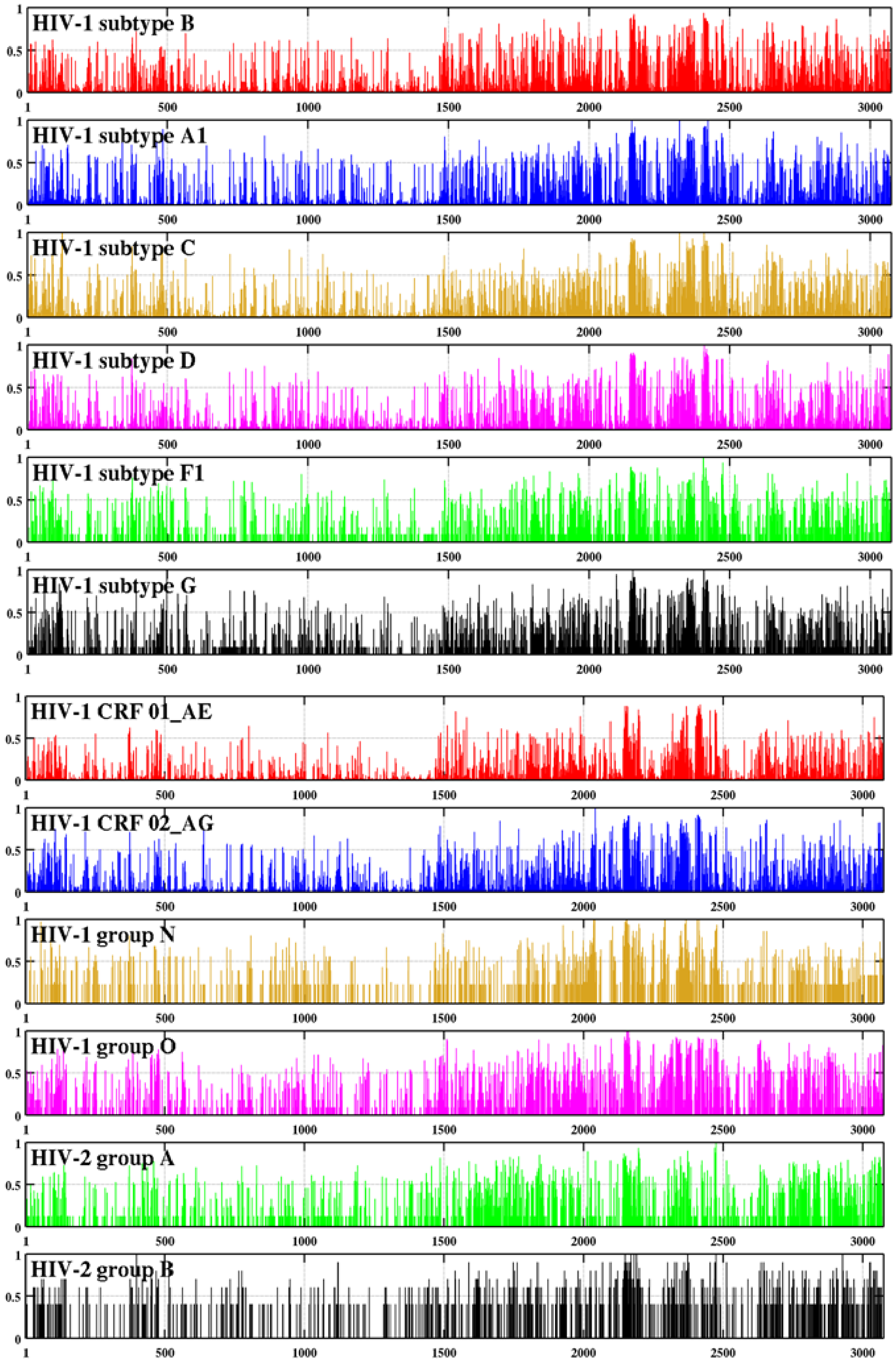

Figure S 5: Amino acid diversity along the full-length HIV genome. Twelve subplots individually show the nucleotide diversity results for subtype B, A1, C, D, F1, G, CRF01_AE, CRF02_AG, and HIV-2 group A and B, HIV-1 group N, O.

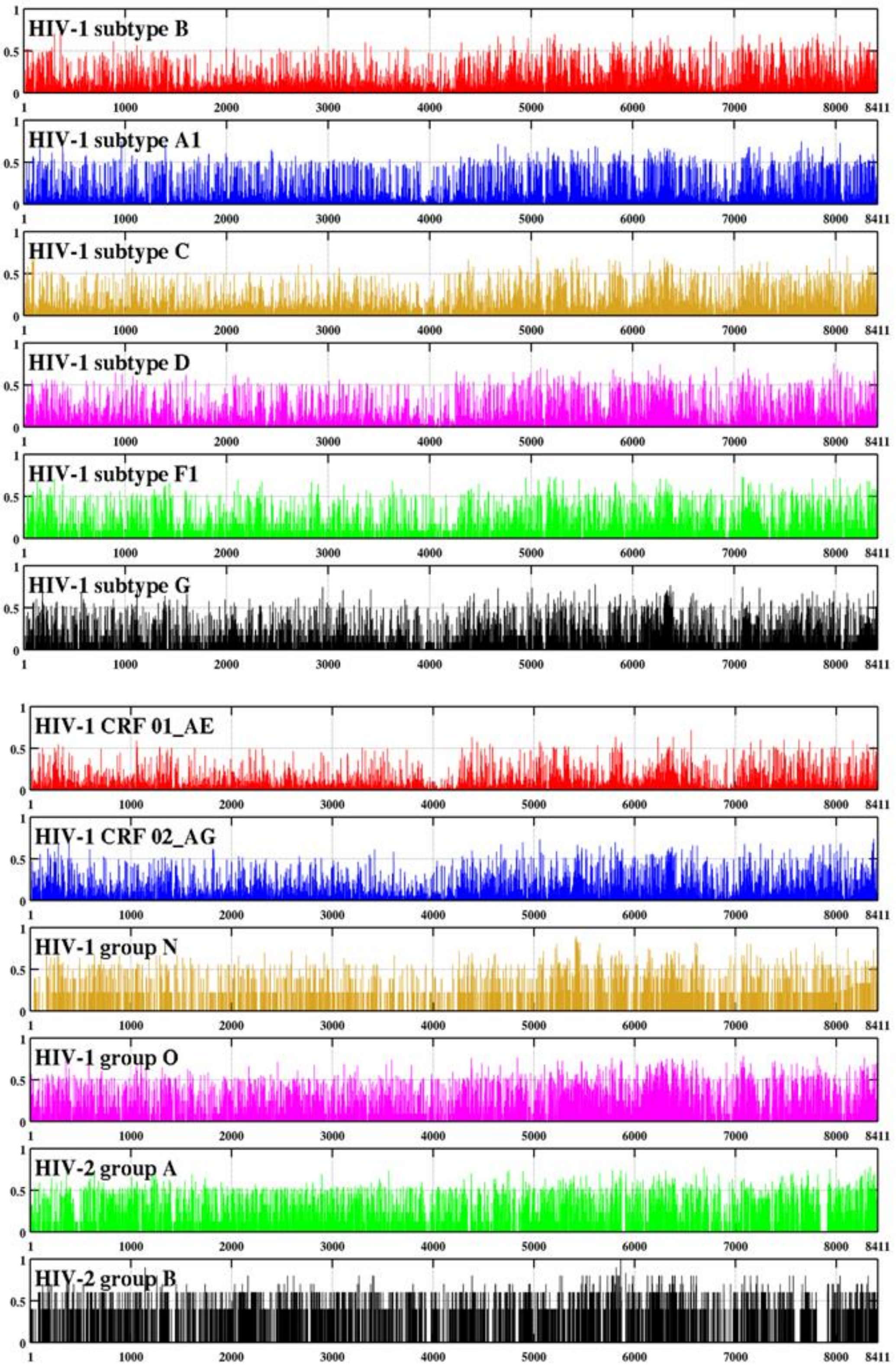

Figure S 6: Nucleotide diversity along the full-length HIV genome. Twelve subplots individually show the nucleotide diversity results for subtype B, A1, C, D, F1, G, CRF01_AE, CRF02_AG, and HIV-2 group A and B, HIV-1 group N, O.

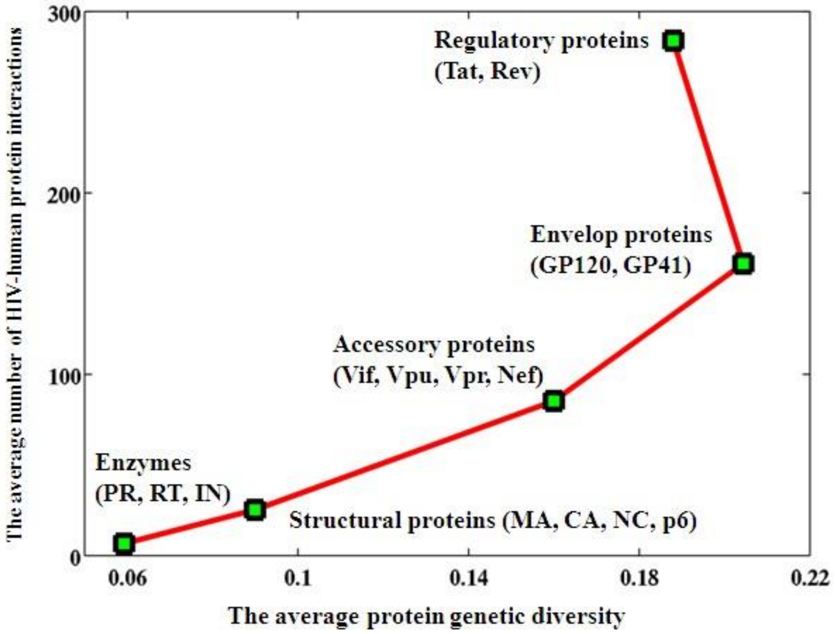

Figure S 7: Average amino acid diversity of HIV-1 protein clusters and number of HIV-human protein interactions. Five protein clusters include: viral enzymes (PR, RT, IN), accessory proteins (Vif, Vpu, Vpr, Nef), envelope proteins (GP120, GP41) and regulatory proteins (Tat, Rev). Proteins are clustered according to their functional roles in the HIV-1 life cycle [3].

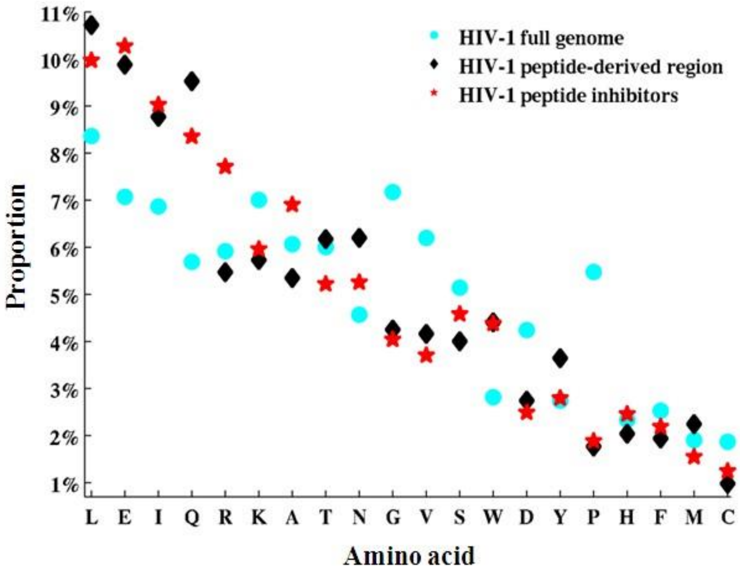

Figure S 8: Amino acid composition of HIV-1 subtype B genome (blue circles), HIV-1 peptide-derived regions (black diamonds) and sequences of HIV-derived peptide inhibitors (red stars).

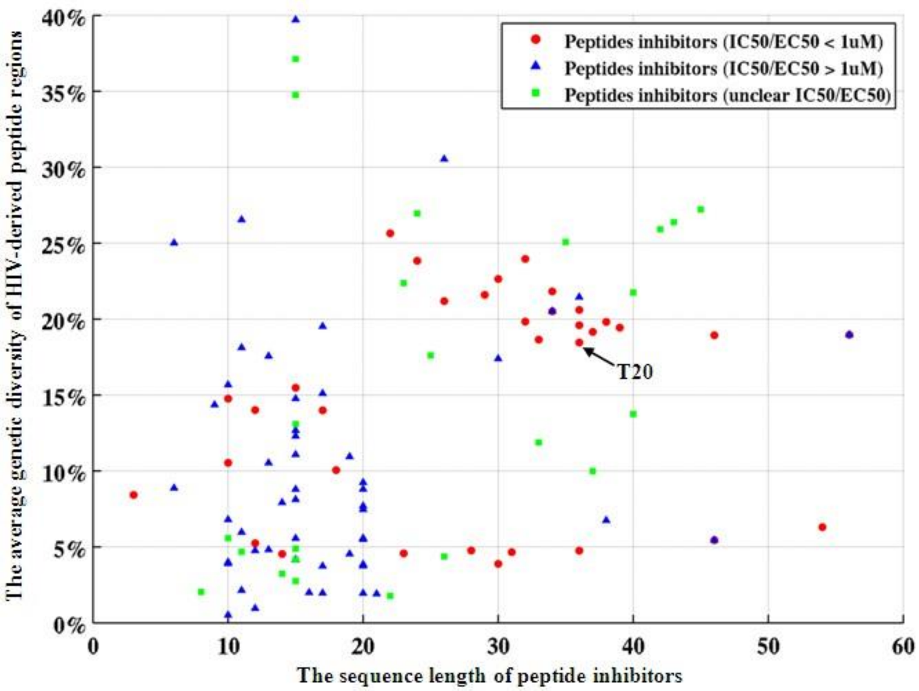

Figure S 9: Average amino acid diversity of peptide-derived regions in the HIV-1 subtype B genome. X-axis indicates the length of peptide inhibitor sequences. Y-axis indicates the average genetic diversity of the known peptide-derived regions in the HIV-1 subtype B genome.

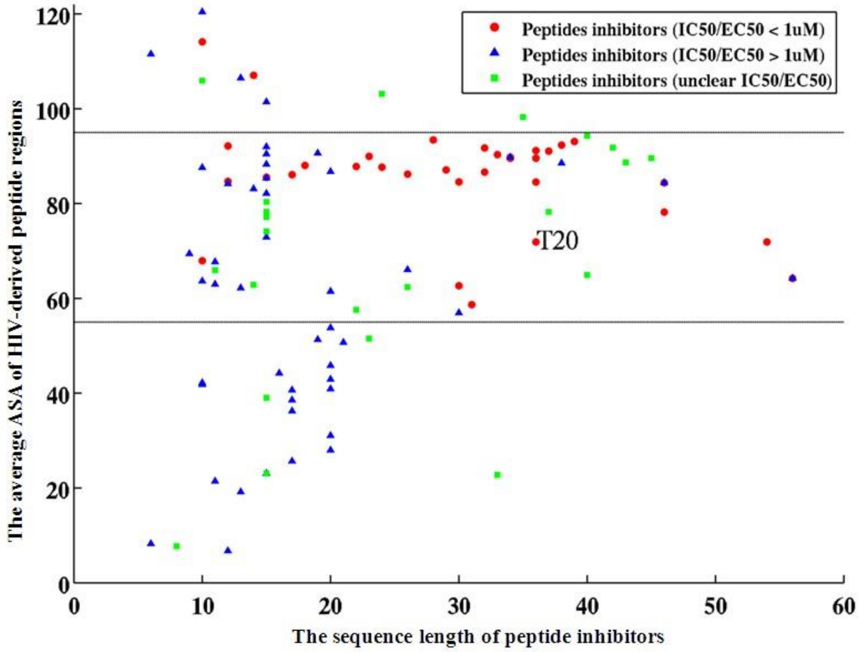

Figure S 10: Solvent accessible surface area (ASA) of peptide-derived regions in the HIV-1 subtype B genome. X-axis shows the length of peptide inhibitor sequences. Y-axis shows the average ASA (Å) of known peptide-derived regions in the HIV-1 subtype B genome. Horizontal lines mark the average ASA between 55 Å and 95 Å, covering most peptide inhibitors with IC50/EC50<1μM.

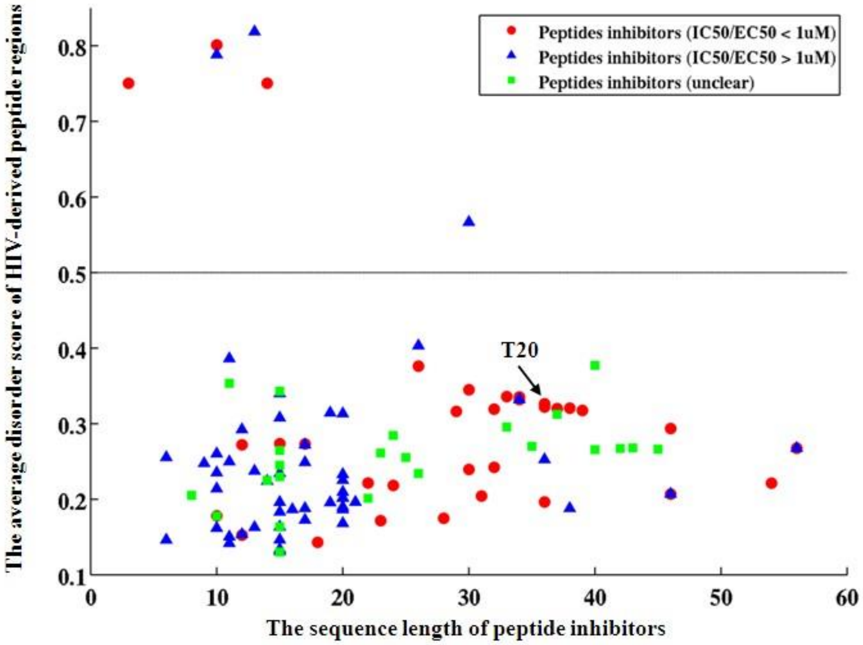

Figure S 11: Protein intrinsic disorder scores of peptide-derived regions in the HIV-1 subtype B genome. X-axis indicates the length of HIV-derived peptide inhibitor sequences. Y-axis indicates the average protein intrinsic disorder scores in the known peptide-derived regions of HIV-1 subtype B genome. The horizontal line at the value of 0.5 indicates the cutoff of the protein intrinsic disorder score for determining disordered (≥0.5) or ordered (<0.5) structural regions.

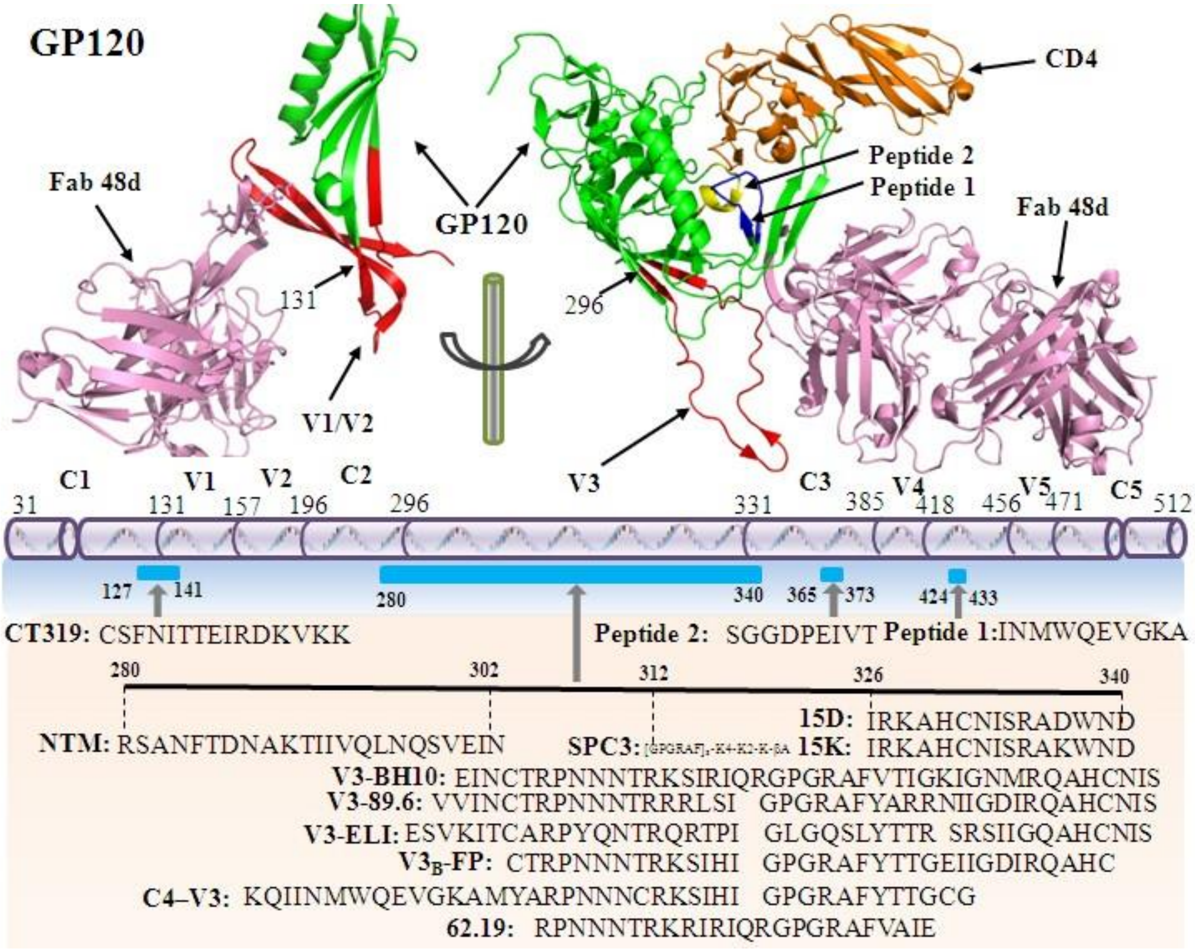

Figure S 12: Protein structure of the HIV-1 GP120-CD4-Fab 48d complex (PDB: 2B4C, 3U4E) and mapped GP120 peptide-derived inhibitors. On the structure, CD4 and Fab 48d structures are colored orange and pink, respectively. The GP120 and peptide inhibitor sequences are annotated beneath the protein structures. Peptide inhibitors are mapped to the GP120 functional domains (bottom), including 5 variable domains (V1-V5) and 5 conserved domains (C1-C5) [4].

The V1 to V3 and V5 loops have been identified as the minimal functional units of GP120 to mediate CXCR4-dependent infection [4]. The V3 loop is the major target for neutralizing antibodies and V3-derived peptides offer promising anti-HIV activities [5]. GP120-derived peptides can inhibit the interactions between GP120 and T-cell surface glycoproteins (e.g. CD4, CD19), chemokine co-receptors (e.g. CCR5, CXCR4) and monoclonal antibodies [5]. The inhibition activity of GP120-derived peptides is strain-dependent and cell-dependent (Table S4).

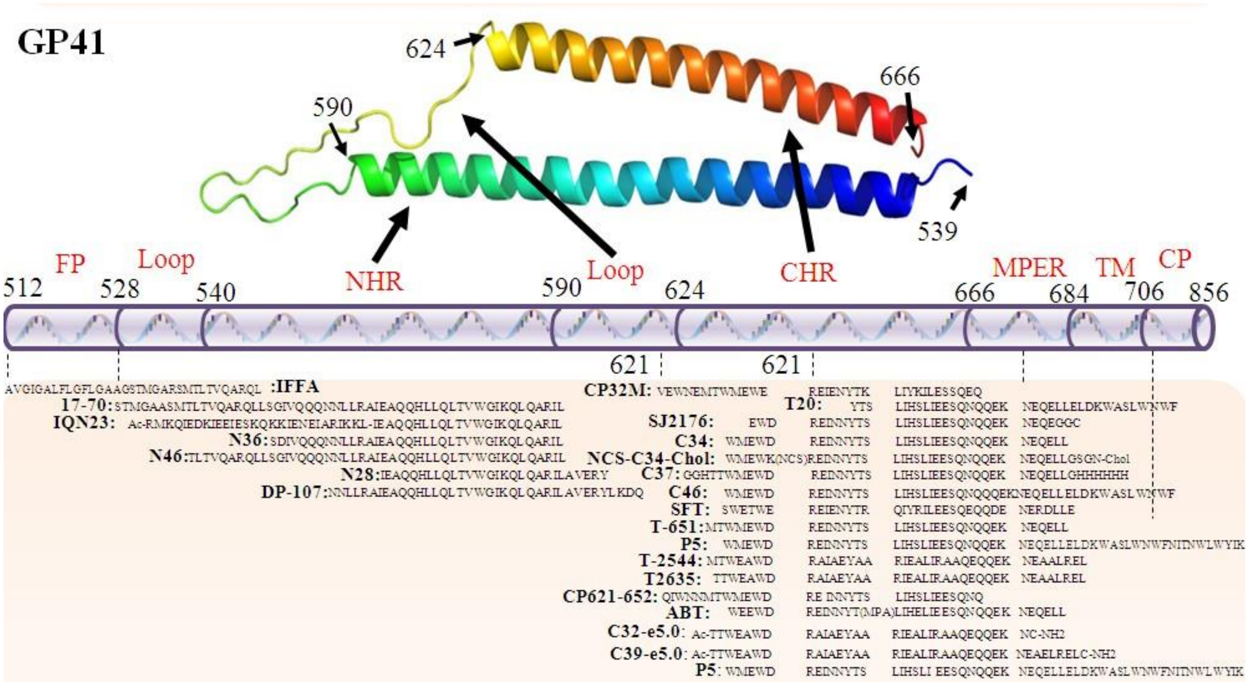

Figure S 13: GP41 structure and mapped GP41-derived peptide inhibitors. The protein cartoon representation of GP41 protein is shown on top and the peptide inhibitor sequences are annotated beneath the structures. SIV GP41 structure is visualized (PDB: 2EZS) as the HIV-1 GP41 crystal structure has not been fully resolved (February 2014).

GP41 forms a trimeric spike with GP120 on the surface of HIV particles (Figure 4). Three C-terminal heptad repeats (CHRs) bind with three N-terminal heptad repeats (NHRs) to form a 6-helix bundle, which fuses the particle with cellular membrane to create a fusion pore during the viral entry [6, 7]. Peptides derived from either NHR or CHR can mimic the viral structures to prevent viral entry. T20 (Enfuvirtide, Fuzeon, DP178), derived from NHR of HIV-1 subtype B strain LAI, is an 36AA L-peptide inhibitor which targets multiple sites on GP41 and GP120 [8]. Most GP41 peptide inhibitors have been derived from the conserved pre-hairpin structure [9]. GP41-derived L-peptides have many defects such as proteolytic degradation, limited potency and toxicity [10]. To overcome the defects of L-peptides, D-peptides have been proposed as promising fusion inhibitors (see reviews in [11, 12]). For instance, D-peptide inhibitors PIE12 [13] and IQN17 [14] have shown promising anti-HIV activities by mimicking the GP41 heptad repeat regions.

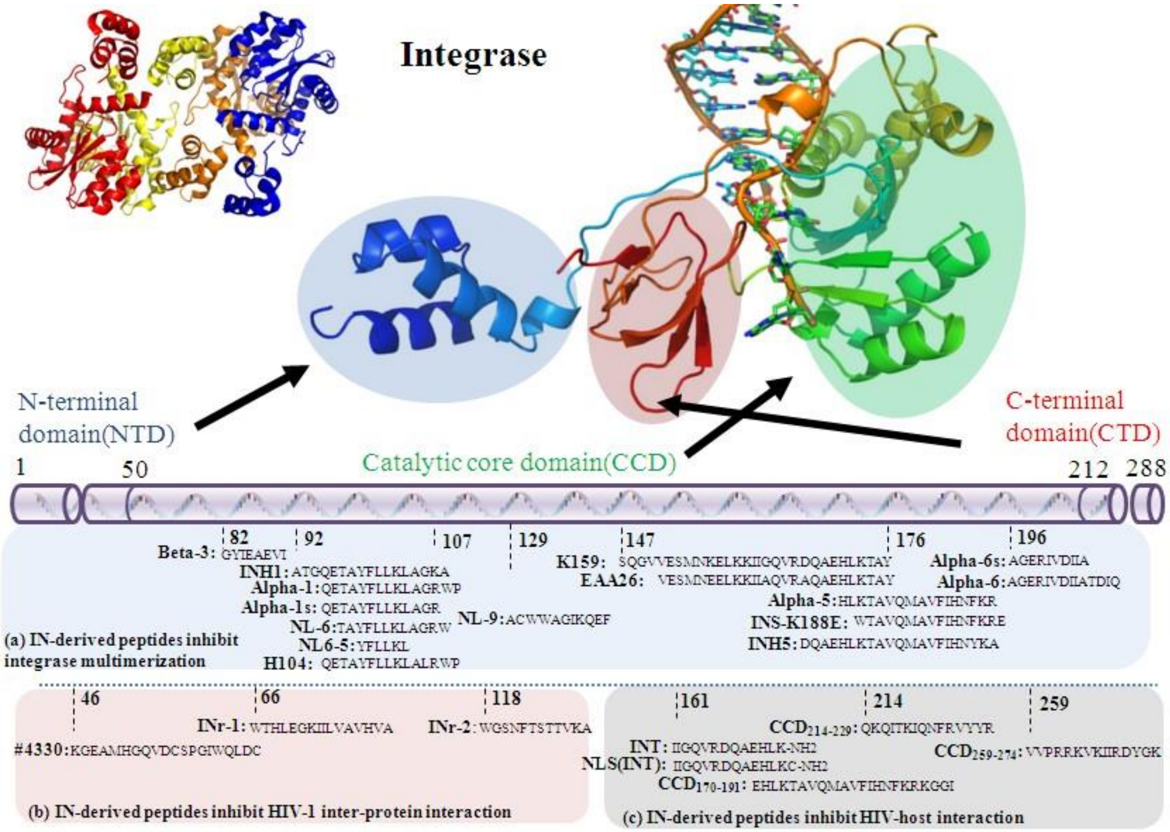

Figure S 14: HIV-1 Integrase tetramer (PDB: 1K6Y) and mapped Integrase-derived peptide inhibitors. Cartoon representation of Integrase domains is shown on top and the peptide inhibitor sequences are annotated beneath the structures. For visualization purpose, we used the Integrase monomer from prototype foamy virus (PDB: 3OY9).

The HIV-1 Integrase structure has an N-terminal domain (NTD), a catalytic core domain (CCD) and a C-terminal domain (CTD), connected by flexible links [3]. Integrase plays multiple roles during the viral reverse transcription and integration [15]. The key functional roles of Integrase is to insert viral dsDNA into human chromatins, creating a viral reservoir for viral infection [16]. Integrase-derived peptides can inhibit Integrase-mediated catalytic functions, Integrase inter-domain interactions and/or Integrase-human protein interactions (Table S4). Peptide inhibitors derived from CCD can inhibit Integrase dimerization, 3'-end DNA processing and strand transfer during the viral integration [17].

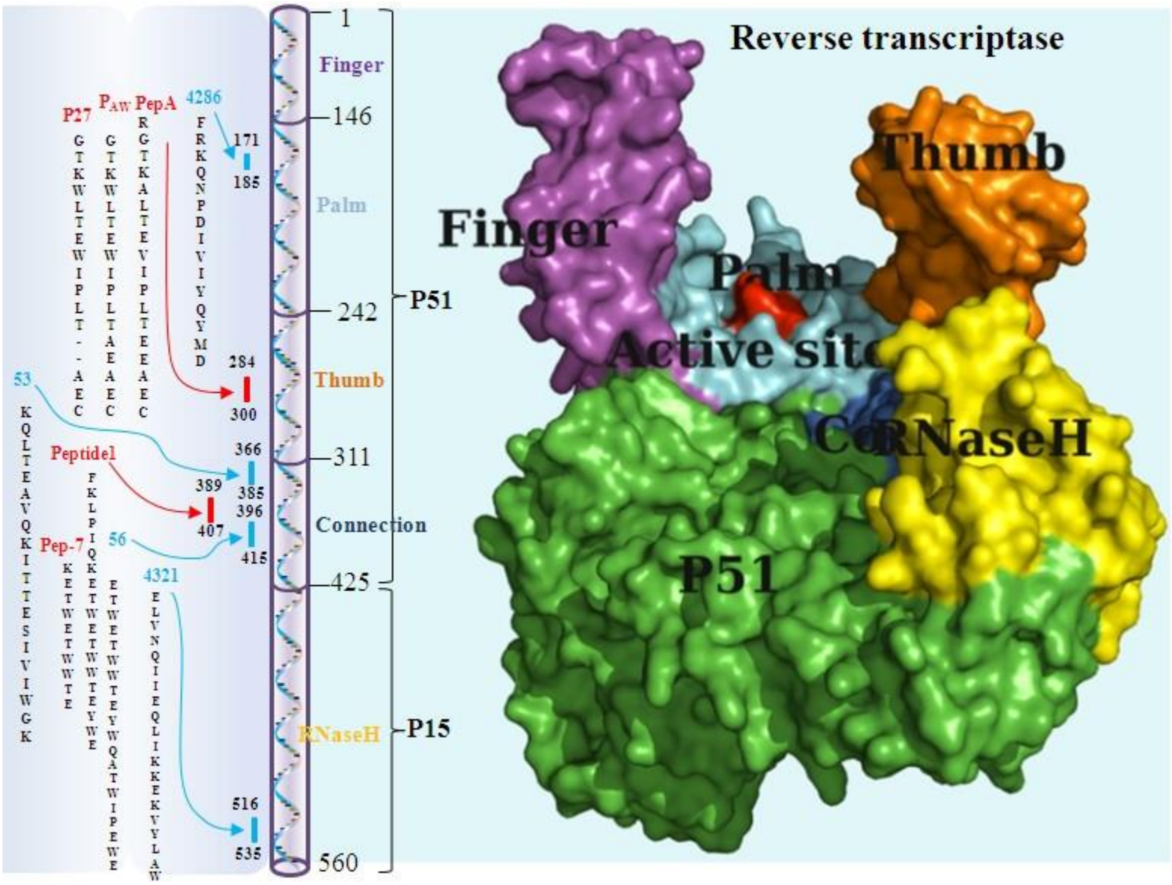

Figure S 15: HIV-1 reverse transcriptase (RT) structure (PDB: 3DLK) and mapped RT-derived peptide inhibitors. The surface representation of reverse transcriptase domains is shown on the right side, and HIV-derived peptide inhibitor sequences are annotated on the left side.

Reverse transcriptase forms a heterodimer to synthesize HIV dsDNA from the viral genomic RNA [18]. RT structures are comprised of the finger, palm, thumb, connection, RNaseH and P51 functional domains [19]. Peptide inhibitors derived from the connection (Pep-7 [20], Peptide1 [21]) and the thumb domain (P24[22], P27[22], P_{AW}[22]) can block the dimerization of p66 and p51. Nanoparticle systems can improve the delivery of RT peptide inhibitors [22].

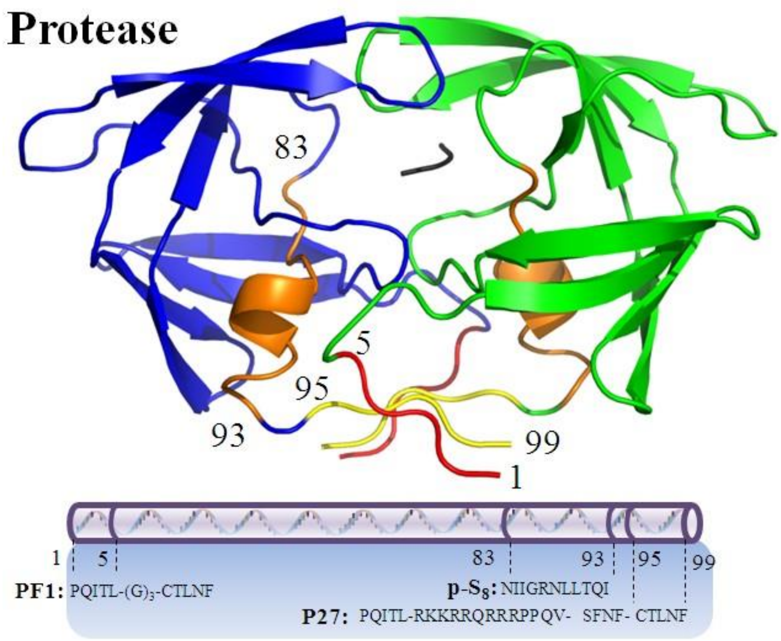

Figure S 16: HIV-1 Protease homodimer structure (PDB:1A30) and mapped protease-derived peptide inhibitors. Cartoon representation of protease is shown on top, and the peptide inhibitor sequences are annotated beneath the structure.

Beta-sheets of the N-terminal and C-terminal domains are crucial for protease dimerization [23, 24]. HIV-derived peptide inhibitors that mimic the N- and C-terminal domains have been investigated as potential protease inhibitors. These include the cross linked interfacial peptide PF1 [25] and the PR-derived peptide p-S8 [26]. Peptides derived from protease positions 83-93 can also inhibit the protease folding [27-29].

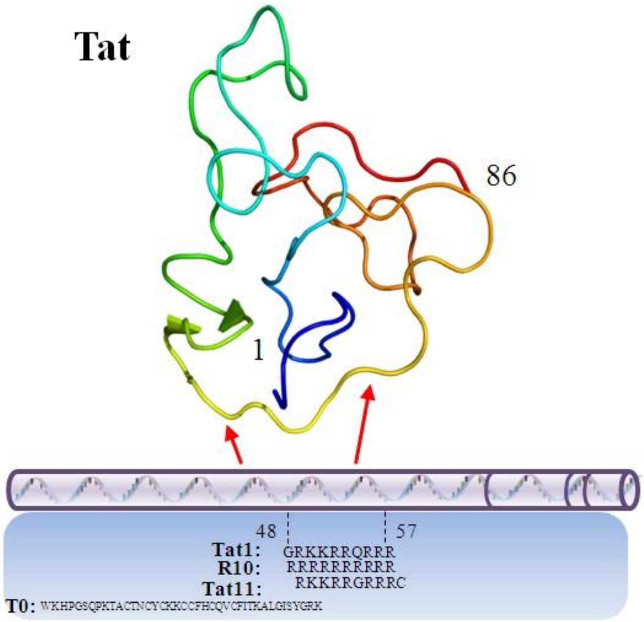

Figure S 17: HIV-1 Tat structure (PDB: 1JFW) and mapped Tat-derived peptide inhibitors. Cartoon representation of Tat is shown on top. Peptide inhibitor sequences are annotated beneath the structure.

The regulatory protein Tat can bind with GP120 to enhance the viral entry [30]. Peptide sequences derived from Tat positions 48-57 can interrupt the Tat-GP120 interaction in a concentration-dependent manner [30]. The peptide inhibitor Tat11 can interrupt nuclear import by interacting with the host importin beta protein [31]. Moreover, the Tat-mediated transcription can be inhibited by interrupting Tat-TAR interactions, which involves the arginine rich motif of Tat and the 3-nt bulge of the TAR RNA hairpin (U23, A27, U38) [32].

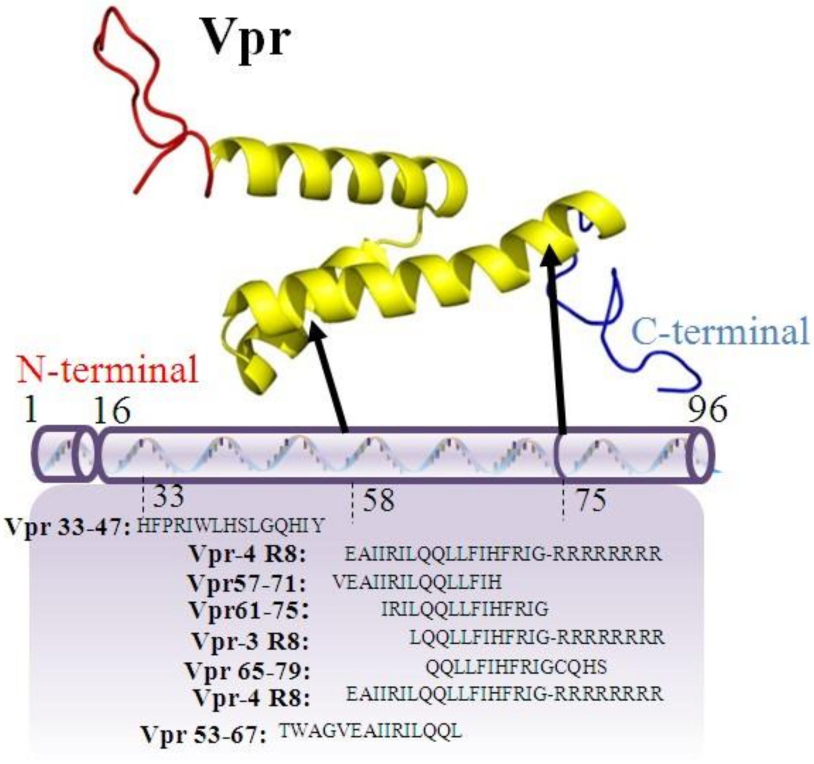

Figure S 18: HIV-1 Vpr structure (PDB:1M8L) and mapped Vpr-derived peptides. Cartoon representation of Vpr is shown on top and HIV-derived peptide inhibitor sequences are annotated beneath the Vpr structure.

An interaction between Vpr and RT has not been reported, nor an interaction between Vpr and Integrase. However, peptide inhibitors derived from Vpr domains (positions: 57-71, 61-75) can interfere with the activity of both RT and Integrase [33]. Two studies have shown that Vpr-derived peptides (positions: 55-69, 60-74) can inhibit the strand transfer and the 3'-end-processing reactions performed by Integrase [34, 35].

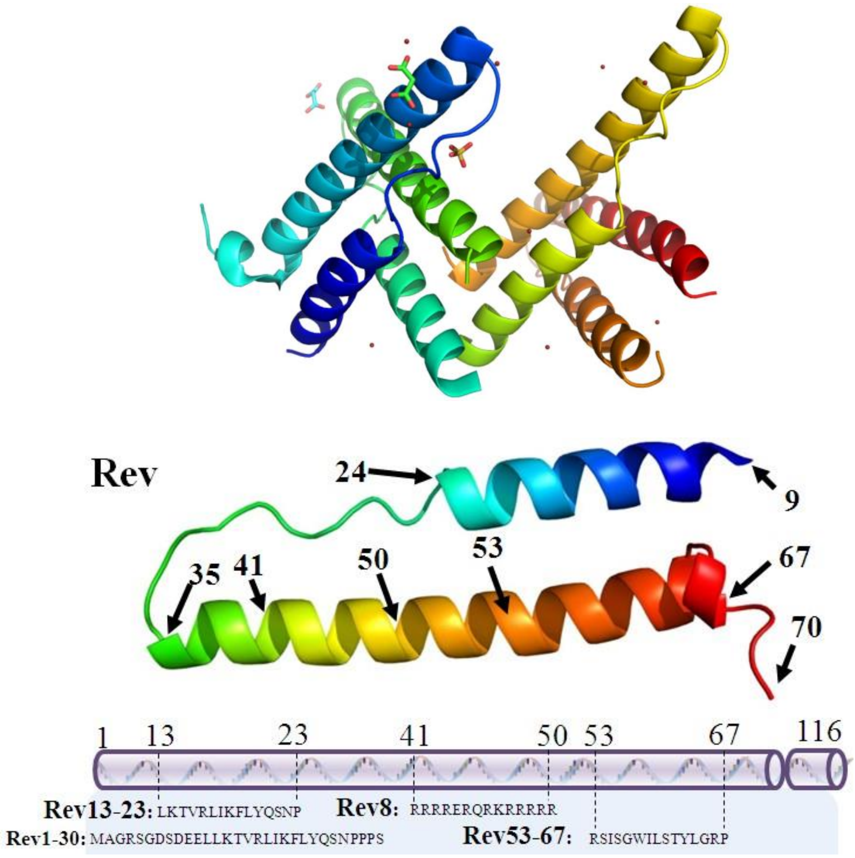

Figure S 19: HIV-1 Rev tetramer structure (PDB: 3LPH) and mapped Rev-derived peptide inhibitors. Cartoon representation of Rev is shown on top and Rev-derived peptide inhibitor sequences are annotated beneath the Rev structure.

Rev can target the Rev response element (RRE) in the viral RNA genome during nuclear export, while Rev-derived peptides can interrupt the Rev-RRE interaction [36]. Rev can physically bind with Integrase to form a pre-integration complex so that viral integration can be postponed until the completion of nucleocytoplasmic shuttling [37, 38]. Two Rev-derived peptides (positions: 1-30, 49-74) can inhibit the Integrase 3'-end processing and the strand-transfer in cell-free assays [37]. Moreover, direct interactions between two Rev domains (positions: 12-23, 53-67) and Integrase domains (positions: 118-128, 66-80) have been reported [39]. Two shorter Rev peptides (positions 13-23, 53-67) have also shown the inhibitory activity [40]. The Integrase-derived peptides INr-1 and INr-2 can stimulate viral genome integration and interrupt the Rev-Integrase protein interaction [41].

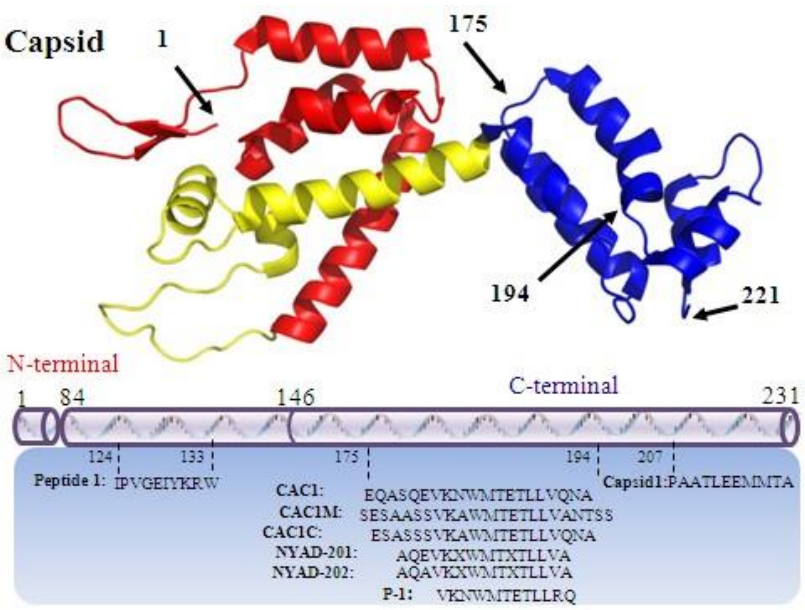

Figure S 20: Monomer structure of HIV-1 Capsid (PDB: 2NTE) and mapped Capsid-derived peptide inhibitors. Cartoon representation of Capsid is shown on top and HIV-derived peptide inhibitor sequences are annotated beneath the protein structure.

Capsid pentamers and hexamers constitute the internal shell of viral particles [42]. The alpha-helical structure of the C-terminal domain (CTD, positions: 146-231) participates in the capsid multimerization [43]. Peptide inhibitors derived from the CTD can interrupt the multimerization of HIV-1 Capsid by mimicking the capsid multimerization interfaces. The peptide inhibitor CAC1 derived from the CTD domain can disassociate CTD dimers (K_d = 50 uM) [44]. Since peptide inhibitors must penetrate the viral membrane to prevent Capsid multimerization, cell-penetrating peptides have been designed to improve the peptide potency in cell culture experiments [45, 46]. For instance, peptide inhibitor CAI [47] has been converted into a cell-penetrating peptide NYAD-1, which improves the binding affinity and inhibits the post entry stage [45]. Cell-penetrating peptides NYAD-201 and NYAD-202 have shown promising anti-HIV activities [46].

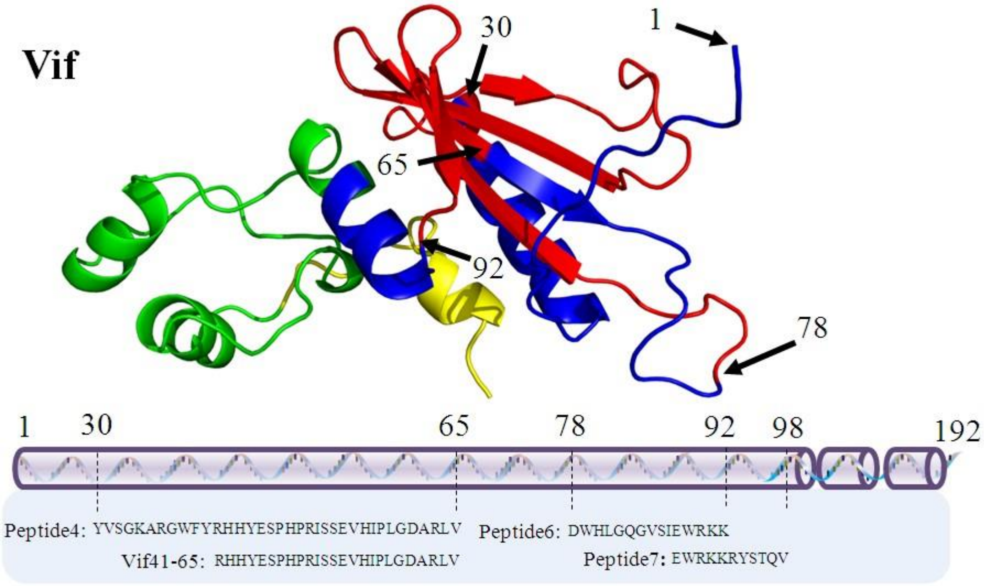

Figure S 21: HIV-1 Vif structure (PDB: 4N9F) and mapped Vif-derived peptide inhibitors. Peptide inhibitor sequences are annotated beneath the Vif structure. The N-terminal domain, the central domain and the C-terminal domain of Vif are colored blue, green and yellow, respectively.

Vif-derived peptide Vif41-65 (positions: 41-65) can inhibit the protease activity [48]. Vif positions (36, 47, 101, 117, 124) are associated with PI treatment [49]. Two Vif-derived peptides 30-65 and 78-98 have also been shown to inhibit the protease activity [50]. The N terminus of Protease (positions: 1-9) interacts with the central domain of Vif (positions: 78-98) [51]. Two Vif-derived peptides (positions: 81-88, 88-98) can inhibit the protease activity [50, 52].

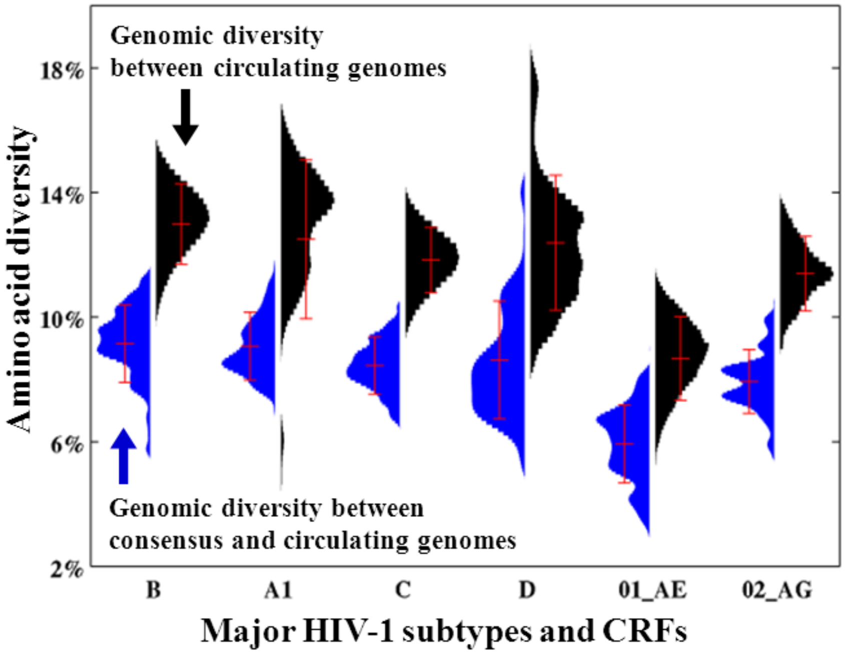

Figure S 22: Distribution plots of amino acid diversity between the consensus and the circulating genomes (blue), and within circulating genomes (black). X-axis indicates HIV-1 subtypes B, A1, C, D, CRF01_AE and CRF02_AG, each of which contains more than 50 sequences in our datasets. Y-axis indicates the amino acid genomic diversity. For each subtype, the consensus sequence is obtained by retaining the most prevalent amino acid at each position. For the 6 major HIV-1 subtypes and CRFs (A1, B, C, D, 01_AE, 02_AG), the average amino acid diversity between circulating strains (12.3 ± 1.5%) was significantly higher than that between the consensus and the circulating strains (8.3 ± 1.3%, P-value < 0.001).

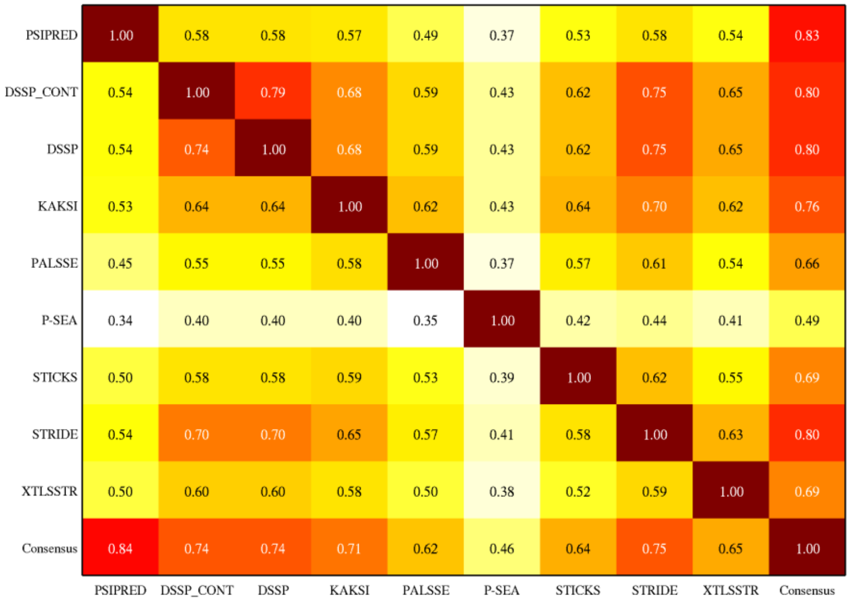

Figure S 23: Similarity of prediction results between the consensus and the 9 protein secondary structure prediction methods. Consensus predictions were obtained using the majority voting strategy among 9 individual methods. Given 15 HIV-1 proteins in the full-length genome of HIV-1 subtype B, similarities between two methods were calculated by the percentages of common predictions as alpha-helix (top-right part of matrix) and beta-strand (left-bottom part of matrix) structures.

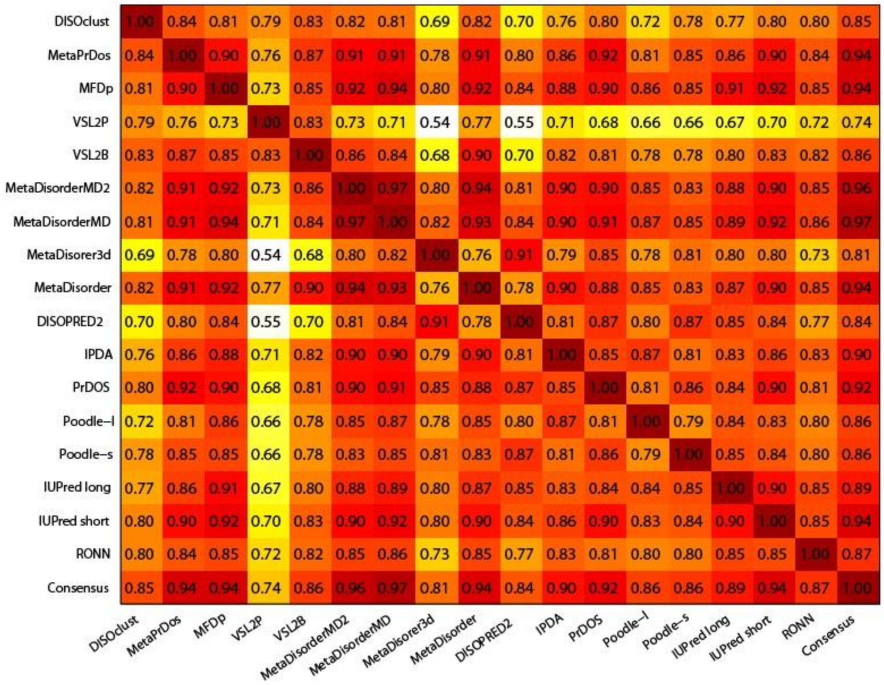

Figure S 24: Prediction similarities of the consensus and 17 methods for protein intrinsically disorder prediction. Prediction similarities were calculated by the

percentages of common predictions of ordered (disorder tendency score < 0.5) or disordered (disorder tendency score ≥ 0.5) positions in the HIV-1 protein structures. Consensus predictions were obtained using the majority voting strategy among the 17 individual methods. The consensus method has the highest average prediction similarities compared to the other methods.

References

1. Binley JM, Sanders RW, Master A, Cayanan CS, Wiley CL, Schiffner L, Travis B, Kuhmann S, Burton DR, Hu SL, et al: **Enhancing the proteolytic maturation of human immunodeficiency virus type 1 envelope glycoproteins.** *J Virol* 2002, **76:**2606-2616.
2. Hemelaar J, Gouws E, Ghys PD, Osmanov S, Isolation W-UNfH, Characterisation: **Global trends in molecular epidemiology of HIV-1 during 2000-2007.** *AIDS* 2011, **25:**679-689.
3. Engelman A, Cherepanov P: **The structural biology of HIV-1: mechanistic and therapeutic insights.** *Nat Rev Microbiol* 2012, **10:**279-290.
4. Ghaffari G, Tuttle DL, Briggs D, Burkhardt BR, Bhatt D, Andiman WA, Sleasman JW, Goodenow MM: **Complex determinants in human immunodeficiency virus type 1 envelope gp120 mediate CXCR4-dependent infection of macrophages.** *J Virol* 2005, **79:**13250-13261.
5. Moseri A, Tantry S, Sagi Y, Arshava B, Naider F, Anglister J: **An optimally constrained V3 peptide is a better immunogen than its linear homolog or HIV-1 gp120.** *Virology* 2010, **401:**293-304.
6. Frey G, Chen J, Rits-Volloch S, Freeman MM, Zolla-Pazner S, Chen B: **Distinct conformational states of HIV-1 gp41 are recognized by neutralizing and non-neutralizing antibodies.** *Nat Struct Mol Biol* 2010, **17:**1486-1491.
7. Chen X, Lu L, Qi Z, Lu H, Wang J, Yu X, Chen Y, Jiang S: **Novel recombinant engineered gp41 N-terminal heptad repeat trimers and their potential as anti-HIV-1 therapeutics or microbicides.** *J Biol Chem* 2010, **285:**25506-25515.
8. Liu S, Lu H, Niu J, Xu Y, Wu S, Jiang S: **Different from the HIV fusion inhibitor C34, the anti-HIV drug Fuzeon (T-20) inhibits HIV-1 entry by targeting multiple sites in gp41 and gp120.** *J Biol Chem* 2005, **280:**11259-11273.
9. Champagne K, Shishido A, Root MJ: **Interactions of HIV-1 inhibitory peptide T20 with the gp41 N-HR coiled coil.** *J Biol Chem* 2009, **284:**3619-3627.
10. Welch BD, VanDemark AP, Heroux A, Hill CP, Kay MS: **Potent D-peptide inhibitors of HIV-1 entry.** *Proc Natl Acad Sci U S A* 2007, **104:**16828-16833.
11. Pang W, Tam SC, Zheng YT: **Current peptide HIV type-1 fusion inhibitors.** *Antivir Chem Chemother* 2009, **20:**1-18.
12. Ashkenazi A, Shai Y: **Insights into the mechanism of HIV-1 envelope induced membrane fusion as revealed by its inhibitory peptides.** *Eur Biophys J* 2011, **40:**349-357.
13. Welch BD, Francis JN, Redman JS, Paul S, Weinstock MT, Reeves JD, Lie YS, Whitby FG, Eckert DM, Hill CP, et al: **Design of a potent D-peptide HIV-1**

entry inhibitor with a strong barrier to resistance. *J Virol* 2010, **84:**11235-11244.

14. Eckert DM, Malashkevich VN, Hong LH, Carr PA, Kim PS: **Inhibiting HIV-1 entry: discovery of D-peptide inhibitors that target the gp41 coiled-coil pocket.** *Cell* 1999, **99:**103-115.
15. Poeschla EM: **Integrase, LEDGF/p75 and HIV replication.** *Cell Mol Life Sci* 2008, **65:**1403-1424.
16. Craigie R, Bushman FD: **HIV DNA Integration.** *Cold Spring Harb Perspect Med* 2012, **2:**a006890.
17. Maes M, Loyter A, Friedler A: **Peptides that inhibit HIV-1 integrase by blocking its protein-protein interactions.** *FEBS J* 2012, **279:**2795-2809.
18. La Regina G, Coluccia A, Silvestri R: **Looking for an active conformation of the future HIV type-1 non-nucleoside reverse transcriptase inhibitors.** *Antivir Chem Chemother* 2010, **20:**213-237.
19. Huang H, Chopra R, Verdine GL, Harrison SC: **Structure of a covalently trapped catalytic complex of HIV-1 reverse transcriptase: implications for drug resistance.** *Science* 1998, **282:**1669-1675.
20. Depollier J, Hourdou ML, Aldrian-Herrada G, Rothwell P, Restle T, Divita G: **Insight into the mechanism of a peptide inhibitor of HIV reverse transcriptase dimerization.** *Biochemistry* 2005, **44:**1909-1918.
21. Divita G, Restle T, Goody RS, Chermann JC, Baillon JG: **Inhibition of human immunodeficiency virus type 1 reverse transcriptase dimerization using synthetic peptides derived from the connection domain.** *J Biol Chem* 1994, **269:**13080-13083.
22. Agopian A, Gros E, Aldrian-Herrada G, Bosquet N, Clayette P, Divita G: **A new generation of peptide-based inhibitors targeting HIV-1 reverse transcriptase conformational flexibility.** *J Biol Chem* 2009, **284:**254-264.
23. Ishima R, Torchia DA, Louis JM: **Mutational and structural studies aimed at characterizing the monomer of HIV-1 protease and its precursor.** *J Biol Chem* 2007, **282:**17190-17199.
24. Sperka T, Boross P, Eizert H, Tozser J, Bagossi P: **Effect of mutations on the dimer stability and the pH optimum of the human foamy virus protease.** *Protein Eng Des Sel* 2006, **19:**369-375.
25. Bowman MJ, Chmielewski J: **Novel strategies for targeting the dimerization interface of HIV protease with cross-linked interfacial peptides.** *Biopolymers* 2002, **66:**126-133.
26. Bonomi M, Barducci A, Gervasio FL, Parrinello M: **Multiple routes and milestones in the folding of HIV-1 protease monomer.** *PLoS One* 2010, **5:**e13208.
27. Broglia RA, Provasi D, Vasile F, Ottolina G, Longhi R, Tiana G: **A folding inhibitor of the HIV-1 protease.** *Proteins* 2006, **62:**928-933.
28. Bonomi M, Gervasio FL, Tiana G, Provasi D, Broglia RA, Parrinello M: **Insight into the folding inhibition of the HIV-1 protease by a small peptide.** *Biophys J* 2007, **93:**2813-2821.

29. Broglia RA, Tiana G, Sutto L, Provasi D, Simona F: **Design of HIV-1-PR inhibitors that do not create resistance: blocking the folding of single monomers.** *Protein Sci* 2005, **14:**2668-2681.
30. Keogan S, Passic S, Krebs FC: **Infection by CXCR4-Tropic Human Immunodeficiency Virus Type 1 Is Inhibited by the Cationic Cell-Penetrating Peptide Derived from HIV-1 Tat.** *Int J Pept* 2012, **2012:**349427.
31. Friedler A, Friedler D, Luedtke NW, Tor Y, Loyter A, Gilon C: **Development of a functional backbone cyclic mimetic of the HIV-1 Tat arginine-rich motif.** *J Biol Chem* 2000, **275:**23783-23789.
32. Davidson A, Leeper TC, Athanassiou Z, Patora-Komisarska K, Karn J, Robinson JA, Varani G: **Simultaneous recognition of HIV-1 TAR RNA bulge and loop sequences by cyclic peptide mimics of Tat protein.** *Proc Natl Acad Sci U S A* 2009, **106:**11931-11936.
33. Gleenberg IO, Herschhorn A, Hizi A: **Inhibition of the activities of reverse transcriptase and integrase of human immunodeficiency virus type-1 by peptides derived from the homologous viral protein R (Vpr).** *J Mol Biol* 2007, **369:**1230-1243.
34. Suzuki S, Maddali K, Hashimoto C, Urano E, Ohashi N, Tanaka T, Ozaki T, Arai H, Tsutsumi H, Narumi T, et al: **Peptidic HIV integrase inhibitors derived from HIV gene products: structure-activity relationship studies.** *Bioorg Med Chem* 2010, **18:**6771-6775.
35. Suzuki S, Urano E, Hashimoto C, Tsutsumi H, Nakahara T, Tanaka T, Nakanishi Y, Maddali K, Han Y, Hamatake M, et al: **Peptide HIV-1 integrase inhibitors from HIV-1 gene products.** *J Med Chem* 2010, **53:**5356-5360.
36. Mills NL, Daugherty MD, Frankel AD, Guy RK: **An alpha-helical peptidomimetic inhibitor of the HIV-1 Rev-RRE interaction.** *J Am Chem Soc* 2006, **128:**3496-3497.
37. Rosenbluh J, Hayouka Z, Loya S, Levin A, Armon-Omer A, Britan E, Hizi A, Kotler M, Friedler A, Loyter A: **Interaction between HIV-1 Rev and integrase proteins: a basis for the development of anti-HIV peptides.** *J Biol Chem* 2007, **282:**15743-15753.
38. Levin A, Hayouka Z, Friedler A, Loyter A: **Nucleocytoplasmic shuttling of HIV-1 integrase is controlled by the viral Rev protein.** *Nucleus* 2010, **1:**190-201.
39. Levin A, Rosenbluh J, Hayouka Z, Friedler A, Loyter A: **Integration of HIV-1 DNA is regulated by interplay between viral rev and cellular LEDGF/p75 proteins.** *Mol Med* 2010, **16:**34-44.
40. Hayouka Z, Rosenbluh J, Levin A, Maes M, Loyter A, Friedler A: **Peptides derived from HIV-1 Rev inhibit HIV-1 integrase in a shiftide mechanism.** *Biopolymers* 2008, **90:**481-487.
41. Levin A, Hayouka Z, Helfer M, Brack-Werner R, Friedler A, Loyter A: **Peptides derived from HIV-1 integrase that bind Rev stimulate viral genome integration.** *PLoS One* 2009, **4:**e4155.
42. Pornillos O, Ganser-Pornillos BK, Kelly BN, Hua Y, Whitby FG, Stout CD, Sundquist WI, Hill CP, Yeager M: **X-ray structures of the hexameric building block of the HIV capsid.** *Cell* 2009, **137:**1282-1292.

43. Domenech R, Bocanegra R, Gonzalez-Muniz R, Gomez J, Mateu MG, Neira JL: **Larger helical populations in peptides derived from the dimerization helix of the capsid protein of HIV-1 results in peptide binding toward regions other than the "hotspot" interface.** *Biomacromolecules* 2011, **12:**3252-3264.
44. Garzon MT, Lidon-Moya MC, Barrera FN, Prieto A, Gomez J, Mateu MG, Neira JL: **The dimerization domain of the HIV-1 capsid protein binds a capsid protein-derived peptide: a biophysical characterization.** *Protein Sci* 2004, **13:**1512-1523.
45. Zhang H, Zhao Q, Bhattacharya S, Waheed AA, Tong X, Hong A, Heck S, Curreli F, Goger M, Cowburn D, et al: **A cell-penetrating helical peptide as a potential HIV-1 inhibitor.** *J Mol Biol* 2008, **378:**565-580.
46. Zhang H, Curreli F, Zhang X, Bhattacharya S, Waheed AA, Cooper A, Cowburn D, Freed EO, Debnath AK: **Antiviral activity of alpha-helical stapled peptides designed from the HIV-1 capsid dimerization domain.** *Retrovirology* 2011, **8:**28.
47. Sticht J, Humbert M, Findlow S, Bodem J, Muller B, Dietrich U, Werner J, Krausslich HG: **A peptide inhibitor of HIV-1 assembly in vitro.** *Nat Struct Mol Biol* 2005, **12:**671-677.
48. Potash MJ, Bentsman G, Muir T, Krachmarov C, Sova P, Volsky DJ: **Peptide inhibitors of HIV-1 protease and viral infection of peripheral blood lymphocytes based on HIV-1 Vif.** *Proc Natl Acad Sci U S A* 1998, **95:**13865-13868.
49. Adekale MA, Cane PA, McCrae MA: **Changes in the Vif protein of HIV-1 associated with the development of resistance to inhibitors of viral protease.** *J Med Virol* 2005, **75:**195-201.
50. Baraz L, Friedler A, Blumenzweig I, Nussinuv O, Chen N, Steinitz M, Gilon C, Kotler M: **Human immunodeficiency virus type 1 Vif-derived peptides inhibit the viral protease and arrest virus production.** *FEBS Lett* 1998, **441:**419-426.
51. Baraz L, Hutoran M, Blumenzweig I, Katzenellenbogen M, Friedler A, Gilon C, Steinitz M, Kotler M: **Human immunodeficiency virus type 1 Vif binds the viral protease by interaction with its N-terminal region.** *J Gen Virol* 2002, **83:**2225-2230.
52. Friedler A, Blumenzweig I, Baraz L, Steinitz M, Kotler M, Gilon C: **Peptides derived from HIV-1 Vif: a non-substrate based novel type of HIV-1 protease inhibitors.** *J Mol Biol* 1999, **287:**93-101.
