# Supplementary material for: An integrated map of HIV genome-wide variation from a population perspective
Source: Retrovirology. 2015 Feb 15;12:18. doi: 10.1186/s12977-015-0148-6 (PMC4358901; doi:10.1186/s12977-015-0148-6)
Supplement: Additional file 2: Table S1. — Average amino acid diversity of HIV monomeric and multimeric proteins. Table S2. Summary of average AA diversity, average dN, average dS and average dN/dS in the HIV-1 subtype A1, B, C and CRF 01_AE genomes. Table S3. Statistical of dN/dS, dN and dS distributions in the monomeric and multimeric protein groups of the HIV-1 subtype A1, B, C and CRF01_AE genomes. Table S4. Summary of 121 peptide inhibitors derived from HIV-1 proteins. Table S5. Summary of protein structures and PDB data for HIV-1 and HIV-2 proteins. Table S6. Percentages of deletions and insertions in the HIV-1 and HIV-2 full-length genomic sequence alignments. Table S7. Summary of antibody, CD4+, CD8+ T cell epitope positions. Table S8. Cutoffs for determining solvent exposed residues. [file 12977_2015_148_MOESM2_ESM.pdf]

# Additional tables

An integrated map of HIV genome-wide variation from a population perspective

# Authors and Affiliations

Guangdi Li[1,2*], Supinya Piampongsant[2], Nuno Rodrigues Faria[3], Arnout Voet[4], Andrea-Clemencia Pineda-Peña[2,5], Ricardo Khouri[2,6], Philippe Lemey[2], Anne-Mieke Vandamme[2,7], Kristof Theys [2*]

[1] Metabolic Syndrome Research Center, the Second Xiangya Hospital, Central South University, Changsha, Hunan, China

[2] Rega Institute for Medical Research, Department of Microbiology and Immunology, KU Leuven, Leuven, Belgium

[3] Department of Zoology, University of Oxford, Oxford OX1-3PS, UK

[4] Zhang IRU, RIKEN Institute Laboratories, Hirosawa 2-1, Wako-shi, Saitama, Japan

[5] Clinical and Molecular Infectious Disease Group, Faculty of Sciences and Mathematics, Universidad del Rosario, Bogotá, Colombia

[6] LIMI-LIP, Centro de Pesquisa Gonçalo Moniz, FIOCRUZ, Salvador-Bahia, Brasil

[7] Centro de Malária e Outras Doenças Tropicais and Unidade de Microbiologia, Instituto de Higiene e Medicina Tropical, Universidade Nova de Lisboa, Lisbon, Portugal

# Abbreviations:

AA: amino acid, ASA: solvent accessible surface area, CA: capsid, CTD: C-terminal domain, gag: group-specific antigen gene, dN/dS: ratio of non-synonymous to synonymous, NTD: N-terminal domain, $K_d$: dissociation constant, MA: matrix, NC: nucleocapsid, PR: protease, RT: reverse transcriptase, IN: integrase, LTR: long terminal region, ORF: open reading frame, Vif: viral infectivity factor, Vpr: viral protein R, Tat: trans-activator of transcription, Vpu: viral protein U, Rev: regulator of virion expression, sp: signal peptide, GP120: surface glycoprotein GP120, GP41: transmembrane glycoprotein GP41, Nef: negative regulatory factor, env: envelope gene, p2: spacer peptide 2, p1: spacer peptide 1, FP: fusion peptide, NHR: N-terminal heptad repeat, CHR: C-terminal heptad repeat, MPER: membrane-proximal external region, TM: transmembrane domain.

# Tables

**Table S 1:** Average amino acid diversity of HIV-1 monomeric and multimeric proteins

| HIV-1 clade | A1 | B | C | D | F1 | G | H |
|---|---|---|---|---|---|---|---|
| Monomers | 14.6% | 14.4% | 14.4% | 14.3% | 14.1% | 14.1% | 13.9% |
| Multimers | 12.7% | 13.1% | 12.5% | 12.5% | 12.3% | 12.3% | 12.3% |
| P-value # | 5.4E-7 | 1.3E-5 | 3.8E-10 | 1.9E-11 | 5.2E-11 | 6.6E-11 | 4.9E-10 |
| | | | | | | | |
| HIV-1 clade | J | K | 01_AE | 02_AG | N | O | P |
| Monomer | 12.9% | 13.0% | 12.7% | 12.5% | 12.7% | 13.0% | 12.8% |
| Multimer | 11.3% | 11.3% | 11.1% | 11.1% | 11.1% | 11.4% | 11.1% |
| P-value | 1.1E-9 | 2.0E-9 | 6.3E-12 | 8.2E-11 | 9.1E-13 | 4.0E-14 | 8.9E-14 |

#: Mann-Whitney U-test. Monomeric proteins include: Nucleocapsid, Vpr, Vpu and p6. Multimeric proteins include: Matrix, Capsid, Protease, RT, Integrase, Vif, Tat, Rev, GP120, GP41 and Nef.

**Table S 2:** Summary of average amino acid diversity, average dN, average dS and average dN/dS in the HIV-1 subtype A1, B, C and CRF 01_AE genomes.

| | **MA** | **CA** | **NC** | **p6** | **PR** | **RT** | **IN** | **Vif** | **Vpr** | **Tat** | **Rev** | **Vpu** | **GP120** | **GP41** | **Nef** |
|---|---|---|---|---|---|---|---|---|---|---|---|---|---|---|---|
| **Subtype A1 ( n = 134* )** | | | | | | | | | | | | | | | |
| **Average AA diversity#** | 13.07% | 7.3% | 7% | 19.89% | 5.59% | 6.44% | 4.76% | 13.63% | 9.86% | 18.14% | 16.83% | 23.04% | 23.34% | 13.38% | 14.64% |
| **Average dN&** | 4.2416 | 2.2520 | 1.5649 | 5.8088 | 1.5365 | 1.6664 | 1.2301 | 3.6541 | 3.008 | 5.1568 | 5.1229 | 6.6055 | 7.9728 | 4.0475 | 4.8832 |
| **Average dS** | 7.5729 | 6.6680 | 7.6173 | 6.8687 | 5.399 | 6.6364 | 4.8250 | 4.5349 | 6.2658 | 6.9122 | 5.8713 | 8.3641 | 10.5770 | 6.1135 | 9.7136 |
| **Average dN/dS** | 0.6277 | 0.5247 | 0.2616 | 1.6407 | 0.4437 | 0.5332 | 0.4208 | 1.1979 | 0.5999 | 1.1711 | 3.7359 | 1.7621 | 1.0839 | 6.9040 | 0.6937 |
| **Subtype B ( n = 495 )** | | | | | | | | | | | | | | | |
| **Average AA diversity** | 12.92% | 4.95% | 10.83% | 14.99% | 8.21% | 6.04% | 4.91% | 14.71% | 11.29% | 19.32% | 18.34% | 20.04% | 23.93% | 15.59% | 17.79% |
| **Average dN** | 17.9835 | 7.8521 | 15.9528 | 22.7092 | 10.3275 | 8.3994 | 6.6144 | 18.6009 | 18.4932 | 31.6002 | 27.0129 | 29.8340 | 41.0006 | 22.4352 | 28.7236 |
| **Average dS** | 36.2322 | 31.6573 | 36.9683 | 30.0589 | 23.9977 | 30.0431 | 24.7345 | 29.3979 | 34.8307 | 28.6126 | 35.7222 | 40.8066 | 69.3688 | 40.0128 | 46.3504 |
| **Average dN/dS** | 0.9391 | 0.3995 | 0.6488 | 3.4335 | 0.7814 | 0.4290 | 0.7368 | 1.5756 | 1.2612 | 4.3325 | 3.9501 | 2.0519 | 0.9575 | 1.6276 | 0.9521 |
| **Subtype C ( n = 429 )** | | | | | | | | | | | | | | | |
| **Average AA diversity** | 16.11% | 5.77% | 9.39% | 15.52% | 6.15% | 5.84% | 4.45% | 10.61% | 10.64% | 15.37% | 16.10% | 20.21% | 22.89% | 14.19% | 14.96% |
| **Average dN** | 16.9613 | 6.6180 | 10.3630 | 16.9663 | 6.3052 | 6.192 | 4.5921 | 11.1515 | 11.1673 | 16.7051 | 17.6523 | 21.3779 | 26.4224 | 16.7594 | 18.2087 |
| **Average dS** | 29.8789 | 24.6275 | 32.999 | 23.3061 | 22.4909 | 24.1740 | 18.1995 | 20.2978 | 28.292 | 24.0354 | 25.3185 | 26.4743 | 34.6614 | 25.7589 | 35.6265 |
| **Average dN/dS** | 0.7263 | 0.4154 | 0.5174 | 1.5352 | 0.8951 | 0.4464 | 0.6779 | 1.5415 | 0.7458 | 2.5781 | 4.1160 | 1.9955 | 0.8544 | 7.4103 | 1.4687 |
| **CRF 01_AE ( n = 250 )** | | | | | | | | | | | | | | | |
| **Average AA diversity** | 10.06% | 3.00% | 4.92% | 13.37% | 4.34% | 3.75% | 2.39% | 9.32% | 7.97% | 13.57% | 13.34% | 12.80% | 17.40% | 9.62% | 11.78% |
| **Average dN** | 6.0259 | 1.87 | 2.5963 | 8.0067 | 2.2563 | 1.965 | 1.3935 | 5.3593 | 4.7608 | 7.4750 | 7.8306 | 7.7633 | 11.1853 | 5.6436 | 6.9714 |
| **Average dS** | 8.4715 | 7.7751 | 9.8050 | 10.9668 | 6.1963 | 7.8606 | 5.7381 | 7.4865 | 8.3815 | 7.9631 | 11.1749 | 9.7772 | 11.6962 | 8.2893 | 11.1463 |
| **Average dN/dS** | 1.2606 | 0.4052 | 0.4081 | 1.8429 | 0.4441 | 0.4436 | 0.8574 | 1.2791 | 1.08 | 2.3009 | 2.016 | 2.7341 | 1.4246 | 3.3754 | 1.0291 |

*: Number of genomic sequences used in our analysis (one sequence per patient).

#: Average AA diversity was calculated by the mean value of amino acid diversities in all codon positions of individual proteins in HIV-1 subtype A1 genome.

&: Average dN was calculated by the mean value of dN data in all codon positions of individual proteins in HIV-1 subtype A1 genome.

**Table S 3:** Statistical comparisons of dN/dS, dN and dS distributions in the monomeric and multimeric protein groups of the HIV-1 subtype A1, B, C and CRF01_AE genomes.

| Subtype /CRF | Genomic sequence number | Distribution | Median value | | P-value in Mann-Whitney U test |
|---|---|---|---|---|---|
| | | | Monomeric proteins | Multimeric proteins | |
| A1 | 134 | dN/dS | 0.2221 | 0.1436 | 0.0122 |
| | | dN | 1.4602 | 0.6674 | 0.0013 |
| | | dS | 5.7179 | 4.866 | 0.2295 |
| | | | | | |
| B | 495 | dN/dS | 0.4476 | 0.2501 | $7.7815\times 10^{-6}$ |
| | | dN | 9.1821 | 4.5409 | $1.1904\times 10^{-7}$ |
| | | dS | 25.5599 | 26.0369 | 0.6977 |
| | | | | | |
| C | 429 | dN/dS | 0.2010 | 0.1558 | 0.0208 |
| | | dN | 4.7151 | 2.3443 | 0.00086 |
| | | dS | 20.7612 | 19.0769 | 0.1013 |
| | | | | | |
| 01_AE | 250 | dN/dS | 0.2928 | 0.1478 | $9.137\times 10^{-4}$ |
| | | dN | 2.2235 | 0.8930 | $7.1285\times 10^{-6}$ |
| | | dS | 6.0276 | 5.4458 | 0.0708 |

**Table S 4:** Summary of the 121 peptide inhibitors derived from HIV-1 proteins. (1) Peptide position: HIV-1 proteins and HXB2 positions from which the peptide inhibitors were derived. The numbering of the GP41 peptide positions refers to the Env protein; (2) Peptide name: we used the peptide names as indicated in the published articles; (3) Sequence: peptide amino acid sequence, with additional molecule functional groups where relevant; (4) Target: target protein with which peptide inhibitors bind; (5) IC50/EC50/Kd: experimental outcomes of peptide performance in Kd, IC50 or EC50. The superscript 'd' indicates Kd and 'E' indicates EC50. Others without superscripts are IC50 values. GP120 inhibitors: 'n' indicates neutralizing activity, IN inhibitors: 's' indicates integrase strand transfer, '3e' indicates integrase 3'-end processing. '~' indicates approximate values; (6) HIV strain: HIV strains used for evaluation of peptide inhibitory activity. If multiple HIV-1 strains were used, subtype or group information is given in both of the columns (5) and (6). (7) Cell line: cell lines used in the experiments.

| Peptide position(1) | Peptide name(2) | Sequence(3) | Target(4) | IC$_{50}$/EC$_{50}$/Kd(5) | HIV strain(6) | Cell line(7) | Reference |
|---|---|---|---|---|---|---|---|
| GP41[638–673] | T20(enfuvirtide) | YTSLIHSLIEESQNQQEKNEQELLELDKWASLWNWF | GP41 | B:2.7±0.4nM,<br>IIIB:28nM[1],<br>BCF02>2000nM[1] | B:HXB2,<br>B:IIIB [1],<br>C: BCF02 [1] | 293T | [1],[2] |
| GP41[638–673] | T-20EK | YTSLIEELIKKSEEQQKKNEELKKLEEWAKKWNWF | GP41 | B:1.2nM | NL4-3$_{D36G}$ | MT-2 | [3] |
| GP41[621-652] | CP621-652 | QIWNNMTWMEWDREINNYTSLIHSLIEESQNQ | GP41 | HXB2:8.6±2.5nM,<br>NL4-3:5.8±0.6nM | HXB2,NL4-3 | TZM-bl | [4] |
| GP41 [621-652] | CP32M | VEWNEMTWMEWEREIENYTKLIYKILEESQEQ | GP41 | BCF02:10nM[1],<br>IIIB:5nM[1] | IIIB[1],<br>BCF02[1] | MT-2 | [1],[5] |
| GP41[628-661] | NCS-C34-Chol | WMEWK(NCS)REINNYTSLIHSLIEESQNQQEKNEQELLGSGN-Chol | GP41 | 8.4±2.2nM | HXB2, SF162,<br>CNE28,NL4-3 | HEK293T | [6] |
| GP41[528-581] | 17-70 | STMGAASMTLTVQARQLLSGIVQQQNNLLRAIEAQQHLLQLTVWGIKQLQARIL | GP41 | 391±33nM | HXB2 | TZM-bl | [7] |
| GP41[630-659] | SJ-2176 | EWDREINNYTSLIHSLIEESQNQQEKNEQEGGC | GP41 | P24-NC:101uM$^{E}$<br>CPE:142nM$^{E}$<br>Cell fusion:156nM$^{E}$ | IIIB | MT-2 | [8] |
| GP41[512-544] | IFFA | AVGIGALFLGFLGAAGSTMGARSMTLTVQARQL | GP41 | | IIIB | SupT1, TF228 | [9] |
| GP41[628-639,641-661] | ABT | WEEWDREINNYT(MPA)LIHELIEESQNQQEKNEQELL | GP41 | 1.01-66.39nM | NL4-3,subtype A,B,C | TZM-bl | [10] |
| GP41[626-663] | T1144 | TTWEAWDRAIAEYAARIEALLRALQEQQEKNEAALREL | GP41 | 0.4nM | Bal | TZM-bl | [11] |
| GP41[638-673] [626-663] | TLT35 | T20 (GGGGS)$_6$ T1144 | GP41 | IIIB:11.06±3.12nM,Bal:<br>2.24±0.68nM,Range:<br>1.83-27.87nM | IIIB,Bal,<br>Subtype:A,B,C,<br>E,F,G,O | MT-2 | [11, 12] |
| GP41[626-657] | C32-e5.0 | Ac-TTWEAWDRAIAEYAARIEALIRAAQEQQEKNC-NH$_2$ | GP41 | 6.4±1.4nM$^{d}$ | | | [13, 14] |
| GP41[626-664] | C39-e5.0 | Ac-TTWEAWDRAIAEYAARIEALIRAAQEQQEKNEAELRELC-NH2 | GP41 | 9.9±1.8nM$^{d}$ | | | [13, 14] |
| GP41[628-661] | C34 | WMEWDREINNYTSLIHSLIEESQNQQEKNEQELL | GP41 | >2uM[1] | BCF02[1] | 293T | [1],[15, 16] |
| GP41[636-661] | Aoc−βAla−P26 | Aoc−βAla−NNYTSLIHSLIEESQNQQEKNEQELL | GP41 | NL4-3D36G:130±12nM,<br>IIIB:14.9±2.99nM | NL4-3D36G,<br>IIIB | MT-2,<br>HL2/3,TZM-b | [17] |
| GP41[626-661] | MT-C34 | MTWMEWDREINNYTSLIHSLIEESQNQQEKNEQELL | GP41 | 0.5±0.1nM | NL4-3 | HL2/3 | [18] |
| GP41[626-649] | MT-SC22EK | MTWEEWDKKIEEYTKKIEELIKKS | GP41 | A:3.8-4.6nM<br>B: 1.6-9.3nM<br>C:1.3-10.8nM<br>AE:3.4-15.1nM<br>BC:0.8-6.5nM | A,B,C,A/E,B/C | TZM-bl | [19] |
| GP41[625-661] | C37 | GGHTTWMEWDREINNYTSLIHSLIEESQNQQEKNEQELLGHHHHHH | GP41 | HXB2: ~1nM<br>JR-FL:~1.5nM | HXB2,NL4-3,JR-FL, Ba-L | 293T | [20] |
| GP41[628-673] | C46(364H-3L | WMEWDREINNYTSLIHSLIEESQN | GP41 | JR-FL:23nM, | JR-FL,Bal, | U87,293T | [21] |

| | | | | | | | |
|---|---|---|---|---|---|---|---|
| | multimer) | QQEKNEQELLELDKWASLWNWF | | Bal:120nM, 117III: >100nM, HXB2:12nM | 117III,IIIB,HXB2 | | |
| GP41[628-656] | (Caca29)$_2$ | (CAcaWMEWDREINNYTSLIHSLIEESQNQQEKN)$_2$ | GP41 | 5.71nM | - | - | [22] |
| GP41[628-649] | (CAcaSC22EK)$_2$ | (CAcaWEEWDKKIEEYTKKIEELIKKS)$_2$ | GP41 | 4.9nM | - | - | [22] |
| GP41[629-662] | SC35E(SBn)$_5$H$_9$ | Ac-WEEWEKKIHEYTAKIELIKKSEEQQKKNEEELKK-NH$_2$ | GP41 | 1.02±0.33nM | ??? | TZM-bl | [23] |
| GP41[628-673] | V2o | WMTWDREIDNITQTISSAIEESQNQNEKNEQELLKLNQWDIFSNWF | GP41 | HXB2:0.42±0.15nM, BaL:0.51±0.11nM,SIVmac251:5.0±3.3nM | HXB2,BaL,SIVmac251 | 293T | [24] |
| GP41[553-590] | DP-107 | NNLLRAIEAQQHLLQLTVWGIKQLQARILAVERYLKDQ | GP41 | 2.7uM | IIIB | MT4 | [25] |
| GP41[627-662] | SFT(Sifuvirtide) | SWETWEREIENYTRQIYRILEESQEQQDRNERDLLE | GP41 | A:1.81nM, B:10.35nM, C:3.84nM[26] | Subtype A,B,C | MT-2 | [26-28] |
| GP41[559-581] | IQN23 | Ac-RMKQIEDKIEEIESKQKKIENEIARIKKL-IEAQQHLLQLTVWGIKQLQARIL-NH$_2$ | GP41 | 15±7nM | HXB2 | 293T | [29] |
| GP41[546-581] | N36 | SDIVQQQNNLLRAIEAQQHLLQLTVWGIKQLQARIL | GP41 | 180±70nM$^{E}$ | NL4-3 | 293T, MT-2 | [30] |
| GP41[546-581] | N36Fd | SDIVQQQNNLLRAIEAQQHLLQLTVWGIKQLQARIL-GYIPEAPRDGQAYVRKDGEWVLLSTFL | GP41 | NL4-3: 56.34±9.24nM,IIIB:99nM; Bal: 182.9nM,93IN101:1.21nM | IIIB,NL4-3, BaL,93IN101 | MT-2,PMBC,TZM-bl | [31] |
| GP41[559-586] | N28Fd | IEAQQHLLQLTVWGIKQLQARILAVERY-GYIPEAPRDGQAYVRKDGEWVLLSTFL | GP41 | NL4-3:26.95±0.02nM, IIIB:39nM | IIIB,NL4-3, BaL, 93IN101 | MT-2,PMBC,TZM-bl | [31] |
| GP41[536-581] | N46 | TLTVQARQLLSGIVQQQNNLLRAIEAQQHLLQLTVWGIKQLQARIL | GP41 | >1uM (IIIB) | IIIB, 92US657, 94UG103 | MT-2,H9, | [32] |
| GP41[536-581] | N46FdFc | TLTVQARQLLSGIVQQQNNLLRAIEAQQHLLQLTVW-GIKQLQARILGYIPEAPRDGQAYVRKDGEWVLLSTFL-(H)$_6$ | GP41 | 310±25nM | IIIB, 92US657, 94UG103 | MT-2,H9, | [32] |
| GP41[626-663] | T2635 | TTWEAWDRAIAEYAARIEALIRAAQEQQEKNEAALREL | GP41 | 6.24nM~393.0nM | LAI | TZM-bl | [33] |
| GP41[626-663] | T-2544 | MTWEAWDRAIAEYAARIEALIRAAQEQQEKNEAALREL | GP41 | 7nM | IIIB | PBMC, MT-2 | [34] |
| GP41[626-661] | T-651 | MTWMEWDREINNYTSLIHSLIEESQNQQEKNEQELL | GP41 | 8nM | IIIB | PBMC, MT-2 | [34] |
| GP41[628-683] | P5 | WMEWDREINNYTSLIHSLIEESQNQQEKNEQELLELDKWASLWNWFNITNWLWYIK | GP41 | ~60nM | LAI, JR-CSF | Hela,PBMC | [35] |
| | | | | | | | |
| IN[95-109] | Alpha-1 | QETAYFLLKLAGRWP-CONH$_2$ | IN | 3.5uM | | - | [36] |
| IN[171-187] | Alpha-5 | HLKTAVQMAVFIHNFKR-CONH$_2$ | IN | 3.0uM | | - | [36] |
| IN[196-209] | Alpha-6 | AGERIVDIIATDIQ-CONH$_2$ | IN | 2.0uM | | - | [36] |
| IN[95-107] | Alpha-1s | QETAYFLLKLAGR-CONH$_2$ | IN | 150uM | | - | [36] |
| IN[196-205] | Alpha-6s | AGERIVDIIA-CONH$_2$ | IN | 30uM | | - | [36] |
| IN[82-89] | Beta-3 | GYIEAEVI-CONH2 | IN | >1mM | | - | [36] |
| IN[95-109] | H104 | QETAYFLLKLALRWP-CONH2 | IN | - | - | - | [37] |
| IN[97-108] | NL-6 | TAYFLLKLAGRW | IN | 2.7uM$^{s}$, 21 uM$^{3E}$ | - | - | [38] |
| IN[99-104] | NL6-5 | YFLLKL | IN | 20uM$^{s,\ 3E}$ | - | - | [38] |
| IN[129-139] | NL-9 | ACWWAGIKQEF | IN | 56uM$^{s}$, 95uM$^{3E}$ | - | - | [38] |
| IN[173-188] | INS K188E | WTAVQMAVFIHNFKRE | IN | 5.2±0.2uM | HXB2 | HEK293T | [39] |
| IN[92-108] | INH1 | ATGQETAYFLLKLAGKA-CONH$_2$ | IN | 150uM$^{s}$, 250uM$^{3E}$ | NL4 –3 | CEM-12D7 | [40] |
| IN[167-187] | INH5 | DQAEHLKTAVQMAVFIHNYKA-CONH$_2$ | IN | 4.7uM$^{s}$, 11uM$^{3E}$ | | | [40] |
| IN[147-176] | K159 | SQGVVESMNKELKKIIGQVRDQAEHLKTAY | IN | 16nM$^{S}$,16nM$^{3E}$ | HXB2D | - | [41],[42],[43] |

| | | | | | | | |
|---|---|---|---|---|---|---|---|
| IN[151-176] | EAA26 | VESMNEELKKIIAQVRAQAEHLK TAY | IN | - | - | - | [42],[44] |
| IN[171-187,196-209] | a5-Cmpi-a6 | HLKTAVQMAVFIHNFKR-Cmpi-AGERIVDIIATDIQ-NH2 | IN | 460±30nM | | | [45] |
| | | | | | | | |
| CA[175-194] | CAC1 | Ac-EQASQEVKNWMTETLLVQNA-CONH2 | CA | 50uM$^{d}$ | BH10 | - | [46] |
| CA[207-217] | Capsid1 | PAATLEEMMTA | CA | | | H9 | [47] |
| CA[175-193] | CAC1M | SESAASSVKAWMTETLLVANTSS | CA | 8±1uM$^{d}$ | HXB2 | U87-CD4-CXCR4 | [48] |
| CA[175-194] | CAC1C | ESASSSVKAWMTETLLVQNA | CA | 19±8uM$^{d}$ | HXB2 | U87-CD4-CXCR4 | [48] |
| CA[178-192] | NYAD-201 | AQEVKXWMTXTLLVA (X= (S)-2-alpha- (2'-pentenyl)alanine) | CA | IIIB: 4.29±0.62uM, MN: 3.03±0.61uM, SF2: 5.06±1.37uM, RF: 2.84±0.63uM, Bal: 4.73±1.92uM, 89.6: 5.21±0.87uM | IIIB,MN,RF,SF 2,BaL,89.6 | MT-2,PBMC | [49] |
| CA[178-192] | NYAD-202 | AQAVKXWMTXTLLVA (X= (S)-alpha- (2'-pentenyl)alanine) | CA | IIIB: 2.36±0.33uM, MN: 2.47±0.71uM, SF2: 4.48±0.84uM, RF: 2.64±0.39uM, Bal: 2.23±0.44uM, 89.6: 3.471±0.22uM | IIIB,MN,RF,SF 2,BaL,89.6 | MT-2,PBMC | [49] |
| CA[181-192] | P-1 | VKNWMTETLLRQ | CA | 3.8±3.5uM$^{d}$ | BH10 | - | [50] |
| CA[124-133] | peptide 1 | IPVGEIYKRW | CA | 37±10uM$^{d}$ | | | [51] |
| | | | | | | | |
| RT[285-301] | $P_{AW}$ | GTKWLTEWIPLTAEAEC | RT | 700±200nM$^{d}$ | LAI | PBMC | [52] |
| RT[285-299] | P27 | GTKWLTEWIPLTAEC | RT | 50±10nM$^{d}$ | LAI | PBMC | [52] |
| RT[285-296] | P24 | GTKWLTEWIPLC | RT | 700±50nM$^{d}$ | LAI | PBMC | [52] |
| RT[395-404] | Pep-7 | KETWETWWTE | RT | 138nM$^{d}$ | BH10 | - | [53],[54] |
| RT[389-407] | Peptide1 | FKLPIQKETWETWWTEYWE | RT | 1.2uM$^{d}$ | LAV | MT-4 | [55] |
| | | | | | | | |
| PR [83-93] | p-$S_8$ | NIIGRNLLTQI | PR | 2.58±0.78uM[56] | - | - | [56],[57],[58, 59] |
| PR[1-5,95-99] | PF1 | PQITL-$(G)_3$-CTLNF | PR | 40uM(HIV1),20uM(HIV2) | HIV1,HIV2 | | [60] |
| | | | | | | | |
| MA[71-87] | 8L | $CH_3CO$-GSEELRSLYNTIAVLGC-$NH_2$ | MA | NL4-3: 2.3±0.3uM$^{E}$ JR-CSF: 7.8uM | NL4-3, JR-CSF | MT-4,PM1/CCR5 | [61] |
| MA[81-97] | 9L | $CH_3CO$-TIAVLYSVHQRIDVKGC-$NH_2$ | MA | NL4-3: 2.1±0.5uM$^{E}$ JR-CSF: 0.58uM | NL4-3, JR-CSF | MT-4,PM1/CCR5 | [61] |
| MA[47-59] | 4/5m | NPGLLETSEGCRQ | MA | 615ug/ml | IIIB | H9 | [47] |
| | | | | | | | |
| RT [166-185] | 4286 | KILEPFRKQNPDIVIYQYMD | IN | 4.8uM$^{3E}$,4.5uM$^{S}$ | BH10 | - | [62] |
| RT [516-535] | 4321 | ELVNQIIEQLIKKEKVYLAW | IN | 6.9uM$^{3E}$,5uM$^{S}$ | BH10 | - | [62] |
| RT [176-195] | 34 | PDIVIYQYMDDLYVGSDLEI | IN | 10uM$^{S}$, 6uM$^{3E}$ | HXB2 | - | |
| RT [366-385] | 53 | KQLTEAVQKITTESIVIWGK | IN | 7±1uM$^{3E}$,4±1uM$^{S}$ | HXB2 | - | [63] |
| RT [396-415] | 56 | ETWETWWTEYWQATWIPEWE | IN | 6±1uM$^{3E}$,2±1uM$^{S}$ | HXB2 | - | [63] |
| RT [486-505] | 65 | LQDSGLEVNIVTDSQYALGI | IN | 2uM$^{S}$, 11uM$^{3E}$ | HXB2 | - | [63] |
| RT [526-545] | 64 | ELVNQIIEQLIKKEKVYLAW | IN | 14uM$^{S}$, 15uM$^{3E}$ | HXB2 | - | [63] |
| IN[46-65] | #4330 | KGEAMHGQVDCSPGIWQLDC | RT | 4.2±0.2uM$^{RDDP}$,6.8±0.7uM$^{DDDP}$ | HXB2R | - | [64] |
| | | | | | | | |
| Vpr [33-47] | Vpr 33-47 | HFPRIWLHSLGQHIY | IN | 41uM$^{s}$, 187uM$^{3e}$ | BH10 | - | [65] |
| Vpr [53-67] | Vpr 53-67 | TWAGVEAIIRILQQL | IN | 144uM$^{s}$, >200uM$^{3e}$ | BH10 | - | [65] |
| Vpr [57–71] | Vpr 57-71 | VEAIIRILQQLLFIH | RT/IN | 0.22uM | BH10 | - | [65] |
| Vpr [61-75] | Vpr 61-75 | IRILQQLLFIHFRIG | RT/IN | 0.7uM$^{rddp}$, 1.3uM$^{dddp}$ | BH10 | - | [65] |
| Vpr[55-69] | Vpr-1 | AGVEAIIRILQQLLF | IN | - | HXB2, JR-CSF | MT-4 | [66] |
| Vpr[64-75] | Vpr-3 R8 | Ac-LQQLLFIHFRIG-RRRRRRRR-$NH_2$ | IN | 4±0.1nM$^{s}$[66], 8±1nM$^{3e}$[66], | HXB2 [66],[67], JR- | MT-4 | [66],[67] |

| | | | | | | | |
|---|---|---|---|---|---|---|---|
| | | | | 60±10nM$^{s}$[67], 130±20nM$^{3e}$[67] | CSF[66] | | |
| Vpr[58-75] | Vpr-4 R8 | Ac-EAIIRILQQLLFIHFRIG-RRRRRRRR-NH$_2$ | IN | 5±2nM$^{s}$, [66] 6±6 nM$^{3e}$[66] 40±10nM$^{s}$[67] 90±10nM$^{3e}$[67] | HXB2[66],[67], JR-CSF[66] | MT-4[66],[67] | [66],[67] |
| Vpr [65-79] | Vpr 65-79 | QQLLFIHFRIGCQHS | IN | 14uM$^{s}$, 76uM$^{3e}$ | BH10 | - | [65] |
| Vpr[58-75] | Vpr-15 | Ac-EAEIRIKQQLLFIHFRIG-RRRRRRRR-NH$_2$ | IN | 31±10nM$^{S}$, 40±1nM$^{3E}$ | HXB2, JR-CSF | MT-4 | [66] |
| | | | | | | | |
| Vif[30-65] | Peptide4 | YVSGKARGWFYRHHYESPHPRISSEVHIPLGDARLV | PR | 230-250uM | IIIB | Hut 78 | [68] |
| Vif[78-92] | Peptide6 | DWHLGQGVSIEWRKK | PR | 110uM | IIIB | Hut 78 | [68] |
| Vif[88-98] | Peptide7 | EWRKKRYSTQV | PR | 25uM[68], 3.31uM[69] | IIIB | Hut 78 | [68], [69] |
| Vif[41-65] | Vif41-65 | RHHYESPHPRISSEVHIPLGDARLV | PR/IN | | HXB-2 | PBL | [70] |
| | | | | | | | |
| p6* [1-8] | TFP | FLREDLAF | PR | 98±10uM$^{I}$ | HXB2 | | [71] |
| p6* [4-6] | - | EDL | PR | 50±9uM$^{I}$ | HXB2 | | [71] |
| PR[1-5]Tat [49-61]p6*[53-56]PR[95-99] | P27 | PQITL-RKKRRQRRRPPQV- SFNF-CTLNF | PR | 0.23-0.32uM/5uM | A01 patient, LAI[72] | MT-2,H9 | [72], [73] |
| | | | | | | | |
| Rev [1-30] | Rev1-30 | MAGRSGDSDEELLKTVRLIKFLYQSNPPPS | IN | 6.5±0.2uM$^{d}$ | HXB2 | - | [74] |
| Rev [13-23] | Rev13-23 | LKTVRLIKFLY | IN | 2.8±0.1uM$^{d}$ | HXB2 | HeLa | [74],[75] |
| Rev [49-74] | Rev49-74 | QRQIRSISGWILSTYLGRPAEPVPLQ | IN | 11.2±0.5uM$^{d}$ | HXB2 | - | [74] |
| Rev [53-67] | Rev53-67 | RSISGWILSTYLGRP | IN | 6.9±0.1uM$^{d}$ | HXB2 | HeLa | [74],[75] |
| CA[229-231]p2[1-3] | 6a | RVL-FEA-Nle | PR | NL4-3: 2.60±0.4nM, MDR769: 4.40±0.7nM | NL4-3, MDR769 | - | [76] |
| | | | | | | | |
| GP120[280-302] | NTM | RSANFTDNAKTIIVQLNQSVEIN | CD4 receptor | | BH-10 | - | [77] |
| GP120[424-433] | Peptide 1 | INMWQEVGKA | CD4 | 28uM | IIIB | - | [78] |
| GP120[365-373] | Peptide 2 | SGGDPEIVT | CD4 | 6uM | IIIB | - | [78] |
| GP120[312-317] | SPC3 | [GPGRAF]8-K$_4$-K$_2$-K-βA | chemokine receptors α/β | 7.7±0.4uM | LAI | Xenopus oocyte | [79] |
| GP120[293-334] | V3-BH10 | EINCTRPNNNTRKSIRIQRGPGRAFVTIGKIGNMRQAHCNIS | IgG, MAbs,CD19 | - | IIIB,MN | MT-4, PMBC | [80] |
| GP120[290-334] | V3-89.6 | ESVVINCTRPNNNTRRRLSIGPGRAFYARRNIIGDIRQAHCNIS | IgG, MAbs,CD19 | - | IIIB,MN | MT-4, PMBC | [80] |
| GP120[290-320,323-334] | V3-ELI | ESVKITCARPYQNTRQRTPIGLGQSLYTTRSRSIIGQAHCNIS | IgG, MAbs,CD19 | - | IIIB,MN | MT-4, PMBC | [80] |
| GP120[298-321] | 62.19 | RPNNNTRKRIRIQRGPGRAFVAIE | F39F,447-52D Fab | 31%$^{n}$ | IIIB,89.6 | MT-2 | [81] |
| GP120[296-313,315-331] | V3$_B$-FP | CTRPNNNTRKSIRIGPGQTFYATGDIIGDIRQAHC | | >50%$^{n}$ | BZ167, DJ263, NL43 | | [82] |
| GP120[421-436,298-321] | C4–V3 T303C–E322C | KQIINMWQEVGKAMYA-RPNNNCRKSIHIGPGRAFYTTGCG | chemokine receptors | - | IIIB,NL4-3, JRFL | 293T,Rabbit | [83] |
| GP120[326-340] | 15K | IRKAHCNISRAKWND | CXCR4,CCR5 | | | 293T,PBMC,MDM | [84] |
| GP120[326-340] | 15D | IRKAHCNISRADWND | CXCR4,CCR5 | | | 293T,PBMC,MDM | [84] |
| GP120[157-171] | CT319 | CSFNITTEIRDKVKK | Tat | | HXB2 | U937 | [85] |
| | | | | | | | |
| IN[170-191], IN[214-228], IN[259-273] | CCD$_{170-191}$,CTD$_{214-228}$,CTD$_{259-274}$ | (1)EHLKTAVQMAVFIHNFKRKGGI,(2)QKQITKIQNFRVYYR (3)VVPRRKVKIIRDYGK | Transportin-SR2 | - | - | - | [86] |
| IN[161-174] | NLS(IN) | IIGQVRDQAEHLKC-NH2 | Importin-alpha | | - | | [87] |
| | | | | | | | |
| Tat[48-57] | R10 | RRRRRRRRRR | | 50uM$^{e}$ | IIIB | MAGI | [88] |
| Tat[49-58] | Tat11 | RKKRRGRRRC-NH2 | | 5nM$^{d}$ | | Colo-205 | [89] |
| Tat[11-50] | - | WKHPGSQPKTACTNCYCKKCCFHCQVCFITKALGISYGRK | CXCR4 | | NL4-3 | 293T | [90, 91] |

| | | | | | | | |
|---|---|---|---|---|---|---|---|
| | | | | | | | |
| Rev[34-47] | Rev 8 | Ac-RRRRERQRKRRRRR-OH | RRE | ~150nM | - | - | [92] |
| | | | | | | | |
| MA[11-47] | p17(11-47) | GELDRWEKIRLRPGGKKKYKLKH IVWASRELERFAVN | Ca2+/CaM | - | - | - | [30] |
| | | | | | | | |
| GP41[577-586] | | QARVLAVERY | IgA | | IIIB,ADA | TZM-bl | [93] |
| GP41[628–683] | Peptide P5 | WMEWDREINNYTSLIHSLIEESQN QQEKNEQELLELDKWASLWNWF NITNWLWYIK | GP41-Antibody | 61±1.5uM | HXB2 | Hela-CD4-LTR-LacZ | [35] |

**Table S 5:** Summary of protein structures and PDB data for HIV-1 and HIV-2 proteins

| Gene | | ***gag*** | | | | | | ***pol*** | | | ***vif*** | ***vpr*** | ***tat*** | ***rev*** | ***vpu/vpx**** | ***env*** | | ***nef*** |
|---|---|---|---|---|---|---|---|---|---|---|---|---|---|---|---|---|---|---|
| Protein | | Matrix | Capsid | p2 | Nucleocapsid | p1 | p6 | Protease | RT | Integrase | Vif | Vpr | Tat | Rev | Vpu/Vpx | GP120 | GP41 | Nef |
| Number of units | | 3 | 5,6 | 1 | 1 | 1 | 1 | 2 | 2 | 4 | 2,3,4 | 1 | 2 | 2,6 | 1 | 3 | 3 | 2 |
| HIV-1 | AA length | 132 | 231 | 14 | 55 | 16 | 52 | 99 | 560 | 288 | 192 | 96 | 101 | 116 | 82 | 481 | 345 | 206 |
| | Multimer | 1HIW # | 3H4E | - | - | - | - | 1A30 | 1N6Q | 1K6Y | - | - | - | 3LPH | - | 4NCO | 2XRA | - |
| | Monomer | - | - | 1U57 | 1A1T | - | 2C55 | - | - | - | 4N9F | 1M8L | 1K5K | - | 1VPU | - | - | 4EMZ |
| HIV-2 | AA length | 135 | 229 | 17 | 52 | 14 | 64 | 99 | 559 | 292 | 215 | 102 | 130 | 107 | 113 | 503 | 354 | 263 |
| | Multimer | - | 2WLV | - | - | - | - | 3S45 | 1MU2 | - | - | - | - | - | - | - | - | - |
| | Monomer | 2K4E | - | - | 2E1X | - | - | - | - | 3F9K | - | - | - | - | - | - | - | - |

#: PDB code from the RCSB Protein Data Bank;

*: Vpu in HIV-1 and Vpx in HIV-2;

-: either the data is not available or do not exist. For HIV multerimeric proteins, structures with different units can coexist such as pentamers and hexamers of Capsid [94], dimers and hexamers of Rev [95] and dimers, trimers and tetramers of Vif [96].

HIV-1 reference: HXB2, HIV-2 reference: BEN

**Table S 6:** Percentages of deletions and insertions in the HIV-1 and HIV-2 full-length genomic sequence alignments

| Type | **HIV-1** | | | | | | | | | | | | | | **HIV-2** | |
|---|---|---|---|---|---|---|---|---|---|---|---|---|---|---|---|---|
| Group | M | | | | | | | | | | | N | O | P | A | B |
| Subtype/CRF | A1 | B | C | D | F1 | G | H | J | K | 01_AE | 02_AG | | | | | |
| Percentage of NT deletions in MSA | 1.0% | 0.44% | 0.34% | 0.39% | 0.94% | 0.35% | 0.07% | - | 0.09% | 0.56% | 1.32% | 1.31% | 0.41% | 1.03% | 0.41% | - |
| Percentage of NT insertions in MSA | - | 0.04% | 0.06% | - | 0.17% | 0.04% | - | - | 0.33% | 0.01% | - | 0.08% | 0.23% | 0.05% | 0.08% | 0.01% |
| Percentage of NT indels # in MSA | 1.0% | 0.48% | 0.40% | 0.39% | 1.11% | 0.39% | 0.07% | - | 0.42% | 0.57% | 1.32% | 1.39% | 0.64% | 1.08% | 0.49% | 0.01% |

*: Only the HIV coding regions are counted.

-: The percentage is less than 0.01%.

#: Deletions and insertions.

**Table S 7:** Summary of antibody, CD4+ and CD8+ T cell epitope positions

| | Antibody epitope positions | CD4+ T cell epitope positions | CD8+ T cell epitope positions |
|---|---|---|---|
| **Matrix** | 20-31 | 1-107,118-132 | 11-44,74-101,124-132 |
| **Capsid** | 64-75 | 1-219 | 3-56,61-92,94-104,108-117,121-153,161-189,197-205, 217-231 |
| **p2** | | 2-14 | 1-10 |
| **NC** | | 1-55 | 28-36,50-55 |
| **p1** | | 1-16 | 1-10 |
| **p6** | | 1-43 | 33-41 |
| **Protease** | | 53-70 | 3-11,30-42,57-66,68-90 |
| **RT** | 249-263,295-304,521-531 | 36-53,97-111,156-181,195-209,249-272,276-317,338-352,384-398,411-443 | 18-26,33-43,73-82,93-101,107-115,118-135,137-166,173-187,202-210,244-252,260-279,293-301,309-318,333-350,356-366,375-383,392-401,436-457,495-505,520-528 |
| **Integrase** | | 16-30,79-93,171-234,242-267 | 28-36,66-74,78-93,114-121,123-132,135-143,165-194,197-211,219-227,260-271 |
| **Vif** | | 65-76,81-96 | 17-26,28-39,48-66,79-89,102-111,158-168 |
| **Vpr** | | 32-96 | 29-42,48-67 |
| **Tat** | 47-60 | 17-55,64-80 | 30-49 |
| **Rev** | | 9-56 | 14-23,57-75 |
| **Vpu** | | 19-34 | 5-13,29-37 |
| **GP120** | 101-121,131-166,182-191,206-215,269-292,390-413,424-444,468-481 | 1-34,41-55,57-103,125-159,164-237,239-276,278-327,333-481 | 1-39,48-56,74-82,169-177,179-196,268-277,280-300,345-353,386-397 |
| **GP41** | 14-32,50-104,128-172 | 36-77,82-186,189-206,221-233,303-345 | 46-54,66-82,95-103,187-201,259-291,294-311,320-327,332-345 |
| **Nef** | 90-98 | 3-59,64-102,104-128,140-154,162-206 | 13-27,37-45,68-100,105-145,180-191 |

**Table S 8:** Cutoffs for determining solvent exposed residues

| **Amino acid** | A | R | N | D | C | Q | E | G | H | I |
|---|---|---|---|---|---|---|---|---|---|---|
| **ASA(Å²)** | 39.91 # | 62.79 | 52.71 | 50.64 | 34.63 | 63.68 | 59.14 | 55.65 | 43.91 | 48.39 |
| **Amino acid** | L | K | M | F | P | S | T | W | Y | V |
| **ASA(Å²)** | 55.76 | 52.23 | 63.85 | 50.23 | 45.29 | 37.80 | 46.01 | 76.61 | 71.12 | 47.58 |

#: For each amino acid, the cutoff is calculated using the 25% of the maximum ASA in all HIV-1 proteins, as described in [97].

# References


1. He Y, Cheng J, Lu H, Li J, Hu J, Qi Z, Liu Z, Jiang S, Dai Q: **Potent HIV fusion inhibitors against Enfuvirtide-resistant HIV-1 strains.** *Proc Natl Acad Sci U S A* 2008, **105:**16332-16337.
2. Champagne K, Shishido A, Root MJ: **Interactions of HIV-1 inhibitory peptide T20 with the gp41 N-HR coiled coil.** *J Biol Chem* 2009, **284:**3619-3627.
3. Shimane K, Kawaji K, Miyamoto F, Oishi S, Watanabe K, Sakagami Y, Fujii N, Shimura K, Matsuoka M, Kaku M, et al: **HIV-1 resistance mechanism to an electrostatically constrained peptide fusion inhibitor that is active against T-20-resistant strains.** *Antimicrob Agents Chemother* 2013.
4. Chong H, Yao X, Qiu Z, Qin B, Han R, Waltersperger S, Wang M, Cui S, He Y: **Discovery of critical residues for viral entry and inhibition through structural Insight of HIV-1 fusion inhibitor CP621-652.** *J Biol Chem* 2012, **287:**20281-20289.
5. Yao X, Chong H, Zhang C, Qiu Z, Qin B, Han R, Waltersperger S, Wang M, He Y, Cui S: **Structural Basis of Potent and Broad HIV-1 Fusion Inhibitor CP32M.** *J Biol Chem* 2012, **287:**26618-26629.
6. Zhao L, Tong P, Chen YX, Hu ZW, Wang K, Zhang YN, Zhao DS, Cai LF, Liu KL, Zhao YF, Li YM: **A multi-functional peptide as an HIV-1 entry inhibitor based on self-concentration, recognition, and covalent attachment.** *Org Biomol Chem* 2012, **10:**6512-6520.
7. Sackett K, Wexler-Cohen Y, Shai Y: **Characterization of the HIV N-terminal fusion peptide-containing region in context of key gp41 fusion conformations.** *J Biol Chem* 2006, **281:**21755-21762.
8. Jiang S, Lin K, Strick N, Neurath AR: **HIV-1 inhibition by a peptide.** *Nature* 1993, **365:**113.
9. Gerber D, Pritsker M, Gunther-Ausborn S, Johnson B, Blumenthal R, Shai Y: **Inhibition of HIV-1 envelope glycoprotein-mediated cell fusion by a DL-amino acid-containing fusion peptide: possible recognition of the fusion complex.** *J Biol Chem* 2004, **279:**48224-48230.
10. Chong H, Yao X, Zhang C, Cai L, Cui S, Wang Y, He Y: **Biophysical property and broad anti-HIV activity of albuvirtide, a 3-maleimimidopropionic acid-modified peptide fusion inhibitor.** *PLoS One* 2012, **7:**e32599.
11. Cai L, Pan C, Xu L, Shui Y, Liu K, Jiang S: **Interactions between different generation HIV-1 fusion inhibitors and the putative mechanism underlying the synergistic anti-HIV-1 effect resulting from their combination.** *FASEB J* 2012, **26:**1018-1026.
12. Pan C, Cai L, Lu H, Lu L, Jiang S: **A novel chimeric protein-based HIV-1 fusion inhibitor targeting gp41 glycoprotein with high potency and stability.** *J Biol Chem* 2011, **286:**28425-28434.

13. Cai L, Balogh E, Gochin M: **Stable extended human immunodeficiency virus type 1 gp41 coiled coil as an effective target in an assay for high-affinity fusion inhibitors.** *Antimicrob Agents Chemother* 2009, **53:**2444-2449.
14. Gochin M: **A Suite of Modular Fluorescence Assays Interrogate the Human Immunodeficiency Virus Glycoprotein-41 Coiled Coil and Assist in Determining Binding Mechanism of Low Molecular Weight Fusion Inhibitors.** *Assay Drug Dev Technol* 2012.
15. Lu M, Blacklow SC, Kim PS: **A trimeric structural domain of the HIV-1 transmembrane glycoprotein.** *Nat Struct Biol* 1995, **2:**1075-1082.
16. Hollmann A, Matos PM, Augusto MT, Castanho MA, Santos NC: **Conjugation of cholesterol to HIV-1 fusion inhibitor C34 increases peptide-membrane interactions potentiating its action.** *PLoS One* 2013, **8:**e60302.
17. Wang C, Shi W, Cai L, Lu L, Wang Q, Zhang T, Li J, Zhang Z, Wang K, Xu L, et al: **Design, synthesis, and biological evaluation of highly potent small molecule-peptide conjugates as new HIV-1 fusion inhibitors.** *J Med Chem* 2013, **56:**2527-2539.
18. Chong H, Yao X, Sun J, Qiu Z, Zhang M, Waltersperger S, Wang M, Cui S, He Y: **The M-T hook structure is critical for design of HIV-1 fusion inhibitors.** *J Biol Chem* 2012, **287:**34558-34568.
19. Chong H, Yao X, Qiu Z, Sun J, Zhang M, Waltersperger S, Wang M, Liu SL, Cui S, He Y: **Short-peptide fusion inhibitors with high potency against wild-type and enfuvirtide-resistant HIV-1.** *FASEB J* 2013, **27:**1203-1213.
20. Kahle KM, Steger HK, Root MJ: **Asymmetric deactivation of HIV-1 gp41 following fusion inhibitor binding.** *PLoS Pathog* 2009, **5:**e1000674.
21. Dervillez X, Huther A, Schuhmacher J, Griesinger C, Cohen JH, von Laer D, Dietrich U: **Stable expression of soluble therapeutic peptides in eukaryotic cells by multimerisation: application to the HIV-1 fusion inhibitory peptide C46.** *ChemMedChem* 2006, **1:**330-339.
22. Ling Y, Xue H, Jiang X, Cai L, Liu K: **Increase of anti-HIV activity of C-peptide fusion inhibitors using a bivalent drug design approach.** *Bioorg Med Chem Lett* 2013, **23:**4770-4773.
23. Bai Y, Xue H, Wang K, Cai L, Qiu J, Bi S, Lai L, Cheng M, Liu S, Liu K: **Covalent fusion inhibitors targeting HIV-1 gp41 deep pocket.** *Amino Acids* 2013, **44:**701-713.
24. Brauer F, Schmidt K, Zahn RC, Richter C, Radeke HH, Schmitz JE, von Laer D, Egerer L: **A rationally engineered anti-HIV peptide fusion inhibitor with greatly reduced immunogenicity.** *Antimicrob Agents Chemother* 2013, **57:**679-688.
25. Kazmierski WM, Hazen RJ, Aulabaugh A, StClair MH: **Inhibitors of human immunodeficiency virus type 1 derived from gp41 transmembrane protein: structure--activity studies.** *J Med Chem* 1996, **39:**2681-2689.
26. Yao X, Chong H, Zhang C, Waltersperger S, Wang M, Cui S, He Y: **Broad antiviral activity and crystal structure of HIV-1 fusion inhibitor sifuvirtide.** *J Biol Chem* 2012, **287:**6788-6796.

27. He Y, Xiao Y, Song H, Liang Q, Ju D, Chen X, Lu H, Jing W, Jiang S, Zhang L: **Design and evaluation of sifuvirtide, a novel HIV-1 fusion inhibitor.** *J Biol Chem* 2008, **283:**11126-11134.
28. Wang RR, Yang LM, Wang YH, Pang W, Tam SC, Tien P, Zheng YT: **Sifuvirtide, a potent HIV fusion inhibitor peptide.** *Biochem Biophys Res Commun* 2009, **382:**540-544.
29. Eckert DM, Kim PS: **Design of potent inhibitors of HIV-1 entry from the gp41 N-peptide region.** *Proc Natl Acad Sci U S A* 2001, **98:**11187-11192.
30. Nishikawa H, Nakamura S, Kodama E, Ito S, Kajiwara K, Izumi K, Sakagami Y, Oishi S, Ohkubo T, Kobayashi Y, et al: **Electrostatically constrained alpha-helical peptide inhibits replication of HIV-1 resistant to enfuvirtide.** *Int J Biochem Cell Biol* 2009, **41:**891-899.
31. Chen X, Lu L, Qi Z, Lu H, Wang J, Yu X, Chen Y, Jiang S: **Novel recombinant engineered gp41 N-terminal heptad repeat trimers and their potential as anti-HIV-1 therapeutics or microbicides.** *J Biol Chem* 2010, **285:**25506-25515.
32. Qi Z, Pan C, Lu H, Shui Y, Li L, Li X, Xu X, Liu S, Jiang S: **A recombinant mimetics of the HIV-1 gp41 prehairpin fusion intermediate fused with human IgG Fc fragment elicits neutralizing antibody response in the vaccinated mice.** *Biochem Biophys Res Commun* 2010, **398:**506-512.
33. Eggink D, Bontjer I, Langedijk JP, Berkhout B, Sanders RW: **Resistance of human immunodeficiency virus type 1 to a third-generation fusion inhibitor requires multiple mutations in gp41 and is accompanied by a dramatic loss of gp41 function.** *J Virol* 2011, **85:**10785-10797.
34. Dwyer JJ, Wilson KL, Davison DK, Freel SA, Seedorff JE, Wring SA, Tvermoes NA, Matthews TJ, Greenberg ML, Delmedico MK: **Design of helical, oligomeric HIV-1 fusion inhibitor peptides with potent activity against enfuvirtide-resistant virus.** *Proc Natl Acad Sci U S A* 2007, **104:**12772-12777.
35. Yu H, Tudor D, Alfsen A, Labrosse B, Clavel F, Bomsel M: **Peptide P5 (residues 628-683), comprising the entire membrane proximal region of HIV-1 gp41 and its calcium-binding site, is a potent inhibitor of HIV-1 infection.** *Retrovirology* 2008, **5:**93.
36. Zhao L, O'Reilly MK, Shultz MD, Chmielewski J: **Interfacial peptide inhibitors of HIV-1 integrase activity and dimerization.** *Bioorg Med Chem Lett* 2003, **13:**1175-1177.
37. Kong R, Wang C, Ma X, Liu J, Chen W: **Peptides design based on the interfacial helix of integrase dimer.** *Conf Proc IEEE Eng Med Biol Soc* 2005, **5:**4743-4746.
38. Li HY, Zawahir Z, Song LD, Long YQ, Neamati N: **Sequence-based design and discovery of peptide inhibitors of HIV-1 integrase: insight into the binding mode of the enzyme.** *J Med Chem* 2006, **49:**4477-4486.
39. Levin A, Hayouka Z, Helfer M, Brack-Werner R, Friedler A, Loyter A: **Stimulation of the HIV-1 integrase enzymatic activity and cDNA integration by a peptide derived from the integrase protein.** *Biopolymers* 2010, **93:**740-751.

40. Maroun RG, Gayet S, Benleulmi MS, Porumb H, Zargarian L, Merad H, Leh H, Mouscadet JF, Troalen F, Fermandjian S: **Peptide inhibitors of HIV-1 integrase dissociate the enzyme oligomers.** *Biochemistry* 2001, **40:**13840-13848.
41. Sourgen F, Maroun RG, Frere V, Bouziane M, Auclair C, Troalen F, Fermandjian S: **A synthetic peptide from the human immunodeficiency virus type-1 integrase exhibits coiled-coil properties and interferes with the in vitro integration activity of the enzyme. Correlated biochemical and spectroscopic results.** *Eur J Biochem* 1996, **240:**765-773.
42. Maroun RG, Krebs D, El Antri S, Deroussent A, Lescot E, Troalen F, Porumb H, Goldberg ME, Fermandjian S: **Self-association and domains of interactions of an amphipathic helix peptide inhibitor of HIV-1 integrase assessed by analytical ultracentrifugation and NMR experiments in trifluoroethanol/H(2)O mixtures.** *J Biol Chem* 1999, **274:**34174-34185.
43. Azzi S, Parissi V, Maroun RG, Eid P, Mauffret O, Fermandjian S: **The HIV-1 integrase alpha4-helix involved in LTR-DNA recognition is also a highly antigenic peptide element.** *PLoS One* 2010, **5:**e16001.
44. Krebs D, Maroun RG, Sourgen F, Troalen F, Davoust D, Fermandjian S: **Helical and coiled-coil-forming properties of peptides derived from and inhibiting human immunodeficiency virus type 1 integrase assessed by 1H-NMR--use of NH temperature coefficients to probe coiled-coil structures.** *Eur J Biochem* 1998, **253:**236-244.
45. Zhao L, Chmielewski J: **Inhibition of HIV-1 integrase dimerization and activity with crosslinked interfacial peptides.** *Bioorg Med Chem* 2012.
46. Garzon MT, Lidon-Moya MC, Barrera FN, Prieto A, Gomez J, Mateu MG, Neira JL: **The dimerization domain of the HIV-1 capsid protein binds a capsid protein-derived peptide: a biophysical characterization.** *Protein Sci* 2004, **13:**1512-1523.
47. Niedrig M, Gelderblom HR, Pauli G, Marz J, Bickhard H, Wolf H, Modrow S: **Inhibition of infectious human immunodeficiency virus type 1 particle formation by Gag protein-derived peptides.** *J Gen Virol* 1994, **75 ( Pt 6):**1469-1474.
48. Bocanegra R, Nevot M, Domenech R, Lopez I, Abian O, Rodriguez-Huete A, Cavasotto CN, Velazquez-Campoy A, Gomez J, Martinez MA, et al: **Rationally designed interfacial peptides are efficient in vitro inhibitors of HIV-1 capsid assembly with antiviral activity.** *PLoS One* 2011, **6:**e23877.
49. Zhang H, Curreli F, Zhang X, Bhattacharya S, Waheed AA, Cooper A, Cowburn D, Freed EO, Debnath AK: **Antiviral activity of alpha-helical stapled peptides designed from the HIV-1 capsid dimerization domain.** *Retrovirology* 2011, **8:**28.
50. Domenech R, Bocanegra R, Gonzalez-Muniz R, Gomez J, Mateu MG, Neira JL: **Larger helical populations in peptides derived from the dimerization helix of the capsid protein of HIV-1 results in peptide binding toward regions other than the "hotspot" interface.** *Biomacromolecules* 2011, **12:**3252-3264.
51. Hilpert K, Behlke J, Scholz C, Misselwitz R, Schneider-Mergener J, Hohne W: **Interaction of the capsid protein p24 (HIV-1) with sequence-derived peptides: influence on p24 dimerization.** *Virology* 1999, **254:**6-10.

52. Agopian A, Gros E, Aldrian-Herrada G, Bosquet N, Clayette P, Divita G: **A new generation of peptide-based inhibitors targeting HIV-1 reverse transcriptase conformational flexibility.** *J Biol Chem* 2009, **284:**254-264.
53. Depollier J, Hourdou ML, Aldrian-Herrada G, Rothwell P, Restle T, Divita G: **Insight into the mechanism of a peptide inhibitor of HIV reverse transcriptase dimerization.** *Biochemistry* 2005, **44:**1909-1918.
54. Morris MC, Robert-Hebmann V, Chaloin L, Mery J, Heitz F, Devaux C, Goody RS, Divita G: **A new potent HIV-1 reverse transcriptase inhibitor. A synthetic peptide derived from the interface subunit domains.** *J Biol Chem* 1999, **274:**24941-24946.
55. Divita G, Restle T, Goody RS, Chermann JC, Baillon JG: **Inhibition of human immunodeficiency virus type 1 reverse transcriptase dimerization using synthetic peptides derived from the connection domain.** *J Biol Chem* 1994, **269:**13080-13083.
56. Broglia RA, Provasi D, Vasile F, Ottolina G, Longhi R, Tiana G: **A folding inhibitor of the HIV-1 protease.** *Proteins* 2006, **62:**928-933.
57. Bonomi M, Gervasio FL, Tiana G, Provasi D, Broglia RA, Parrinello M: **Insight into the folding inhibition of the HIV-1 protease by a small peptide.** *Biophys J* 2007, **93:**2813-2821.
58. Broglia RA, Tiana G, Sutto L, Provasi D, Simona F: **Design of HIV-1-PR inhibitors that do not create resistance: blocking the folding of single monomers.** *Protein Sci* 2005, **14:**2668-2681.
59. Bonomi M, Barducci A, Gervasio FL, Parrinello M: **Multiple routes and milestones in the folding of HIV-1 protease monomer.** *PLoS One* 2010, **5:**e13208.
60. Bowman MJ, Chmielewski J: **Novel strategies for targeting the dimerization interface of HIV protease with cross-linked interfacial peptides.** *Biopolymers* 2002, **66:**126-133.
61. Narumi T, Komoriya M, Hashimoto C, Wu H, Nomura W, Suzuki S, Tanaka T, Chiba J, Yamamoto N, Murakami T, Tamamura H: **Conjugation of cell-penetrating peptides leads to identification of anti-HIV peptides from matrix proteins.** *Bioorg Med Chem* 2012, **20:**1468-1474.
62. Oz Gleenberg I, Avidan O, Goldgur Y, Herschhorn A, Hizi A: **Peptides derived from the reverse transcriptase of human immunodeficiency virus type 1 as novel inhibitors of the viral integrase.** *J Biol Chem* 2005, **280:**21987-21996.
63. Zawahir Z, Neamati N: **Inhibition of HIV-1 integrase activity by synthetic peptides derived from the HIV-1 HXB2 Pol region of the viral genome.** *Bioorg Med Chem Lett* 2006, **16:**5199-5202.
64. Oz Gleenberg I, Herschhorn A, Goldgur Y, Hizi A: **Inhibition of human immunodeficiency virus type-1 reverse transcriptase by a novel peptide derived from the viral integrase.** *Arch Biochem Biophys* 2007, **458:**202-212.
65. Gleenberg IO, Herschhorn A, Hizi A: **Inhibition of the activities of reverse transcriptase and integrase of human immunodeficiency virus type-1 by peptides derived from the homologous viral protein R (Vpr).** *J Mol Biol* 2007, **369:**1230-1243.

66. Suzuki S, Maddali K, Hashimoto C, Urano E, Ohashi N, Tanaka T, Ozaki T, Arai H, Tsutsumi H, Narumi T, et al: **Peptidic HIV integrase inhibitors derived from HIV gene products: structure-activity relationship studies.** *Bioorg Med Chem* 2010, **18:**6771-6775.
67. Suzuki S, Urano E, Hashimoto C, Tsutsumi H, Nakahara T, Tanaka T, Nakanishi Y, Maddali K, Han Y, Hamatake M, et al: **Peptide HIV-1 integrase inhibitors from HIV-1 gene products.** *J Med Chem* 2010, **53:**5356-5360.
68. Baraz L, Friedler A, Blumenzweig I, Nussinuv O, Chen N, Steinitz M, Gilon C, Kotler M: **Human immunodeficiency virus type 1 Vif-derived peptides inhibit the viral protease and arrest virus production.** *FEBS Lett* 1998, **441:**419-426.
69. Friedler A, Blumenzweig I, Baraz L, Steinitz M, Kotler M, Gilon C: **Peptides derived from HIV-1 Vif: a non-substrate based novel type of HIV-1 protease inhibitors.** *J Mol Biol* 1999, **287:**93-101.
70. Potash MJ, Bentsman G, Muir T, Krachmarov C, Sova P, Volsky DJ: **Peptide inhibitors of HIV-1 protease and viral infection of peripheral blood lymphocytes based on HIV-1 Vif.** *Proc Natl Acad Sci U S A* 1998, **95:**13865-13868.
71. Louis JM, Dyda F, Nashed NT, Kimmel AR, Davies DR: **Hydrophilic peptides derived from the transframe region of Gag-Pol inhibit the HIV-1 protease.** *Biochemistry* 1998, **37:**2105-2110.
72. Davis DA, Brown CA, Singer KE, Wang V, Kaufman J, Stahl SJ, Wingfield P, Maeda K, Harada S, Yoshimura K, et al: **Inhibition of HIV-1 replication by a peptide dimerization inhibitor of HIV-1 protease.** *Antiviral Res* 2006, **72:**89-99.
73. Davis DA, Tebbs IR, Daniels SI, Stahl SJ, Kaufman JD, Wingfield P, Bowman MJ, Chmielewski J, Yarchoan R: **Analysis and characterization of dimerization inhibition of a multi-drug-resistant human immunodeficiency virus type 1 protease using a novel size-exclusion chromatographic approach.** *Biochem J* 2009, **419:**497-506.
74. Rosenbluh J, Hayouka Z, Loya S, Levin A, Armon-Omer A, Britan E, Hizi A, Kotler M, Friedler A, Loyter A: **Interaction between HIV-1 Rev and integrase proteins: a basis for the development of anti-HIV peptides.** *J Biol Chem* 2007, **282:**15743-15753.
75. Levin A, Rosenbluh J, Hayouka Z, Friedler A, Loyter A: **Integration of HIV-1 DNA is regulated by interplay between viral rev and cellular LEDGF/p75 proteins.** *Mol Med* 2010, **16:**34-44.
76. Liu F, Boross PI, Wang YF, Tozser J, Louis JM, Harrison RW, Weber IT: **Kinetic, stability, and structural changes in high-resolution crystal structures of HIV-1 protease with drug-resistant mutations L24I, I50V, and G73S.** *J Mol Biol* 2005, **354:**789-800.
77. Veljkovic N, Branch DR, Metlas R, Prljic J, Manfredi R, Stringer WW, Veljkovic V: **Antibodies reactive with C-terminus of the second conserved region of HIV-1gp120 as possible prognostic marker and therapeutic agent for HIV disease.** *J Clin Virol* 2004, **31 Suppl 1:**S39-44.
78. Franke R, Hirsch T, Overwin H, Eichler J: **Synthetic mimetics of the CD4 binding site of HIV-1 gp120 for the design of immunogens.** *Angew Chem Int Ed Engl* 2007, **46:**1253-1255.

79. Carlier E, Mabrouk K, Moulard M, Fajloun Z, Rochat H, De Waard M, Sabatier JM: **Ion channel activation by SPC3, a peptide derived from the HIV-1 gp120 V3 loop.** *J Pept Res* 2000, **56:**427-437.
80. Sakaida H, Hori T, Yonezawa A, Sato A, Isaka Y, Yoshie O, Hattori T, Uchiyama T: **T-tropic human immunodeficiency virus type 1 (HIV-1)-derived V3 loop peptides directly bind to CXCR-4 and inhibit T-tropic HIV-1 infection.** *J Virol* 1998, **72:**9763-9770.
81. Haynes BF, Ma B, Montefiori DC, Wrin T, Petropoulos CJ, Sutherland LL, Scearce RM, Denton C, Xia SM, Korber BT, Liao HX: **Analysis of HIV-1 subtype B third variable region peptide motifs for induction of neutralizing antibodies against HIV-1 primary isolates.** *Virology* 2006, **345:**44-55.
82. Zolla-Pazner S, Cohen S, Pinter A, Krachmarov C, Wrin T, Wang S, Lu S: **Cross-clade neutralizing antibodies against HIV-1 induced in rabbits by focusing the immune response on a neutralizing epitope.** *Virology* 2009, **392:**82-93.
83. Moseri A, Tantry S, Sagi Y, Arshava B, Naider F, Anglister J: **An optimally constrained V3 peptide is a better immunogen than its linear homolog or HIV-1 gp120.** *Virology* 2010, **401:**293-304.
84. Chertov O, Zhang N, Chen X, Oppenheim JJ, Lubkowski J, McGrath C, Sowder RC, 2nd, Crise BJ, Malyguine A, Kutzler MA, et al: **Novel peptides based on HIV-1 gp120 sequence with homology to chemokines inhibit HIV infection in cell culture.** *PLoS One* 2011, **6:**e14474.
85. Marchio S, Alfano M, Primo L, Gramaglia D, Butini L, Gennero L, De Vivo E, Arap W, Giacca M, Pasqualini R, Bussolino F: **Cell surface-associated Tat modulates HIV-1 infection and spreading through a specific interaction with gp120 viral envelope protein.** *Blood* 2005, **105:**2802-2811.
86. De Houwer S, Demeulemeester J, Thys W, Taltynov O, Christ F, Debyser Z: **Identification of residues in the C-terminal domain of HIV-1 integrase that mediate binding to TRN-SR2.** *J Biol Chem* 2012.
87. Armon-Omer A, Graessmann A, Loyter A: **A synthetic peptide bearing the HIV-1 integrase 161-173 amino acid residues mediates active nuclear import and binding to importin alpha: characterization of a functional nuclear localization signal.** *J Mol Biol* 2004, **336:**1117-1128.
88. Keogan S, Passic S, Krebs FC: **Infection by CXCR4-Tropic Human Immunodeficiency Virus Type 1 Is Inhibited by the Cationic Cell-Penetrating Peptide Derived from HIV-1 Tat.** *Int J Pept* 2012, **2012:**349427.
89. Friedler A, Friedler D, Luedtke NW, Tor Y, Loyter A, Gilon C: **Development of a functional backbone cyclic mimetic of the HIV-1 Tat arginine-rich motif.** *J Biol Chem* 2000, **275:**23783-23789.
90. Xiao H, Neuveut C, Tiffany HL, Benkirane M, Rich EA, Murphy PM, Jeang KT: **Selective CXCR4 antagonism by Tat: implications for in vivo expansion of coreceptor use by HIV-1.** *Proc Natl Acad Sci U S A* 2000, **97:**11466-11471.
91. Ghezzi S, Noonan DM, Aluigi MG, Vallanti G, Cota M, Benelli R, Morini M, Reeves JD, Vicenzi E, Poli G, Albini A: **Inhibition of CXCR4-dependent HIV-1 infection by extracellular HIV-1 Tat.** *Biochem Biophys Res Commun* 2000, **270:**992-996.

92. Mills NL, Daugherty MD, Frankel AD, Guy RK: **An alpha-helical peptidomimetic inhibitor of the HIV-1 Rev-RRE interaction.** *J Am Chem Soc* 2006, **128:**3496-3497.
93. Jain S, Rosenthal KL: **The gp41 epitope, QARVLAVERY, is highly conserved and a potent inducer of IgA that neutralizes HIV-1 and inhibits viral transcytosis.** *Mucosal Immunol* 2011, **4:**539-553.
94. Pornillos O, Ganser-Pornillos BK, Kelly BN, Hua Y, Whitby FG, Stout CD, Sundquist WI, Hill CP, Yeager M: **X-ray structures of the hexameric building block of the HIV capsid.** *Cell* 2009, **137:**1282-1292.
95. Daugherty MD, Liu B, Frankel AD: **Structural basis for cooperative RNA binding and export complex assembly by HIV Rev.** *Nat Struct Mol Biol* 2010, **17:**1337-1342.
96. Auclair JR, Green KM, Shandilya S, Evans JE, Somasundaran M, Schiffer CA: **Mass spectrometry analysis of HIV-1 Vif reveals an increase in ordered structure upon oligomerization in regions necessary for viral infectivity.** *Proteins* 2007, **69:**270-284.
97. Levy ED: **A simple definition of structural regions in proteins and its use in analyzing interface evolution.** *J Mol Biol* 2010, **403:**660-670.
