# Supplementary material for: An integrated map of HIV genome-wide variation from a population perspective
Source: Retrovirology. 2015 Feb 15;12:18. doi: 10.1186/s12977-015-0148-6 (PMC4358901; doi:10.1186/s12977-015-0148-6)
Supplement: Additional file 3: — Software. Our Matlab toolbox developed for data visualization, genomic diversity analysis and HIV genomic alignment. Our sequence datasets are also included. [file 12977_2015_148_MOESM3_ESM.zip › HIVToolboxManual.pdf]

Supplementary Software:

An integrated map of HIV genome-wide variation from a population perspective

Guangdi Li[1,2], Supinya Piampongsant[2], Nuno Rodrigues Faria[3], Arnout Voet[4], Andrea-Clemencia Pineda-Peña[2,5], Ricardo Khouri[2,6], Philippe Lemey[2], Anne-Mieke Vandamme[2,7], Kristof Theys [2]

[1] Metabolic Syndrome Research Center, the Second Xiangya Hospital, Central South University, Changsha, Hunan, China

[2] Rega Institute for Medical Research, Department of Microbiology and Immunology, KU Leuven, Leuven, Belgium

[3] Department of Zoology, University of Oxford, Oxford OX1-3PS, UK

[4] Zhang IRU, RIKEN Institute Laboratories, Hirosawa 2-1, Wako-shi, Saitama, Japan

[5] Clinical and Molecular Infectious Disease Group, Faculty of Sciences and Mathematics, Universidad del Rosario, Bogotá, Colombia

[6] LIMI-LIP, Centro de Pesquisa Gonçalo Moniz, FIOCRUZ, Salvador-Bahia, Brasil

[7] Centro de Malária e Outras Doenças Tropicais and Unidade de Microbiologia, Instituto de Higiene e Medicina Tropical, Universidade Nova de Lisboa, Lisbon, Portugal

*: Corresponding author (liguangdi.research@gmail.com)

1. Introduction

Extensive full-length sequences of HIV genomes have been accumulated in the past few years. To our best knowledge, a toolbox developed for analyzing full-length HIV genomes has not been reported. Here, we offer a Matlab toolbox for HIV full-length genomic analysis. We provide an alignment tool that optimizes amino acid alignments given the input files of nucleotide genomic sequences. Classical alignment tools either perform nucleotide or amino acid alignments when nucleotide (or amino acid) sequences are optimized given nucleotide (or amino acid) sequences as inputs. Most classical tools do not optimize amino acid alignments based on nucleotide genomic sequences, because of overlapping open reading frames. Considering different lengths and locations of 15 proteins in 3 open reading frames within the HIV genome, our tool has been developed to optimize the genomic alignment using the reference mapping strategy.

This document describes the manual of functions and algorithms in our software. We present the software for (1) full-length genome alignment, (2) the intra- and inter-clade genomic diversity analysis and (3) data visualization. Our example datasets are available in the toolbox. We implemented our toolbox in Matlab 2013a under the Ubuntu system V14.04. A simple example to use our toolbox is provided in the document named: "MeasureGenomeGeneticDiversity.m". If one encountered the difficulty of using the toolbox in different Matlab versions or computer systems, please email Guangdi Li (liguangdi.research@gmail.com) for more information.

2. Alignment of full-length HIV genome

Both HIV-1 and HIV-2 genomes encode 15 proteins in three open reading frames. The number of nucleotides in the coding regions of one HIV genomic sequence is usually between 8500 and 8800. We have developed a tool to improve codon sequence alignments. Briefly, the input of our toolbox requires the nucleotide sequence alignment, which can be prepared using many classical nucleotide alignment tools (e.g. SeaView [1], Mega [2], Mafft [3]). Next, our toolbox uses the reference genome to assemble the nucleotide sequence alignment from each protein coding region. Thereafter, the alignments in protein regions are optimized by maximizing the matched codons based on the amino acid substitution matrix.

We first describe our algorithm which improves amino acid alignments from nucleotide sequences in one protein coding region.

Algorithm: Alignment of codon sequences
Input: One MSA file with nucleotide genomic sequences
Output: One MSA file with aligned amino acid sequences
Step 1: Arrange nucleotide positions into codon triples. /* --A-B---C--- => --ABC------- */
Step 2: Refine small nucleotides into codon positions. /* -AAA--BBB- => --AAA--BBB-- */
/* -AAA-BBB-- => --AAA--BBB-- */
Step 3: Refine codon positions to the left or right side if substitution scores are improved.
Step 4: Optimize the codon alignments at the insertion and deletion regions.
Step 5: Transform codon sequence alignments into amino acid alignments.

In our toolbox, the function called "TransferNucleotide2AminoAcidAlignment.m" implements the above algorithm. A simple example is given to run the alignment tool. Note that MSAInputFile is the path of the MSA input file and MSAOutputFile is the path of the MSA output file.

Example:
```
[ Seq,SeqTitle,SeqID ] = ExtractSequenceOut(  MSAInputFile  ); % collect the nucleotide input
Seq = TransferNucleotide2AminoAcidAlignment( Seq,SeqTitle ); % optimize codon alignments
[a,b] = WriteSequence2Fasta( Seq, SeqTitle, MSAOutputFile  ); % output improved alignments
```

Secondly, six steps are performed to align full-length HIV genomic sequences. An example is provided in the file called "StartFile_FullGnomeAlignmentTool.m".

Procedure for HIV genomic alignment:
(1) We collect the information of HXB2 genomic reference.
(2) We concatenate each HIV-1 protein region in the full-length genome based on HXB2.
(3) We perform the codon sequence alignment of HIV-1 proteins using full-length alignment tool.
(4) We examine sequence quality using other sequence visualization software (e.g. Seaview).
(5) We transform the sequences from nucleotide forms to amino acid forms.
(6) We assemble the concatenated protein sequences into full-length amino acid genomes.

Step 1: HIV full-length genomic information

%% protein information of HIV-1 genome, based on HXB2 reference strain
(see website: http://www.hiv.lanl.gov/content/sequence/HIV/MAP/landmark.html)

LocPronRegion = { 'P17', 'P24', 'P2', 'P7', 'P1','P6', 'Protease','P51', 'P31', 'Vif', 'Vpr', 'Tat', 'Rev', 'Vpu', 'GP120', 'GP41', 'Nef' };
HIVProteinRegionName = { 'Matrix', 'Capsid', 'p2', 'Nucleocapsid', 'p1', 'p6', 'Protease', 'RT', 'Integrase', 'Vif', 'Vpr', 'Tat', 'Rev', 'Vpu', 'GP120', 'GP41', 'Nef' };
ProteinNumber = length(HIVProteinRegionName);

%% stop codon information
StopCodonProtein = [0 0 0 0 0 1 0   0 1 1 1 1 1   1 0 1 1];

%% gene information whether a translated protein has two genes in different open reading frames
Protein_Gene =   [1 1 1 1 1 1 1   1 1 1 1 2 2 1 1 1 1];

%% the start position of the gene in individual open reading frames
LocationLowBound =[790, 1186, 1879, 1921, 2086,
2134, 2253, 2550, 4230, 5041, 5559, 5831, 8379, 5970, 8379, 6062, 6315, 7758, 8797];

%% the end position of the gene in individual open reading frames
LocationUpperBound = [1185, 1878, 1920, 2085, 2133, 2292, 2549, 4229, 5096, 5619, 5850, 6045, 8469, 6045, 8653, 6310, 9417, 6045, 8469];

%% the length of amino acid sequences
ProteinLen = [132, 231, 14, 55, 16, 52, 99, 560, 288, 192, 96, 101, 116, 82, 481, 345, 206];

% HIV-2 genomic information
% ProteinRegionName ={'Matrix', 'Capsid', 'p2', 'Nucleocapsid', 'p1', 'p6', 'Protease', 'RT', 'Integrase', 'Vif', 'Vpr',   'Tat',   'Rev'   'Vpx','GP120','GP41','Nef'};

% LocationLowBound =[1053, 1458, 2145, 2196, 2352, 2394, 2555, 2852, 4529, 5340, 6151, 6302, 8806, 6528, 8806, 5812, 6670, 8179, 9077];
% LocationUpperBound=[1457, 2144, 2195, 2351, 2393, 2585, 2851, 4528, 5407, 5984, 6456, 6597, 8899, 6597, 9056, 6150, 8178, 9240, 9865];

%% output files of aligned sequences will be saved in the directory called: TemporaryData
OutputDir = [currentFolder '/TemporaryData/'];

Step 2: sequences in each HIV-1 protein region

%% concatenate each HIV-1 protein region in the full-length genome based on HXB2.
if 0 %% if you choose to run this step, set the value 1 here, otherwise 0.
CurrentStep = 'Step 1'
% make sure the first sequence of fasta input is the reference HXB2.
InputDir = [currentFolder '/ExampleData/B_PureSubtype.fasta'];
% extract fasta sequences of each protein region, return sequences, sequence titles, sequence IDs

```
[ Seq,SeqTitle,SeqID ] = ExtractSequenceOut( InputDir );
    [ Row,Col ]= size( Seq );
    FullGenomeSequence = repmat( '-',Row,10000); Index = 0;
    FirstSeq = Seq(1,:); Pos = find(FirstSeq~='-');  No = 0;
    for p = 1:ProteinNumber
        IndexSet =[];
        for q = 0:Protein_Gene(p)-1 %% define the protein region "[Start - End]"
             Start= Pos( LocationLowBound(p+q+No) ); End=Pos( LocationUpperBound(p+q+No) );
             IndexSet = [IndexSet Start:End];
        end
        No = No + Protein_Gene(p) - 1;
        LocalSeq = Seq( :,IndexSet );
        if StopCodonProtein(p) == 0
            FullGenomeSequence( :,1+Index:Index+length(IndexSet) ) = LocalSeq ;
            Index = Index + length(IndexSet);
        else
           FullGenomeSequence(:,(1+Index):(Index+length(IndexSet)-
3))=LocalSeq( :,1:(length(IndexSet)-3) ) ;
           Index = Index + length(IndexSet) - 3;
        end
        % write out the fasta files into the output directory called: "TemporaryData".
        [a,b] = WriteSequence2Fasta( LocalSeq,SeqTitle, [OutputDir HIVProteinRegionName{p}
'.fasta' ] );
        fclose( 'all' );
    end
end
```

Step 3: multiple sequence alignment of HIV-1 subtype B genome

```
% improve the alignment of nucleotide sequences by amino acid sequence alignment
if 1 %% if you choose to run this step, set the value 1 here, otherwise 0.
    CurrentStep = 'Step 2'
    for p = 16:17%ProteinNumber
        % for each protein, we transform nucleotide sequences to codon sequences
        LocalProtein = [ OutputDir HIVProteinRegionName{p} '.fasta' ]
        [ Seq,SeqTitle,SeqID ] = ExtractSequenceOut( LocalProtein );

        %% if there are many gaps and insertions in the sequences, the program may meet
        %% difficult to identify the ideal codon residues, please check the temporary fasta
        %% outputs, named: "Tran1.fasta" to "Tran18.fasta".
        Seq = TransferNucleotide2AminoAcidAlignment( Seq,SeqTitle );

        % output the aligned sequences into the directory called "TemporaryData".
        [a,b] = WriteSequence2Fasta( Seq, SeqTitle, [ OutputDir HIVProteinRegionName{p}
'_CodonAlignment.fasta' ] );
    end
```

end

Step 4: improvement of sequence alignment by manual inspection

```
%Seaview manual examination: the aligned sequences are available in the fold: TemporaryData
if 0 %% if you choose to run this step, set the value 1 here, otherwise 0.
CurrentStep = 'Step 3'
% move sequences in a small region from left to right
[ CurrentSeq,SeqTitle,SeqID ] = ExtractSequenceOut( [ currentFolder
'/TemporaryData/p6_CodonAlignment.fasta' ] );
CurrentSeq = MoveSeqLeft2Right( CurrentSeq,40,57 ); %% move the regions between the
position 40 and 57 from left to right side

% move sequences in a small region from right to left
CurrentSeq = MoveSeqRight2Left( CurrentSeq,133,138 ); %% move the regions between the
position 133 and 138 from right to left side

[ a,b ] = WriteSequence2Fasta( CurrentSeq, SeqTitle, [ currentFolder
'/TemporaryData/p6_CodonAlignment_Improve.fasta' ] );
fclose( 'all' );
end
```

Step 5: transform sequences from nucleotide to amino acid forms

```
if 1 %% if you choose to run this step, set the value 1 here, otherwise 0.
 CurrentStep = 'Step 4'
 for p = 12:ProteinNumber
     %% transform the nucleotide to amino acid sequences
     if fopen([ OutputDir HIVProteinRegionName{p} '_CodonAlignment_Improve.fasta' ],'r')>0
        LocalProtein = [ OutputDir HIVProteinRegionName{p} '_CodonAlignment_Improve.fasta' ]
     else
       LocalProtein = [ OutputDir HIVProteinRegionName{p} '_CodonAlignment.fasta' ]
     end
     [ Seq,SeqTitle,SeqID ] = ExtractSequenceOut( LocalProtein );
     [a,b] = WriteSequence2Fasta( Nucleotide2AA(Seq), SeqTitle, [ OutputDir
HIVProteinRegionName{p} '_AminoAcidAlignment.fasta' ] );
 end
end
```

Step 6: assemble protein sequences into full-length genomes

```
if 1
    CurrentStep = 'Step 5'
    [ Seq,SeqTitle,SeqID ] = ExtractSequenceOut( [ OutputDir HIVProteinRegionName{1}
'_AminoAcidAlignment.fasta' ] );
    [ Row,Col ] = size( Seq );
```

```
    FullGenome = repmat('-',Row,10000); Increase = Col;
    FullGenome(:,1:Col) = Seq;
    for p = 2:ProteinNumber
        [ Seq, SeqTitle, SeqID ] = ExtractSequenceOut( [ OutputDir HIVProteinRegionName{ p }
'_AminoAcidAlignment.fasta' ] );
        [ Row, Col ] = size( Seq );
        FullGenome( :,(Increase+1):(Increase+Col) ) = Seq;
        Increase = Increase + Col;
    end
    FullGenome = FullGenome( : , 1:Increase );
    [a,b] = WriteSequence2Fasta( FullGenome, SeqTitle, [ OutputDir
'BGenome_AminoAcidAlignment.fasta' ] );
end
```

3. Genome-wide genetic diversity

Sequence diversity was calculated based on the pairwise nucleotide (NT) and amino acid (AA) comparisons [4, 5]. When calculating the amino acid diversity of HIV genome, we concatenated the amino acid sequences of 15 HIV protein coding regions in the full-length genome. Suppose the sequence dataset D contains L sequences with N positions, genetic diversity at position n is calculated by: $GD(D_n) = 1 - \frac{2}{L(L-1)} \sum_{i=1}^{L} \sum_{j=i+1}^{L} \delta(D_{n,i} = D_{n,j})$, where $D_{n,i}$ is the NT or AA form of the position n at the ith sequence in the dataset D, δ represents the Kronecker symbol, $\delta(D_{n,i} = D_{n,j})$ equals 1 if $D_{n,i}$ is identical to $D_{n,j}$; otherwise 0. Given the sequence dataset D, intra-clade genetic diversity $AGD(D)$ is defined as the average genetic diversity of all positions: $AGD(D) = 1 - \frac{1}{N} \sum_{n=1}^{N} \frac{2}{L(L-1)} \sum_{i=1}^{L} \sum_{j=i+1}^{L} \delta(D_{n,i} = D_{n,j})$. Suppose two sequence datasets D1 and D2 aligned with the same reference genome have the number of sequences L_1 and L_2 respectively. The inter-clade genetic diversity between D1 and D2 is defined as: $RGD(D1, D2) = 1 - \frac{1}{N} \sum_{n=1}^{N} \frac{1}{L_1 \times L_2} \sum_{i=1}^{L_1} \sum_{j=1}^{L_2} \delta(D1_{n,i} = D2_{n,j})$. Furthermore, only positions for which less than 20% of sequences had gaps were considered and gaps were treated as missing data. Intra- and inter-clade genetic diversity was measured using one genomic sequence per patient, while intra-patient diversity was calculated using more than one genomic sequence sampled from individual patients. The Mann–Whitney U test was performed to compare the distributions of genetic diversity and a significant difference was identified if a p-value was less than 0.05.

“AnalysisGenomeIntraSubtypeDiversity.m” implements the computation of the intra-clade genomic diversity. “AnalysisGenomeInterSubtypeDiversity.m” implements the computation of the inter-clade genomic diversity.

4. Data visualization

In our study, we developed a toolbox for data visualization. We provide the Matlab codes to visualize the distribution of genomic diversity. For the visualization in Figure 1, the data was visualized by creating the probability bins given a user-defined cutoff, indicated in the code. For the Figure 2, the genetic diversity was visualized by sliding windows. Please see examples and details in the file called: “Visualization_GenomicDiversity.m”.

References

1. Gouy M, Guindon S, Gascuel O: **SeaView version 4: A multiplatform graphical user interface for sequence alignment and phylogenetic tree building.** *Mol Biol Evol* 2010, **27:**221-224.
2. Tamura K, Stecher G, Peterson D, Filipski A, Kumar S: **MEGA6: Molecular Evolutionary Genetics Analysis version 6.0.** *Mol Biol Evol* 2013, **30:**2725-2729.
3. Edgar RC: **MUSCLE: multiple sequence alignment with high accuracy and high throughput.** *Nucleic Acids Res* 2004, **32:**1792-1797.
4. Li G, Verheyen J, Rhee SY, Voet A, Vandamme AM, Theys K: **Functional conservation of HIV-1 gag: implications for rational drug design.** *Retrovirology* 2013, **10:**126.
5. Spira S, Wainberg MA, Loemba H, Turner D, Brenner BG: **Impact of clade diversity on HIV-1 virulence, antiretroviral drug sensitivity and drug resistance.** *J Antimicrob Chemother* 2003, **51:**229-240.
